# Supplementary material for: Deprescribing interventions in older adults: An overview of systematic reviews
Source: PLoS One. 2024 Jun 17;19(6):e0305215. doi: 10.1371/journal.pone.0305215 (PMC11182547; doi:10.1371/journal.pone.0305215)
Supplement: S1 Checklist — (DOCX) [file pone.0305215.s001.docx]

| **Section and Topic** | **Item #** | **Checklist item** | **Location where item is reported** |
| --- | --- | --- | --- |
| **TITLE** | | |  |
| Title | 1 | Identify the report as a systematic review. | Line 1-2 (defined as an overview review, a specific type of systematic review. |
| **ABSTRACT** | | |  |
| Abstract | 2 | See the PRISMA 2020 for Abstracts checklist. | See supporting information file |
| **INTRODUCTION** | | |  |
| Rationale | 3 | Describe the rationale for the review in the context of existing knowledge. | Lines 101-108 |
| Objectives | 4 | Provide an explicit statement of the objective(s) or question(s) the review addresses. | Lines 109-113 |
| **METHODS** | | |  |
| Eligibility criteria | 5 | Specify the inclusion and exclusion criteria for the review and how studies were grouped for the syntheses. | Inclusion: Table 1 (line 137)  Exclusion: Lines 132-134 |
| Information sources | 6 | Specify all databases, registers, websites, organisations, reference lists and other sources searched or consulted to identify studies. Specify the date when each source was last searched or consulted. | Lines 120-128. Dates searched 1/1/2005-3/16/23 |
| Search strategy | 7 | Present the full search strategies for all databases, registers and websites, including any filters and limits used. | S1 Appendix |
| Selection process | 8 | Specify the methods used to decide whether a study met the inclusion criteria of the review, including how many reviewers screened each record and each report retrieved, whether they worked independently, and if applicable, details of automation tools used in the process. | Lines 153-166.  Inclusion: Table 1 (line 136)  Exclusion: Lines 144-145  No automation tools were used in the selection process |
| Data collection process | 9 | Specify the methods used to collect data from reports, including how many reviewers collected data from each report, whether they worked independently, any processes for obtaining or confirming data from study investigators, and if applicable, details of automation tools used in the process. | Lines 153-166.  No automation tools were used in the process |
| Data items | 10a | List and define all outcomes for which data were sought. Specify whether all results that were compatible with each outcome domain in each study were sought (e.g. for all measures, time points, analyses), and if not, the methods used to decide which results to collect. | Table 2 (line 145) |
|  | 10b | List and define all other variables for which data were sought (e.g. participant and intervention characteristics, funding sources). Describe any assumptions made about any missing or unclear information. | Line numbers 157-165 |
| Study risk of bias assessment | 11 | Specify the methods used to assess risk of bias in the included studies, including details of the tool(s) used, how many reviewers assessed each study and whether they worked independently, and if applicable, details of automation tools used in the process. | Line numbers 167-173 and supporting information (S5 Table) for the quality assessment.  No automation tools were used in the assessment process. |
| Effect measures | 12 | Specify for each outcome the effect measure(s) (e.g. risk ratio, mean difference) used in the synthesis or presentation of results. | Not applicable due to overview of reviews |
| Synthesis methods | 13a | Describe the processes used to decide which studies were eligible for each synthesis (e.g. tabulating the study intervention characteristics and comparing against the planned groups for each synthesis (item #5)). | Not applicable due to overview of reviews |
|  | 13b | Describe any methods required to prepare the data for presentation or synthesis, such as handling of missing summary statistics, or data conversions. | Not applicable due to overview of reviews |
|  | 13c | Describe any methods used to tabulate or visually display results of individual studies and syntheses. | Not applicable due to overview of reviews |
|  | 13d | Describe any methods used to synthesize results and provide a rationale for the choice(s). If meta-analysis was performed, describe the model(s), method(s) to identify the presence and extent of statistical heterogeneity, and software package(s) used. | Lines 175-187. |
|  | 13e | Describe any methods used to explore possible causes of heterogeneity among study results (e.g. subgroup analysis, meta-regression). | Not applicable due to overview of reviews |
|  | 13f | Describe any sensitivity analyses conducted to assess robustness of the synthesized results. | Not applicable due to overview of reviews |
| Reporting bias assessment | 14 | Describe any methods used to assess risk of bias due to missing results in a synthesis (arising from reporting biases). | Not applicable due to overview of reviews |
| Certainty assessment | 15 | Describe any methods used to assess certainty (or confidence) in the body of evidence for an outcome. | Not applicable due to overview of reviews |
| **RESULTS** | | |  |
| Study selection | 16a | Describe the results of the search and selection process, from the number of records identified in the search to the number of studies included in the review, ideally using a flow diagram. | Line numbers 194-196;  Flowchart, see Fig 1 |
|  | 16b | Cite studies that might appear to meet the inclusion criteria, but which were excluded, and explain why they were excluded. | Line numbers 195-196  See supplementary S3 Table |
| Study characteristics | 17 | Cite each included study and present its characteristics. | See supplementary file |
| Risk of bias in studies | 18 | Present assessments of risk of bias for each included study. | Line numbers 225-231. Supplementary file S5 Table |
| Results of individual studies | 19 | For all outcomes, present, for each study: (a) summary statistics for each group (where appropriate) and (b) an effect estimate and its precision (e.g. confidence/credible interval), ideally using structured tables or plots. | Not applicable since the overview of systematic reviews and meta-analyses |
| Results of syntheses | 20a | For each synthesis, briefly summarise the characteristics and risk of bias among contributing studies. | Line numbers 225-231. Supplementary file S5 Table |
|  | 20b | Present results of all statistical syntheses conducted. If meta-analysis was done, present for each the summary estimate and its precision (e.g. confidence/credible interval) and measures of statistical heterogeneity. If comparing groups, describe the direction of the effect. | Not applicable due to overview of reviews |
|  | 20c | Present results of all investigations of possible causes of heterogeneity among study results. | Not applicable due to overview of reviews |
|  | 20d | Present results of all sensitivity analyses conducted to assess the robustness of the synthesized results. | Not applicable due to overview of reviews |
| Reporting biases | 21 | Present assessments of risk of bias due to missing results (arising from reporting biases) for each synthesis assessed. | Not applicable due to overview of reviews |
| Certainty of evidence | 22 | Present assessments of certainty (or confidence) in the body of evidence for each outcome assessed. | Not applicable due to overview of reviews |
| **DISCUSSION** | | |  |
| Discussion | 23a | Provide a general interpretation of the results in the context of other evidence. | Line number 379-92 |
|  | 23b | Discuss any limitations of the evidence included in the review. | Line number 465-467 |
|  | 23c | Discuss any limitations of the review processes used. | Line number 457-473 |
|  | 23d | Discuss implications of the results for practice, policy, and future research. | Line number 475-485 |
| **OTHER INFORMATION** | | |  |
| Registration and protocol | 24a | Provide registration information for the review, including register name and registration number, or state that the review was not registered. | PROSPERO registration CRD42020178860 |
|  | 24b | Indicate where the review protocol can be accessed, or state that a protocol was not prepared. | The PROSPERO protocol can be accessed via above mentioned registration and is a hyperlink in the manuscript |
|  | 24c | Describe and explain any amendments to information provided at registration or in the protocol. | Supplementary information S1 Table |
| Support | 25 | Describe sources of financial or non-financial support for the review, and the role of the funders or sponsors in the review. | This study was supported by National Institute on Aging (NIA: 1R24AG064025, MPI Steinman, Boyd; SG Co-investigator). www.nia.nih.gov. The views expressed are those of the author(s) and not necessarily those of the NIA. The funders had no role in considering the study design or in the collection, analysis, and interpretation of data, the writing of the report, or the decision to submit the article for publication.  ER and AL was supported by an Australian National Health and Medical Research Council (NHMRC) Investigator Grant (APP1195460). NM was supported by Health Research Board Collaboration in Ireland for Clinical Effectiveness Reviews Award (HRB-CICER-2016-1871). |
| Competing interests | 26 | Declare any competing interests of review authors. | The authors declare that they have no competing interests. Declared in PLOS One Editorial system |
| Availability of data, code and other materials | 27 | Report which of the following are publicly available and where they can be found: template data collection forms; data extracted from included studies; data used for all analyses; analytic code; any other materials used in the review. | See Supplementary files S2-S7 Tables. |

*From:*  Page MJ, McKenzie JE, Bossuyt PM, Boutron I, Hoffmann TC, Mulrow CD, et al. The PRISMA 2020 statement: an updated guideline for reporting systematic reviews. BMJ 2021;372:n71. doi: 10.1136/bmj.n71

For more information, visit: <http://www.prisma-statement.org/>
