# Supplementary material for: Deprescribing interventions in older adults: An overview of systematic reviews
Source: PLoS One. 2024 Jun 17;19(6):e0305215. doi: 10.1371/journal.pone.0305215 (PMC11182547; doi:10.1371/journal.pone.0305215)
Supplement: S1 Appendix — (DOCX) [file pone.0305215.s002.docx]

**S1 Appendix. Search Strategy and Databases**

Databases: Medline (Ebsco), Embase (Elsevier), CINAHL Complete (Ebsco), APA PsycInfo (Ebsco), Scopus (Elsevier), Web of Science Core Collection (Clarivate Analytics), Cochrane Database of Systematic Reviews (Wiley), DARE (Centre for Reviews & Dissemination), Health Technology Assessment (Centre for Reviews & Dissemination), NHS EED (Centre for Reviews & Dissemination), and Epistemonikos

Medline (EBSCO)

((MH "Aged+") OR (MH "Health Services for the Aged") OR (MH "Geriatric Assessment") OR (MH "Geriatrics") OR (MH "Geriatric Psychiatry") OR (MH "Geriatric Nursing") OR (MH "Geriatric Dentistry") OR (MH "Dental Care for Aged") OR (MH "Homes for the Aged") OR (MH "Nursing Homes+") OR (MH "Housing for the Elderly") OR TI (elder OR elders OR eldest OR elderly OR geriatric* OR “old age” OR “oldest old” OR “senior citizen” OR “senior citizens” OR “very old” OR septuagenarian* OR octogenarian* OR octagenarian* OR nonagenarian* OR centarian* OR centenarian* OR supercentenarian* OR “older people” OR “older person” OR “older subject” OR “older subjects” OR “older patient” OR “older patients” OR “older age” OR “older aged” OR “older ages” OR “older adult” OR “older adults” OR “older man” OR “older men” OR “older male” OR “older males” OR “older woman” OR “older women” OR “older female” OR “older females” OR “older veterans” OR “older population”) OR AB (elder OR elders OR eldest OR elderly OR geriatric* OR “old age” OR “oldest old” OR “senior citizen” OR “senior citizens” OR “very old” OR septuagenarian* OR octogenarian* OR octagenarian* OR nonagenarian* OR centarian* OR centenarian* OR supercentenarian* OR “older people” OR “older person” OR “older subject” OR “older subjects” OR “older patient” OR “older patients” OR “older age” OR “older aged” OR “older ages” OR “older adult” OR “older adults” OR “older man” OR “older men” OR “older male” OR “older males” OR “older woman” OR “older women” OR “older female” OR “older females” OR “older veterans” OR “older population”))

AND

(MH (“Inappropriate Prescribing”) OR MM (“Drug Utilization Review”) OR TI (deprescri* OR “de prescribing” OR “de prescription” OR “inappropriate prescribing” OR “inappropriate prescription” OR “inappropriate prescriptions” OR “over prescribing” OR “inappropriate medications” OR “inappropriate medication”) OR AB (deprescri* OR “de prescribing” OR “de prescription” OR “inappropriate prescribing” OR “inappropriate prescription” OR “inappropriate prescriptions” OR “over prescribing” OR “inappropriate medications” OR “inappropriate medication”))

OR

((MH "Aged+") OR (MH "Health Services for the Aged") OR (MH "Geriatric Assessment") OR (MH "Geriatrics") OR (MH "Geriatric Psychiatry") OR (MH "Geriatric Nursing") OR (MH "Geriatric Dentistry") OR (MH "Dental Care for Aged") OR (MH "Homes for the Aged") OR (MH "Nursing Homes+") OR (MH "Housing for the Elderly") OR TI (elder OR elders OR eldest OR elderly OR geriatric* OR “old age” OR “oldest old” OR “senior citizen” OR “senior citizens” OR “very old” OR septuagenarian* OR octogenarian* OR octagenarian* OR nonagenarian* OR centarian* OR centenarian* OR supercentenarian* OR “older people” OR “older person” OR “older subject” OR “older subjects” OR “older patient” OR “older patients” OR “older age” OR “older aged” OR “older ages” OR “older adult” OR “older adults” OR “older man” OR “older men” OR “older male” OR “older males” OR “older woman” OR “older women” OR “older female” OR “older females” OR “older veterans” OR “older population”) OR AB (elder OR elders OR eldest OR elderly OR geriatric* OR “old age” OR “oldest old” OR “senior citizen” OR “senior citizens” OR “very old” OR septuagenarian* OR octogenarian* OR octagenarian* OR nonagenarian* OR centarian* OR centenarian* OR supercentenarian* OR “older people” OR “older person” OR “older subject” OR “older subjects” OR “older patient” OR “older patients” OR “older age” OR “older aged” OR “older ages” OR “older adult” OR “older adults” OR “older man” OR “older men” OR “older male” OR “older males” OR “older woman” OR “older women” OR “older female” OR “older females” OR “older veterans” OR “older population”))

AND

(TI ((medication* OR medicin* OR prescription* OR prescrib* OR polypharmacy* OR pharmaceutical* OR drug OR drugs OR aperient OR fiber OR laxative* OR lactulose OR glycerol OR sorbitol OR macrogol OR docusate OR bisacodyl OR senna OR picosulfate OR paraffin OR poloxamer OR “hormone replacement therapy” OR *estrogen OR *estradiol OR *estriol OR tibolone OR “direct thrombin inhibitor” OR bivalirudin OR dabigatran OR warfarin OR anticoagula* OR “factor xa inhibitor” OR “factor xa inhibitors” OR apixaban OR fondaparinux OR rivaroxaban OR pyridoxine OR thiamine OR calcitriol OR cholecalciferol OR colecalciferol OR ergocalciferol OR micronutrient OR vitamin OR mineral OR iron OR ferrous OR calcium OR potassium OR magnesium OR ascorb* OR folic OR folate OR hydroxocobalamin OR cyanocobalamin OR “proton pump inhibitor” OR PPI OR “acid suppression” OR pantoprazole OR omeprazole OR esomeprazole OR lansoprazole OR rabeprazole OR antacid OR cimetidine OR famotidine OR nizatidine OR ranitidine OR fenofibrate OR gemfibrozil OR fibrate OR ezetimibe OR corticosteroid OR glucocorticoid OR steroid OR dihydropyridines OR amlodipine OR diltiazem OR felodipine OR lercanidipine OR nifedipine OR nimodipine OR verapamil OR nitrate OR nitrates OR trinitrate OR isosorbide OR mononitrate OR ivabradine OR nicorandil OR perhexiline OR antipsychotic OR amisulpride OR aripiprazole OR asenapine OR chlorpromazine OR clozapine OR droperidol OR flupenthixol OR fluphenazine OR haloperidol OR olanzapine OR paliperidone OR pericyazine OR quetiapine OR risperidone OR trifluoperazine OR ziprasidone OR zuclopenthixol OR lithium OR buspirone OR diphenhydramine OR doxylamine OR melatonin OR zolpidem OR zopiclone OR benzodiazepine OR alprazolam OR bromazepam OR midazolam OR clobazam OR nitrazepam OR oxazepam OR temazepam OR triazolam OR clonazepam OR diazepam OR flunitrazepam OR lorazepam OR hypnotic OR sedative OR anticholinergic OR darifenacin OR oxybutynin OR tolterodine OR benzhexol OR glycopyrronium OR hyoscine OR bromide OR biperiden OR orphenadrine OR solifenacin OR dopamine OR bromocriptine OR cabergoline OR pergolide OR apomorphine OR pramipexole OR rotigotine OR rasagiline OR selegiline OR amantadine OR entacapone OR levodopa OR anticholinesterase OR donepezil OR memantine OR galantamine OR rivastigmine OR bisphosphonate OR raloxifene OR alendronate OR clodronate OR ibandron* OR pamidronate OR risedronate OR tiludronate OR coledron* OR strontium OR aspirin OR antiplatelet OR clopidogrel OR thienopyridines OR prasugrel OR ticlopidine OR dipyridamole OR celecoxib OR diclofenac OR etoricoxib OR ibuprofen OR indomethacin OR ketoprofen OR ketorolac OR meloxicam OR naproxen OR piroxicam OR NSAID OR “non steroidal anti inflammatory” OR colchicine OR allopurinol OR quinine OR levothyroxine OR thyroxine OR liothyronine OR carbimazole OR propylthiouracil OR iodine OR paracetamol OR acetaminophen OR antiglycaemi* OR biguanide OR metformin OR sulphonylurea OR gliclazide OR glibenclamide OR glimepiride OR glipizide OR pioglitazone OR rosiglitazone OR insulin OR acarbose OR alogliptin OR linagliptin OR saxagliptin OR sitagliptin OR vildagliptin OR antiepilep* OR anticonvulsant OR valprotate OR carbamazepine OR barbiturate OR lithium OR lamotrigine OR gabapentin OR phenytoin OR pregabalin OR ethosuximide OR levetiracetam OR topiramate OR vigabatrin OR tiagabine OR antihistamine OR cyclizine OR cyproheptadine OR dexchlorpheniramine OR diphenhydramine OR doxylamine OR pheniramine OR promethazine OR trimeprazine OR cetirizine OR desloratadine OR fexofenadine OR levocetirizine OR loratadine OR diuretic OR thiazide OR frusemide OR indapamide OR hydrochlorothiazide OR spironolactone OR digoxin OR amiodarone OR antiarrhythmi* OR antidepressant* OR SSRI OR “serotonin reuptake inhibitor” OR mirtazapine OR TCA OR “tricyclic antidepressant” OR mianserin OR SNRI OR “serotonin and noradrenaline reuptake inhibitor” OR venlafaxine OR duloxetine OR desvenlafaxine OR “beta blocker” OR betablocker OR “alpha blocker” OR “angiotensin ii receptor antagonist” OR sartan OR “angiotensin converting enzyme inhibitors” OR acei OR statin OR “hmg coa reductase inhibitor”) N3 (deprescri* OR withdraw* OR withdrew OR ceas* OR cessation OR withheld OR withhold OR discontinu* OR reduc* OR taper* OR stop OR stopping OR stopped OR end OR ending OR ended OR remove* OR removal OR removing)) OR AB ((medication* OR medicin* OR prescription* OR prescrib* OR polypharmacy* OR pharmaceutical* OR drug OR drugs OR aperient OR fiber OR laxative* OR lactulose OR glycerol OR sorbitol OR macrogol OR docusate OR bisacodyl OR senna OR picosulfate OR paraffin OR poloxamer OR “hormone replacement therapy” OR *estrogen OR *estradiol OR *estriol OR tibolone OR “direct thrombin inhibitor” OR bivalirudin OR dabigatran OR warfarin OR anticoagula* OR “factor xa inhibitor” OR “factor xa inhibitors” OR apixaban OR fondaparinux OR rivaroxaban OR pyridoxine OR thiamine OR calcitriol OR cholecalciferol OR colecalciferol OR ergocalciferol OR micronutrient OR vitamin OR mineral OR iron OR ferrous OR calcium OR potassium OR magnesium OR ascorb* OR folic OR folate OR hydroxocobalamin OR cyanocobalamin OR “proton pump inhibitor” OR PPI OR “acid suppression” OR pantoprazole OR omeprazole OR esomeprazole OR lansoprazole OR rabeprazole OR antacid OR cimetidine OR famotidine OR nizatidine OR ranitidine OR fenofibrate OR gemfibrozil OR fibrate OR ezetimibe OR corticosteroid OR glucocorticoid OR steroid OR dihydropyridines OR amlodipine OR diltiazem OR felodipine OR lercanidipine OR nifedipine OR nimodipine OR verapamil OR nitrate OR nitrates OR trinitrate OR isosorbide OR mononitrate OR ivabradine OR nicorandil OR perhexiline OR antipsychotic OR amisulpride OR aripiprazole OR asenapine OR chlorpromazine OR clozapine OR droperidol OR flupenthixol OR fluphenazine OR haloperidol OR olanzapine OR paliperidone OR pericyazine OR quetiapine OR risperidone OR trifluoperazine OR ziprasidone OR zuclopenthixol OR lithium OR buspirone OR diphenhydramine OR doxylamine OR melatonin OR zolpidem OR zopiclone OR benzodiazepine OR alprazolam OR bromazepam OR midazolam OR clobazam OR nitrazepam OR oxazepam OR temazepam OR triazolam OR clonazepam OR diazepam OR flunitrazepam OR lorazepam OR hypnotic OR sedative OR anticholinergic OR darifenacin OR oxybutynin OR tolterodine OR benzhexol OR glycopyrronium OR hyoscine OR bromide OR biperiden OR orphenadrine OR solifenacin OR dopamine OR bromocriptine OR cabergoline OR pergolide OR apomorphine OR pramipexole OR rotigotine OR rasagiline OR selegiline OR amantadine OR entacapone OR levodopa OR anticholinesterase OR donepezil OR memantine OR galantamine OR rivastigmine OR bisphosphonate OR raloxifene OR alendronate OR clodronate OR ibandron* OR pamidronate OR risedronate OR tiludronate OR coledron* OR strontium OR aspirin OR antiplatelet OR clopidogrel OR thienopyridines OR prasugrel OR ticlopidine OR dipyridamole OR celecoxib OR diclofenac OR etoricoxib OR ibuprofen OR indomethacin OR ketoprofen OR ketorolac OR meloxicam OR naproxen OR piroxicam OR NSAID OR “non steroidal anti inflammatory” OR colchicine OR allopurinol OR quinine OR levothyroxine OR thyroxine OR liothyronine OR carbimazole OR propylthiouracil OR iodine OR paracetamol OR acetaminophen OR antiglycaemi* OR biguanide OR metformin OR sulphonylurea OR gliclazide OR glibenclamide OR glimepiride OR glipizide OR pioglitazone OR rosiglitazone OR insulin OR acarbose OR alogliptin OR linagliptin OR saxagliptin OR sitagliptin OR vildagliptin OR antiepilep* OR anticonvulsant OR valprotate OR carbamazepine OR barbiturate OR lithium OR lamotrigine OR gabapentin OR phenytoin OR pregabalin OR ethosuximide OR levetiracetam OR topiramate OR vigabatrin OR tiagabine OR antihistamine OR cyclizine OR cyproheptadine OR dexchlorpheniramine OR diphenhydramine OR doxylamine OR pheniramine OR promethazine OR trimeprazine OR cetirizine OR desloratadine OR fexofenadine OR levocetirizine OR loratadine OR diuretic OR thiazide OR frusemide OR indapamide OR hydrochlorothiazide OR spironolactone OR digoxin OR amiodarone OR antiarrhythmi* OR antidepressant* OR SSRI OR “serotonin reuptake inhibitor” OR mirtazapine OR TCA OR “tricyclic antidepressant” OR mianserin OR SNRI OR “serotonin and noradrenaline reuptake inhibitor” OR venlafaxine OR duloxetine OR desvenlafaxine OR “beta blocker” OR betablocker OR “alpha blocker” OR “angiotensin ii receptor antagonist” OR sartan OR “angiotensin converting enzyme inhibitors” OR acei OR statin OR “hmg coa reductase inhibitor”) N3 (deprescri* OR withdraw* OR withdrew OR ceas* OR cessation OR withheld OR withhold OR discontinu* OR reduc* OR taper* OR stop OR stopping OR stopped OR end OR ending OR ended OR remove* OR removal OR removing)))

AND

(MH "Systematic Review") OR (MH "Systematic Reviews as Topic") OR (MH "Meta-Analysis") OR (MH "Network Meta-Analysis") OR (MH "Meta-Analysis as Topic+") OR TI ("systematic review" OR "systematic literature review" OR “meta analysis” OR “metaanalysis”) OR AB ("systematic review" OR "systematic literature review" OR “meta analysis” OR “metaanalysis”)

Embase

('aged'/exp OR 'elderly care'/exp OR 'geriatric assessment'/exp OR 'geriatrics'/exp OR 'elderly care'/exp OR 'nursing home'/exp OR (elder OR elders OR eldest OR elderly OR geriatric* OR “old age” OR “oldest old” OR “senior citizen” OR “senior citizens” OR “very old” OR septuagenarian* OR octogenarian* OR octagenarian* OR nonagenarian* OR centarian* OR centenarian* OR supercentenarian* OR “older people” OR “older person” OR “older subject” OR “older subjects” OR “older patient” OR “older patients” OR “older age” OR “older aged” OR “older ages” OR “older adult” OR “older adults” OR “older man” OR “older men” OR “older male” OR “older males” OR “older woman” OR “older women” OR “older female” OR “older females” OR “older veterans” OR “older population”):ti,ab)

AND

('inappropriate prescribing'/exp OR 'drug utilization review'/mj OR (deprescri* OR “de prescribing” OR “de prescription” OR “inappropriate prescribing” OR “inappropriate prescription” OR “inappropriate prescriptions” OR “over prescribing” OR “inappropriate medications” OR “inappropriate medication”):ti,ab

OR

((medication* OR medicin* OR prescription* OR prescrib* OR polypharmacy* OR pharmaceutical* OR drug OR drugs OR aperient OR fiber OR laxative* OR lactulose OR glycerol OR sorbitol OR macrogol OR docusate OR bisacodyl OR senna OR picosulfate OR paraffin OR poloxamer OR “hormone replacement therapy” OR estrogen OR estradiol OR estriol OR tibolone OR “direct thrombin inhibitor” OR bivalirudin OR dabigatran OR warfarin OR anticoagula* OR “factor xa inhibitor” OR “factor xa inhibitors” OR apixaban OR fondaparinux OR rivaroxaban OR pyridoxine OR thiamine OR calcitriol OR cholecalciferol OR colecalciferol OR ergocalciferol OR micronutrient OR vitamin OR mineral OR iron OR ferrous OR calcium OR potassium OR magnesium OR ascorb* OR folic OR folate OR hydroxocobalamin OR cyanocobalamin OR “proton pump inhibitor” OR PPI OR “acid suppression” OR pantoprazole OR omeprazole OR esomeprazole OR lansoprazole OR rabeprazole OR antacid OR cimetidine OR famotidine OR nizatidine OR ranitidine OR fenofibrate OR gemfibrozil OR fibrate OR ezetimibe OR corticosteroid OR glucocorticoid OR steroid OR dihydropyridines OR amlodipine OR diltiazem OR felodipine OR lercanidipine OR nifedipine OR nimodipine OR verapamil OR nitrate OR nitrates OR trinitrate OR isosorbide OR mononitrate OR ivabradine OR nicorandil OR perhexiline OR antipsychotic OR amisulpride OR aripiprazole OR asenapine OR chlorpromazine OR clozapine OR droperidol OR flupenthixol OR fluphenazine OR haloperidol OR olanzapine OR paliperidone OR pericyazine OR quetiapine OR risperidone OR trifluoperazine OR ziprasidone OR zuclopenthixol OR lithium OR buspirone OR diphenhydramine OR doxylamine OR melatonin OR zolpidem OR zopiclone OR benzodiazepine OR alprazolam OR bromazepam OR midazolam OR clobazam OR nitrazepam OR oxazepam OR temazepam OR triazolam OR clonazepam OR diazepam OR flunitrazepam OR lorazepam OR hypnotic OR sedative OR anticholinergic OR darifenacin OR oxybutynin OR tolterodine OR benzhexol OR glycopyrronium OR hyoscine OR bromide OR biperiden OR orphenadrine OR solifenacin OR dopamine OR bromocriptine OR cabergoline OR pergolide OR apomorphine OR pramipexole OR rotigotine OR rasagiline OR selegiline OR amantadine OR entacapone OR levodopa OR anticholinesterase OR donepezil OR memantine OR galantamine OR rivastigmine OR bisphosphonate OR raloxifene OR alendronate OR clodronate OR ibandron* OR pamidronate OR risedronate OR tiludronate OR coledron* OR strontium OR aspirin OR antiplatelet OR clopidogrel OR thienopyridines OR prasugrel OR ticlopidine OR dipyridamole OR celecoxib OR diclofenac OR etoricoxib OR ibuprofen OR indomethacin OR ketoprofen OR ketorolac OR meloxicam OR naproxen OR piroxicam OR NSAID OR “non steroidal anti inflammatory” OR colchicine OR allopurinol OR quinine OR levothyroxine OR thyroxine OR liothyronine OR carbimazole OR propylthiouracil OR iodine OR paracetamol OR acetaminophen OR antiglycaemi* OR biguanide OR metformin OR sulphonylurea OR gliclazide OR glibenclamide OR glimepiride OR glipizide OR pioglitazone OR rosiglitazone OR insulin OR acarbose OR alogliptin OR linagliptin OR saxagliptin OR sitagliptin OR vildagliptin OR antiepilep* OR anticonvulsant OR valprotate OR carbamazepine OR barbiturate OR lithium OR lamotrigine OR gabapentin OR phenytoin OR pregabalin OR ethosuximide OR levetiracetam OR topiramate OR vigabatrin OR tiagabine OR antihistamine OR cyclizine OR cyproheptadine OR dexchlorpheniramine OR diphenhydramine OR doxylamine OR pheniramine OR promethazine OR trimeprazine OR cetirizine OR desloratadine OR fexofenadine OR levocetirizine OR loratadine OR diuretic OR thiazide OR frusemide OR indapamide OR hydrochlorothiazide OR spironolactone OR digoxin OR amiodarone OR antiarrhythmi* OR antidepressant* OR SSRI OR “serotonin reuptake inhibitor” OR mirtazapine OR TCA OR “tricyclic antidepressant” OR mianserin OR SNRI OR “serotonin and noradrenaline reuptake inhibitor” OR venlafaxine OR duloxetine OR desvenlafaxine OR “beta blocker” OR betablocker OR “alpha blocker” OR “angiotensin ii receptor antagonist” OR sartan OR “angiotensin converting enzyme inhibitors” OR acei OR statin OR “hmg coa reductase inhibitor”) NEAR/3 (deprescri* OR withdraw* OR withdrew OR ceas* OR cessation OR withheld OR withhold OR discontinu* OR reduc* OR taper* OR stop OR stopping OR stopped OR end OR ending OR ended OR remove* OR removal OR removing)):ti,ab)

AND

('systematic review'/exp OR 'systematic review (topic)'/exp OR 'meta analysis'/exp OR 'meta analysis (topic)'/exp OR 'systematic review':ti,ab OR 'systematic literature review':ti,ab OR 'meta analysis':ti,ab OR metaanalysis:ti,ab)

CINAHL Complete (EBSCO)

((MH "Aged+") OR (MH "Health Services for the Aged") OR (MH "Housing for the Elderly") OR (MH "Gerontologic Care") OR (MH "Gerontologic Nursing+") OR (MH "Rehabilitation, Geriatric") OR (MH "Geriatric Psychiatry") OR (MH "Geriatric Nutrition") OR (MH "Geriatric Assessment+") OR (MH "Gerontologic Nurse Practitioners") OR (MH "Dental Care for Aged") OR TI (elder OR elders OR eldest OR elderly OR geriatric* OR “old age” OR “oldest old” OR “senior citizen” OR “senior citizens” OR “very old” OR septuagenarian* OR octogenarian* OR octagenarian* OR nonagenarian* OR centarian* OR centenarian* OR supercentenarian* OR “older people” OR “older person” OR “older subject” OR “older subjects” OR “older patient” OR “older patients” OR “older age” OR “older aged” OR “older ages” OR “older adult” OR “older adults” OR “older man” OR “older men” OR “older male” OR “older males” OR “older woman” OR “older women” OR “older female” OR “older females” OR “older veterans” OR “older population”) OR AB (elder OR elders OR eldest OR elderly OR geriatric* OR “old age” OR “oldest old” OR “senior citizen” OR “senior citizens” OR “very old” OR septuagenarian* OR octogenarian* OR octagenarian* OR nonagenarian* OR centarian* OR centenarian* OR supercentenarian* OR “older people” OR “older person” OR “older subject” OR “older subjects” OR “older patient” OR “older patients” OR “older age” OR “older aged” OR “older ages” OR “older adult” OR “older adults” OR “older man” OR “older men” OR “older male” OR “older males” OR “older woman” OR “older women” OR “older female” OR “older females” OR “older veterans” OR “older population”))

AND

((MH "Inappropriate Prescribing") OR (MM "Drug Utilization") OR TI (deprescri* OR “de prescribing” OR “de prescription” OR “inappropriate prescribing” OR “inappropriate prescription” OR “inappropriate prescriptions” OR “over prescribing” OR “inappropriate medications” OR “inappropriate medication”) OR AB (deprescri* OR “de prescribing” OR “de prescription” OR “inappropriate prescribing” OR “inappropriate prescription” OR “inappropriate prescriptions” OR “over prescribing” OR “inappropriate medications” OR “inappropriate medication”)

OR

TI ((medication* OR medicin* OR prescription* OR prescrib* OR polypharmacy* OR pharmaceutical* OR drug OR drugs OR aperient OR fiber OR laxative* OR lactulose OR glycerol OR sorbitol OR macrogol OR docusate OR bisacodyl OR senna OR picosulfate OR paraffin OR poloxamer OR “hormone replacement therapy” OR *estrogen OR *estradiol OR *estriol OR tibolone OR “direct thrombin inhibitor” OR bivalirudin OR dabigatran OR warfarin OR anticoagula* OR “factor xa inhibitor” OR “factor xa inhibitors” OR apixaban OR fondaparinux OR rivaroxaban OR pyridoxine OR thiamine OR calcitriol OR cholecalciferol OR colecalciferol OR ergocalciferol OR micronutrient OR vitamin OR mineral OR iron OR ferrous OR calcium OR potassium OR magnesium OR ascorb* OR folic OR folate OR hydroxocobalamin OR cyanocobalamin OR “proton pump inhibitor” OR PPI OR “acid suppression” OR pantoprazole OR omeprazole OR esomeprazole OR lansoprazole OR rabeprazole OR antacid OR cimetidine OR famotidine OR nizatidine OR ranitidine OR fenofibrate OR gemfibrozil OR fibrate OR ezetimibe OR corticosteroid OR glucocorticoid OR steroid OR dihydropyridines OR amlodipine OR diltiazem OR felodipine OR lercanidipine OR nifedipine OR nimodipine OR verapamil OR nitrate OR nitrates OR trinitrate OR isosorbide OR mononitrate OR ivabradine OR nicorandil OR perhexiline OR antipsychotic OR amisulpride OR aripiprazole OR asenapine OR chlorpromazine OR clozapine OR droperidol OR flupenthixol OR fluphenazine OR haloperidol OR olanzapine OR paliperidone OR pericyazine OR quetiapine OR risperidone OR trifluoperazine OR ziprasidone OR zuclopenthixol OR lithium OR buspirone OR diphenhydramine OR doxylamine OR melatonin OR zolpidem OR zopiclone OR benzodiazepine OR alprazolam OR bromazepam OR midazolam OR clobazam OR nitrazepam OR oxazepam OR temazepam OR triazolam OR clonazepam OR diazepam OR flunitrazepam OR lorazepam OR hypnotic OR sedative OR anticholinergic OR darifenacin OR oxybutynin OR tolterodine OR benzhexol OR glycopyrronium OR hyoscine OR bromide OR biperiden OR orphenadrine OR solifenacin OR dopamine OR bromocriptine OR cabergoline OR pergolide OR apomorphine OR pramipexole OR rotigotine OR rasagiline OR selegiline OR amantadine OR entacapone OR levodopa OR anticholinesterase OR donepezil OR memantine OR galantamine OR rivastigmine OR bisphosphonate OR raloxifene OR alendronate OR clodronate OR ibandron* OR pamidronate OR risedronate OR tiludronate OR coledron* OR strontium OR aspirin OR antiplatelet OR clopidogrel OR thienopyridines OR prasugrel OR ticlopidine OR dipyridamole OR celecoxib OR diclofenac OR etoricoxib OR ibuprofen OR indomethacin OR ketoprofen OR ketorolac OR meloxicam OR naproxen OR piroxicam OR NSAID OR “non steroidal anti inflammatory” OR colchicine OR allopurinol OR quinine OR levothyroxine OR thyroxine OR liothyronine OR carbimazole OR propylthiouracil OR iodine OR paracetamol OR acetaminophen OR antiglycaemi* OR biguanide OR metformin OR sulphonylurea OR gliclazide OR glibenclamide OR glimepiride OR glipizide OR pioglitazone OR rosiglitazone OR insulin OR acarbose OR alogliptin OR linagliptin OR saxagliptin OR sitagliptin OR vildagliptin OR antiepilep* OR anticonvulsant OR valprotate OR carbamazepine OR barbiturate OR lithium OR lamotrigine OR gabapentin OR phenytoin OR pregabalin OR ethosuximide OR levetiracetam OR topiramate OR vigabatrin OR tiagabine OR antihistamine OR cyclizine OR cyproheptadine OR dexchlorpheniramine OR diphenhydramine OR doxylamine OR pheniramine OR promethazine OR trimeprazine OR cetirizine OR desloratadine OR fexofenadine OR levocetirizine OR loratadine OR diuretic OR thiazide OR frusemide OR indapamide OR hydrochlorothiazide OR spironolactone OR digoxin OR amiodarone OR antiarrhythmi* OR antidepressant* OR SSRI OR “serotonin reuptake inhibitor” OR mirtazapine OR TCA OR “tricyclic antidepressant” OR mianserin OR SNRI OR “serotonin and noradrenaline reuptake inhibitor” OR venlafaxine OR duloxetine OR desvenlafaxine OR “beta blocker” OR betablocker OR “alpha blocker” OR “angiotensin ii receptor antagonist” OR sartan OR “angiotensin converting enzyme inhibitors” OR acei OR statin OR “hmg coa reductase inhibitor”) N3 (deprescri* OR withdraw* OR withdrew OR ceas* OR cessation OR withheld OR withhold OR discontinu* OR reduc* OR taper* OR stop OR stopping OR stopped OR end OR ending OR ended OR remove* OR removal OR removing)) OR AB ((medication* OR medicin* OR prescription* OR prescrib* OR polypharmacy* OR pharmaceutical* OR drug OR drugs OR aperient OR fiber OR laxative* OR lactulose OR glycerol OR sorbitol OR macrogol OR docusate OR bisacodyl OR senna OR picosulfate OR paraffin OR poloxamer OR “hormone replacement therapy” OR *estrogen OR *estradiol OR *estriol OR tibolone OR “direct thrombin inhibitor” OR bivalirudin OR dabigatran OR warfarin OR anticoagula* OR “factor xa inhibitor” OR “factor xa inhibitors” OR apixaban OR fondaparinux OR rivaroxaban OR pyridoxine OR thiamine OR calcitriol OR cholecalciferol OR colecalciferol OR ergocalciferol OR micronutrient OR vitamin OR mineral OR iron OR ferrous OR calcium OR potassium OR magnesium OR ascorb* OR folic OR folate OR hydroxocobalamin OR cyanocobalamin OR “proton pump inhibitor” OR PPI OR “acid suppression” OR pantoprazole OR omeprazole OR esomeprazole OR lansoprazole OR rabeprazole OR antacid OR cimetidine OR famotidine OR nizatidine OR ranitidine OR fenofibrate OR gemfibrozil OR fibrate OR ezetimibe OR corticosteroid OR glucocorticoid OR steroid OR dihydropyridines OR amlodipine OR diltiazem OR felodipine OR lercanidipine OR nifedipine OR nimodipine OR verapamil OR nitrate OR nitrates OR trinitrate OR isosorbide OR mononitrate OR ivabradine OR nicorandil OR perhexiline OR antipsychotic OR amisulpride OR aripiprazole OR asenapine OR chlorpromazine OR clozapine OR droperidol OR flupenthixol OR fluphenazine OR haloperidol OR olanzapine OR paliperidone OR pericyazine OR quetiapine OR risperidone OR trifluoperazine OR ziprasidone OR zuclopenthixol OR lithium OR buspirone OR diphenhydramine OR doxylamine OR melatonin OR zolpidem OR zopiclone OR benzodiazepine OR alprazolam OR bromazepam OR midazolam OR clobazam OR nitrazepam OR oxazepam OR temazepam OR triazolam OR clonazepam OR diazepam OR flunitrazepam OR lorazepam OR hypnotic OR sedative OR anticholinergic OR darifenacin OR oxybutynin OR tolterodine OR benzhexol OR glycopyrronium OR hyoscine OR bromide OR biperiden OR orphenadrine OR solifenacin OR dopamine OR bromocriptine OR cabergoline OR pergolide OR apomorphine OR pramipexole OR rotigotine OR rasagiline OR selegiline OR amantadine OR entacapone OR levodopa OR anticholinesterase OR donepezil OR memantine OR galantamine OR rivastigmine OR bisphosphonate OR raloxifene OR alendronate OR clodronate OR ibandron* OR pamidronate OR risedronate OR tiludronate OR coledron* OR strontium OR aspirin OR antiplatelet OR clopidogrel OR thienopyridines OR prasugrel OR ticlopidine OR dipyridamole OR celecoxib OR diclofenac OR etoricoxib OR ibuprofen OR indomethacin OR ketoprofen OR ketorolac OR meloxicam OR naproxen OR piroxicam OR NSAID OR “non steroidal anti inflammatory” OR colchicine OR allopurinol OR quinine OR levothyroxine OR thyroxine OR liothyronine OR carbimazole OR propylthiouracil OR iodine OR paracetamol OR acetaminophen OR antiglycaemi* OR biguanide OR metformin OR sulphonylurea OR gliclazide OR glibenclamide OR glimepiride OR glipizide OR pioglitazone OR rosiglitazone OR insulin OR acarbose OR alogliptin OR linagliptin OR saxagliptin OR sitagliptin OR vildagliptin OR antiepilep* OR anticonvulsant OR valprotate OR carbamazepine OR barbiturate OR lithium OR lamotrigine OR gabapentin OR phenytoin OR pregabalin OR ethosuximide OR levetiracetam OR topiramate OR vigabatrin OR tiagabine OR antihistamine OR cyclizine OR cyproheptadine OR dexchlorpheniramine OR diphenhydramine OR doxylamine OR pheniramine OR promethazine OR trimeprazine OR cetirizine OR desloratadine OR fexofenadine OR levocetirizine OR loratadine OR diuretic OR thiazide OR frusemide OR indapamide OR hydrochlorothiazide OR spironolactone OR digoxin OR amiodarone OR antiarrhythmi* OR antidepressant* OR SSRI OR “serotonin reuptake inhibitor” OR mirtazapine OR TCA OR “tricyclic antidepressant” OR mianserin OR SNRI OR “serotonin and noradrenaline reuptake inhibitor” OR venlafaxine OR duloxetine OR desvenlafaxine OR “beta blocker” OR betablocker OR “alpha blocker” OR “angiotensin ii receptor antagonist” OR sartan OR “angiotensin converting enzyme inhibitors” OR acei OR statin OR “hmg coa reductase inhibitor”) N3 (deprescri* OR withdraw* OR withdrew OR ceas* OR cessation OR withheld OR withhold OR discontinu* OR reduc* OR taper* OR stop OR stopping OR stopped OR end OR ending OR ended OR remove* OR removal OR removing)))

AND

(TI ("systematic review" OR "systematic literature review" OR “meta analysis” OR “metaanalysis”) OR AB ("systematic review" OR "systematic literature review" OR “meta analysis” OR “metaanalysis”))

APA PsycInfo (EBSCO)

(DE "Geriatric Patients" OR DE "Geriatrics" OR DE "Gerontology" OR DE "Geriatric Assessment" OR DE "Aging" OR DE "Geriatric Psychiatry" OR DE "Geriatric Psychotherapy" OR TI (elder OR elders OR eldest OR elderly OR geriatric* OR “old age” OR “oldest old” OR “senior citizen” OR “senior citizens” OR “very old” OR septuagenarian* OR octogenarian* OR octagenarian* OR nonagenarian* OR centarian* OR centenarian* OR supercentenarian* OR “older people” OR “older person” OR “older subject” OR “older subjects” OR “older patient” OR “older patients” OR “older age” OR “older aged” OR “older ages” OR “older adult” OR “older adults” OR “older man” OR “older men” OR “older male” OR “older males” OR “older woman” OR “older women” OR “older female” OR “older females” OR “older veterans” OR “older population”) OR AB (elder OR elders OR eldest OR elderly OR geriatric* OR “old age” OR “oldest old” OR “senior citizen” OR “senior citizens” OR “very old” OR septuagenarian* OR octogenarian* OR octagenarian* OR nonagenarian* OR centarian* OR centenarian* OR supercentenarian* OR “older people” OR “older person” OR “older subject” OR “older subjects” OR “older patient” OR “older patients” OR “older age” OR “older aged” OR “older ages” OR “older adult” OR “older adults” OR “older man” OR “older men” OR “older male” OR “older males” OR “older woman” OR “older women” OR “older female” OR “older females” OR “older veterans” OR “older population”))

AND

(TI (deprescri* OR “de prescribing” OR “de prescription” OR “inappropriate prescribing” OR “inappropriate prescription” OR “inappropriate prescriptions” OR “over prescribing” OR “inappropriate medications” OR “inappropriate medication”) OR AB (deprescri* OR “de prescribing” OR “de prescription” OR “inappropriate prescribing” OR “inappropriate prescription” OR “inappropriate prescriptions” OR “over prescribing” OR “inappropriate medications” OR “inappropriate medication”)

OR

TI ((medication* OR medicin* OR prescription* OR prescrib* OR polypharmacy* OR pharmaceutical* OR drug OR drugs OR aperient OR fiber OR laxative* OR lactulose OR glycerol OR sorbitol OR macrogol OR docusate OR bisacodyl OR senna OR picosulfate OR paraffin OR poloxamer OR “hormone replacement therapy” OR *estrogen OR *estradiol OR *estriol OR tibolone OR “direct thrombin inhibitor” OR bivalirudin OR dabigatran OR warfarin OR anticoagula* OR “factor xa inhibitor” OR “factor xa inhibitors” OR apixaban OR fondaparinux OR rivaroxaban OR pyridoxine OR thiamine OR calcitriol OR cholecalciferol OR colecalciferol OR ergocalciferol OR micronutrient OR vitamin OR mineral OR iron OR ferrous OR calcium OR potassium OR magnesium OR ascorb* OR folic OR folate OR hydroxocobalamin OR cyanocobalamin OR “proton pump inhibitor” OR PPI OR “acid suppression” OR pantoprazole OR omeprazole OR esomeprazole OR lansoprazole OR rabeprazole OR antacid OR cimetidine OR famotidine OR nizatidine OR ranitidine OR fenofibrate OR gemfibrozil OR fibrate OR ezetimibe OR corticosteroid OR glucocorticoid OR steroid OR dihydropyridines OR amlodipine OR diltiazem OR felodipine OR lercanidipine OR nifedipine OR nimodipine OR verapamil OR nitrate OR nitrates OR trinitrate OR isosorbide OR mononitrate OR ivabradine OR nicorandil OR perhexiline OR antipsychotic OR amisulpride OR aripiprazole OR asenapine OR chlorpromazine OR clozapine OR droperidol OR flupenthixol OR fluphenazine OR haloperidol OR olanzapine OR paliperidone OR pericyazine OR quetiapine OR risperidone OR trifluoperazine OR ziprasidone OR zuclopenthixol OR lithium OR buspirone OR diphenhydramine OR doxylamine OR melatonin OR zolpidem OR zopiclone OR benzodiazepine OR alprazolam OR bromazepam OR midazolam OR clobazam OR nitrazepam OR oxazepam OR temazepam OR triazolam OR clonazepam OR diazepam OR flunitrazepam OR lorazepam OR hypnotic OR sedative OR anticholinergic OR darifenacin OR oxybutynin OR tolterodine OR benzhexol OR glycopyrronium OR hyoscine OR bromide OR biperiden OR orphenadrine OR solifenacin OR dopamine OR bromocriptine OR cabergoline OR pergolide OR apomorphine OR pramipexole OR rotigotine OR rasagiline OR selegiline OR amantadine OR entacapone OR levodopa OR anticholinesterase OR donepezil OR memantine OR galantamine OR rivastigmine OR bisphosphonate OR raloxifene OR alendronate OR clodronate OR ibandron* OR pamidronate OR risedronate OR tiludronate OR coledron* OR strontium OR aspirin OR antiplatelet OR clopidogrel OR thienopyridines OR prasugrel OR ticlopidine OR dipyridamole OR celecoxib OR diclofenac OR etoricoxib OR ibuprofen OR indomethacin OR ketoprofen OR ketorolac OR meloxicam OR naproxen OR piroxicam OR NSAID OR “non steroidal anti inflammatory” OR colchicine OR allopurinol OR quinine OR levothyroxine OR thyroxine OR liothyronine OR carbimazole OR propylthiouracil OR iodine OR paracetamol OR acetaminophen OR antiglycaemi* OR biguanide OR metformin OR sulphonylurea OR gliclazide OR glibenclamide OR glimepiride OR glipizide OR pioglitazone OR rosiglitazone OR insulin OR acarbose OR alogliptin OR linagliptin OR saxagliptin OR sitagliptin OR vildagliptin OR antiepilep* OR anticonvulsant OR valprotate OR carbamazepine OR barbiturate OR lithium OR lamotrigine OR gabapentin OR phenytoin OR pregabalin OR ethosuximide OR levetiracetam OR topiramate OR vigabatrin OR tiagabine OR antihistamine OR cyclizine OR cyproheptadine OR dexchlorpheniramine OR diphenhydramine OR doxylamine OR pheniramine OR promethazine OR trimeprazine OR cetirizine OR desloratadine OR fexofenadine OR levocetirizine OR loratadine OR diuretic OR thiazide OR frusemide OR indapamide OR hydrochlorothiazide OR spironolactone OR digoxin OR amiodarone OR antiarrhythmi* OR antidepressant* OR SSRI OR “serotonin reuptake inhibitor” OR mirtazapine OR TCA OR “tricyclic antidepressant” OR mianserin OR SNRI OR “serotonin and noradrenaline reuptake inhibitor” OR venlafaxine OR duloxetine OR desvenlafaxine OR “beta blocker” OR betablocker OR “alpha blocker” OR “angiotensin ii receptor antagonist” OR sartan OR “angiotensin converting enzyme inhibitors” OR acei OR statin OR “hmg coa reductase inhibitor”) N3 (deprescri* OR withdraw* OR withdrew OR ceas* OR cessation OR withheld OR withhold OR discontinu* OR reduc* OR taper* OR stop OR stopping OR stopped OR end OR ending OR ended OR remove* OR removal OR removing)) OR AB ((medication* OR medicin* OR prescription* OR prescrib* OR polypharmacy* OR pharmaceutical* OR drug OR drugs OR aperient OR fiber OR laxative* OR lactulose OR glycerol OR sorbitol OR macrogol OR docusate OR bisacodyl OR senna OR picosulfate OR paraffin OR poloxamer OR “hormone replacement therapy” OR *estrogen OR *estradiol OR *estriol OR tibolone OR “direct thrombin inhibitor” OR bivalirudin OR dabigatran OR warfarin OR anticoagula* OR “factor xa inhibitor” OR “factor xa inhibitors” OR apixaban OR fondaparinux OR rivaroxaban OR pyridoxine OR thiamine OR calcitriol OR cholecalciferol OR colecalciferol OR ergocalciferol OR micronutrient OR vitamin OR mineral OR iron OR ferrous OR calcium OR potassium OR magnesium OR ascorb* OR folic OR folate OR hydroxocobalamin OR cyanocobalamin OR “proton pump inhibitor” OR PPI OR “acid suppression” OR pantoprazole OR omeprazole OR esomeprazole OR lansoprazole OR rabeprazole OR antacid OR cimetidine OR famotidine OR nizatidine OR ranitidine OR fenofibrate OR gemfibrozil OR fibrate OR ezetimibe OR corticosteroid OR glucocorticoid OR steroid OR dihydropyridines OR amlodipine OR diltiazem OR felodipine OR lercanidipine OR nifedipine OR nimodipine OR verapamil OR nitrate OR nitrates OR trinitrate OR isosorbide OR mononitrate OR ivabradine OR nicorandil OR perhexiline OR antipsychotic OR amisulpride OR aripiprazole OR asenapine OR chlorpromazine OR clozapine OR droperidol OR flupenthixol OR fluphenazine OR haloperidol OR olanzapine OR paliperidone OR pericyazine OR quetiapine OR risperidone OR trifluoperazine OR ziprasidone OR zuclopenthixol OR lithium OR buspirone OR diphenhydramine OR doxylamine OR melatonin OR zolpidem OR zopiclone OR benzodiazepine OR alprazolam OR bromazepam OR midazolam OR clobazam OR nitrazepam OR oxazepam OR temazepam OR triazolam OR clonazepam OR diazepam OR flunitrazepam OR lorazepam OR hypnotic OR sedative OR anticholinergic OR darifenacin OR oxybutynin OR tolterodine OR benzhexol OR glycopyrronium OR hyoscine OR bromide OR biperiden OR orphenadrine OR solifenacin OR dopamine OR bromocriptine OR cabergoline OR pergolide OR apomorphine OR pramipexole OR rotigotine OR rasagiline OR selegiline OR amantadine OR entacapone OR levodopa OR anticholinesterase OR donepezil OR memantine OR galantamine OR rivastigmine OR bisphosphonate OR raloxifene OR alendronate OR clodronate OR ibandron* OR pamidronate OR risedronate OR tiludronate OR coledron* OR strontium OR aspirin OR antiplatelet OR clopidogrel OR thienopyridines OR prasugrel OR ticlopidine OR dipyridamole OR celecoxib OR diclofenac OR etoricoxib OR ibuprofen OR indomethacin OR ketoprofen OR ketorolac OR meloxicam OR naproxen OR piroxicam OR NSAID OR “non steroidal anti inflammatory” OR colchicine OR allopurinol OR quinine OR levothyroxine OR thyroxine OR liothyronine OR carbimazole OR propylthiouracil OR iodine OR paracetamol OR acetaminophen OR antiglycaemi* OR biguanide OR metformin OR sulphonylurea OR gliclazide OR glibenclamide OR glimepiride OR glipizide OR pioglitazone OR rosiglitazone OR insulin OR acarbose OR alogliptin OR linagliptin OR saxagliptin OR sitagliptin OR vildagliptin OR antiepilep* OR anticonvulsant OR valprotate OR carbamazepine OR barbiturate OR lithium OR lamotrigine OR gabapentin OR phenytoin OR pregabalin OR ethosuximide OR levetiracetam OR topiramate OR vigabatrin OR tiagabine OR antihistamine OR cyclizine OR cyproheptadine OR dexchlorpheniramine OR diphenhydramine OR doxylamine OR pheniramine OR promethazine OR trimeprazine OR cetirizine OR desloratadine OR fexofenadine OR levocetirizine OR loratadine OR diuretic OR thiazide OR frusemide OR indapamide OR hydrochlorothiazide OR spironolactone OR digoxin OR amiodarone OR antiarrhythmi* OR antidepressant* OR SSRI OR “serotonin reuptake inhibitor” OR mirtazapine OR TCA OR “tricyclic antidepressant” OR mianserin OR SNRI OR “serotonin and noradrenaline reuptake inhibitor” OR venlafaxine OR duloxetine OR desvenlafaxine OR “beta blocker” OR betablocker OR “alpha blocker” OR “angiotensin ii receptor antagonist” OR sartan OR “angiotensin converting enzyme inhibitors” OR acei OR statin OR “hmg coa reductase inhibitor”) N3 (deprescri* OR withdraw* OR withdrew OR ceas* OR cessation OR withheld OR withhold OR discontinu* OR reduc* OR taper* OR stop OR stopping OR stopped OR end OR ending OR ended OR remove* OR removal OR removing)))

AND

(TI ("systematic review" OR "systematic literature review" OR “meta analysis” OR “metaanalysis”) OR AB ("systematic review" OR "systematic literature review" OR “meta analysis” OR “metaanalysis”))

Cochrane Database of Systematic Reviews – limited to Cochrane Reviews and Cochrane Protocols, 2005-2020

Title Abstract Keyword: (elder OR elders OR eldest OR elderly OR geriatric* OR “old age” OR “oldest old” OR “senior citizen” OR “senior citizens” OR “very old” OR septuagenarian* OR octogenarian* OR octagenarian* OR nonagenarian* OR centarian* OR centenarian* OR supercentenarian* OR “older people” OR “older person” OR “older subject” OR “older subjects” OR “older patient” OR “older patients” OR “older age” OR “older aged” OR “older ages” OR “older adult” OR “older adults” OR “older man” OR “older men” OR “older male” OR “older males” OR “older woman” OR “older women” OR “older female” OR “older females” OR “older veterans” OR “older population”)

AND

((deprescri* OR “de prescribing” OR “de prescription” OR “inappropriate prescribing” OR “inappropriate prescription” OR “inappropriate prescriptions” OR “over prescribing” OR “inappropriate medications” OR “inappropriate medication”)

OR

((medication* OR medicin* OR prescription* OR prescrib* OR polypharmacy* OR pharmaceutical* OR drug OR drugs OR aperient OR fiber OR laxative* OR lactulose OR glycerol OR sorbitol OR macrogol OR docusate OR bisacodyl OR senna OR picosulfate OR paraffin OR poloxamer OR “hormone replacement therapy” OR estrogen OR estradiol OR estriol OR tibolone OR “direct thrombin inhibitor” OR bivalirudin OR dabigatran OR warfarin OR anticoagula* OR “factor xa inhibitor” OR “factor xa inhibitors” OR apixaban OR fondaparinux OR rivaroxaban OR pyridoxine OR thiamine OR calcitriol OR cholecalciferol OR colecalciferol OR ergocalciferol OR micronutrient OR vitamin OR mineral OR iron OR ferrous OR calcium OR potassium OR magnesium OR ascorb* OR folic OR folate OR hydroxocobalamin OR cyanocobalamin OR “proton pump inhibitor” OR PPI OR “acid suppression” OR pantoprazole OR omeprazole OR esomeprazole OR lansoprazole OR rabeprazole OR antacid OR cimetidine OR famotidine OR nizatidine OR ranitidine OR fenofibrate OR gemfibrozil OR fibrate OR ezetimibe OR corticosteroid OR glucocorticoid OR steroid OR dihydropyridines OR amlodipine OR diltiazem OR felodipine OR lercanidipine OR nifedipine OR nimodipine OR verapamil OR nitrate OR nitrates OR trinitrate OR isosorbide OR mononitrate OR ivabradine OR nicorandil OR perhexiline OR antipsychotic OR amisulpride OR aripiprazole OR asenapine OR chlorpromazine OR clozapine OR droperidol OR flupenthixol OR fluphenazine OR haloperidol OR olanzapine OR paliperidone OR pericyazine OR quetiapine OR risperidone OR trifluoperazine OR ziprasidone OR zuclopenthixol OR lithium OR buspirone OR diphenhydramine OR doxylamine OR melatonin OR zolpidem OR zopiclone OR benzodiazepine OR alprazolam OR bromazepam OR midazolam OR clobazam OR nitrazepam OR oxazepam OR temazepam OR triazolam OR clonazepam OR diazepam OR flunitrazepam OR lorazepam OR hypnotic OR sedative OR anticholinergic OR darifenacin OR oxybutynin OR tolterodine OR benzhexol OR glycopyrronium OR hyoscine OR bromide OR biperiden OR orphenadrine OR solifenacin OR dopamine OR bromocriptine OR cabergoline OR pergolide OR apomorphine OR pramipexole OR rotigotine OR rasagiline OR selegiline OR amantadine OR entacapone OR levodopa OR anticholinesterase OR donepezil OR memantine OR galantamine OR rivastigmine OR bisphosphonate OR raloxifene OR alendronate OR clodronate OR ibandron* OR pamidronate OR risedronate OR tiludronate OR coledron* OR strontium OR aspirin OR antiplatelet OR clopidogrel OR thienopyridines OR prasugrel OR ticlopidine OR dipyridamole OR celecoxib OR diclofenac OR etoricoxib OR ibuprofen OR indomethacin OR ketoprofen OR ketorolac OR meloxicam OR naproxen OR piroxicam OR NSAID OR “non steroidal anti inflammatory” OR colchicine OR allopurinol OR quinine OR levothyroxine OR thyroxine OR liothyronine OR carbimazole OR propylthiouracil OR iodine OR paracetamol OR acetaminophen OR antiglycaemi* OR biguanide OR metformin OR sulphonylurea OR gliclazide OR glibenclamide OR glimepiride OR glipizide OR pioglitazone OR rosiglitazone OR insulin OR acarbose OR alogliptin OR linagliptin OR saxagliptin OR sitagliptin OR vildagliptin OR antiepilep* OR anticonvulsant OR valprotate OR carbamazepine OR barbiturate OR lithium OR lamotrigine OR gabapentin OR phenytoin OR pregabalin OR ethosuximide OR levetiracetam OR topiramate OR vigabatrin OR tiagabine OR antihistamine OR cyclizine OR cyproheptadine OR dexchlorpheniramine OR diphenhydramine OR doxylamine OR pheniramine OR promethazine OR trimeprazine OR cetirizine OR desloratadine OR fexofenadine OR levocetirizine OR loratadine OR diuretic OR thiazide OR frusemide OR indapamide OR hydrochlorothiazide OR spironolactone OR digoxin OR amiodarone OR antiarrhythmi* OR antidepressant* OR SSRI OR “serotonin reuptake inhibitor” OR mirtazapine OR TCA OR “tricyclic antidepressant” OR mianserin OR SNRI OR “serotonin and noradrenaline reuptake inhibitor” OR venlafaxine OR duloxetine OR desvenlafaxine OR “beta blocker” OR betablocker OR “alpha blocker” OR “angiotensin ii receptor antagonist” OR sartan OR “angiotensin converting enzyme inhibitors” OR acei OR statin OR “hmg coa reductase inhibitor”) NEAR/3 (deprescri* OR withdraw* OR withdrew OR ceas* OR cessation OR withheld OR withhold OR discontinu* OR reduc* OR taper* OR stop OR stopping OR stopped OR end OR ending OR ended OR remove* OR removal OR removing)))

Scopus

TITLE-ABS-KEY(elder OR elders OR eldest OR elderly OR geriatric* OR "old age" OR "oldest old" OR "senior citizen" OR "senior citizens" OR "very old" OR septuagenarian* OR octogenarian* OR octagenarian* OR nonagenarian* OR centarian* OR centenarian* OR supercentenarian*) AND TITLE-ABS-KEY((deprescri* OR "de prescribing" OR "de prescription" OR "inappropriate prescribing" OR "inappropriate prescription" OR "inappropriate prescriptions" OR "over prescribing" OR "inappropriate medications" OR "inappropriate medication") OR ((medication* OR medicin* OR prescription* OR prescrib* OR polypharmacy* OR pharmaceutical* OR drug OR drugs OR aperient OR fiber OR laxative* OR lactulose OR glycerol OR sorbitol OR macrogol OR docusate OR bisacodyl OR senna OR picosulfate OR paraffin OR poloxamer OR "hormone replacement therapy" OR estrogen OR estradiol OR estriol OR tibolone OR "direct thrombin inhibitor" OR bivalirudin OR dabigatran OR warfarin OR anticoagula* OR "factor xa inhibitor" OR "factor xa inhibitors" OR apixaban OR fondaparinux OR rivaroxaban OR pyridoxine OR thiamine OR calcitriol OR cholecalciferol OR colecalciferol OR ergocalciferol OR micronutrient OR vitamin OR mineral OR iron OR ferrous OR calcium OR potassium OR magnesium OR ascorb* OR folic OR folate OR hydroxocobalamin OR cyanocobalamin OR "proton pump inhibitor" OR PPI OR "acid suppression" OR pantoprazole OR omeprazole OR esomeprazole OR lansoprazole OR rabeprazole OR antacid OR cimetidine OR famotidine OR nizatidine OR ranitidine OR fenofibrate OR gemfibrozil OR fibrate OR ezetimibe OR corticosteroid OR glucocorticoid OR steroid OR dihydropyridines OR amlodipine OR diltiazem OR felodipine OR lercanidipine OR nifedipine OR nimodipine OR verapamil OR nitrate OR nitrates OR trinitrate OR isosorbide OR mononitrate OR ivabradine OR nicorandil OR perhexiline OR antipsychotic OR amisulpride OR aripiprazole OR asenapine OR chlorpromazine OR clozapine OR droperidol OR flupenthixol OR fluphenazine OR haloperidol OR olanzapine OR paliperidone OR pericyazine OR quetiapine OR risperidone OR trifluoperazine OR ziprasidone OR zuclopenthixol OR lithium OR buspirone OR diphenhydramine OR doxylamine OR melatonin OR zolpidem OR zopiclone OR benzodiazepine OR alprazolam OR bromazepam OR midazolam OR clobazam OR nitrazepam OR oxazepam OR temazepam OR triazolam OR clonazepam OR diazepam OR flunitrazepam OR lorazepam OR hypnotic OR sedative OR anticholinergic OR darifenacin OR oxybutynin OR tolterodine OR benzhexol OR glycopyrronium OR hyoscine OR bromide OR biperiden OR orphenadrine OR solifenacin OR dopamine OR bromocriptine OR cabergoline OR pergolide OR apomorphine OR pramipexole OR rotigotine OR rasagiline OR selegiline OR amantadine OR entacapone OR levodopa OR anticholinesterase OR donepezil OR memantine OR galantamine OR rivastigmine OR bisphosphonate OR raloxifene OR alendronate OR clodronate OR ibandron* OR pamidronate OR risedronate OR tiludronate OR coledron* OR strontium OR aspirin OR antiplatelet OR clopidogrel OR thienopyridines OR prasugrel OR ticlopidine OR dipyridamole OR celecoxib OR diclofenac OR etoricoxib OR ibuprofen OR indomethacin OR ketoprofen OR ketorolac OR meloxicam OR naproxen OR piroxicam OR NSAID OR "non steroidal anti inflammatory" OR colchicine OR allopurinol OR quinine OR levothyroxine OR thyroxine OR liothyronine OR carbimazole OR propylthiouracil OR iodine OR paracetamol OR acetaminophen OR antiglycaemi* OR biguanide OR metformin OR sulphonylurea OR gliclazide OR glibenclamide OR glimepiride OR glipizide OR pioglitazone OR rosiglitazone OR insulin OR acarbose OR alogliptin OR linagliptin OR saxagliptin OR sitagliptin OR vildagliptin OR antiepilep* OR anticonvulsant OR valprotate OR carbamazepine OR barbiturate OR lithium OR lamotrigine OR gabapentin OR phenytoin OR pregabalin OR ethosuximide OR levetiracetam OR topiramate OR vigabatrin OR tiagabine OR antihistamine OR cyclizine OR cyproheptadine OR dexchlorpheniramine OR diphenhydramine OR doxylamine OR pheniramine OR promethazine OR trimeprazine OR cetirizine OR desloratadine OR fexofenadine OR levocetirizine OR loratadine OR diuretic OR thiazide OR frusemide OR indapamide OR hydrochlorothiazide OR spironolactone OR digoxin OR amiodarone OR antiarrhythmi* OR antidepressant* OR SSRI OR "serotonin reuptake inhibitor" OR mirtazapine OR TCA OR "tricyclic antidepressant" OR mianserin OR SNRI OR "serotonin and noradrenaline reuptake inhibitor" OR venlafaxine OR duloxetine OR desvenlafaxine OR "beta blocker" OR betablocker OR "alpha blocker" OR "angiotensin ii receptor antagonist" OR sartan OR "angiotensin converting enzyme inhibitors" OR acei OR statin OR "hmg coa reductase inhibitor") W/3 (deprescri* OR withdraw* OR withdrew OR ceas* OR cessation OR withheld OR withhold OR discontinu* OR reduc* OR taper* OR stop OR stopping OR stopped OR end OR ending OR ended OR remove* OR removal OR removing))) AND TITLE-ABS-KEY(("systematic review" OR "systematic literature review" OR "meta analysis" OR "metaanalysis"))

Web of Science Core Collection

(TI=(elder OR elders OR eldest OR elderly OR geriatric* OR "old age" OR "oldest old" OR "senior citizen" OR "senior citizens" OR "very old" OR septuagenarian* OR octogenarian* OR octagenarian* OR nonagenarian* OR centarian* OR centenarian* OR supercentenarian*) OR AB=(elder OR elders OR eldest OR elderly OR geriatric* OR "old age" OR "oldest old" OR "senior citizen" OR "senior citizens" OR "very old" OR septuagenarian* OR octogenarian* OR octagenarian* OR nonagenarian* OR centarian* OR centenarian* OR supercentenarian*)) AND (TI=(deprescri* OR "de prescribing" OR "de prescription" OR "inappropriate prescribing" OR "inappropriate prescription" OR "inappropriate prescriptions" OR "over prescribing" OR "inappropriate medications" OR "inappropriate medication") OR AB=(deprescri* OR "de prescribing" OR "de prescription" OR "inappropriate prescribing" OR "inappropriate prescription" OR "inappropriate prescriptions" OR "over prescribing" OR "inappropriate medications" OR "inappropriate medication") OR TI=((medication* OR medicin* OR prescription* OR prescrib* OR polypharmacy* OR pharmaceutical* OR drug OR drugs OR aperient OR fiber OR laxative* OR lactulose OR glycerol OR sorbitol OR macrogol OR docusate OR bisacodyl OR senna OR picosulfate OR paraffin OR poloxamer OR "hormone replacement therapy" OR estrogen OR estradiol OR estriol OR tibolone OR "direct thrombin inhibitor" OR bivalirudin OR dabigatran OR warfarin OR anticoagula* OR "factor xa inhibitor" OR "factor xa inhibitors" OR apixaban OR fondaparinux OR rivaroxaban OR pyridoxine OR thiamine OR calcitriol OR cholecalciferol OR colecalciferol OR ergocalciferol OR micronutrient OR vitamin OR mineral OR iron OR ferrous OR calcium OR potassium OR magnesium OR ascorb* OR folic OR folate OR hydroxocobalamin OR cyanocobalamin OR "proton pump inhibitor" OR PPI OR "acid suppression" OR pantoprazole OR omeprazole OR esomeprazole OR lansoprazole OR rabeprazole OR antacid OR cimetidine OR famotidine OR nizatidine OR ranitidine OR fenofibrate OR gemfibrozil OR fibrate OR ezetimibe OR corticosteroid OR glucocorticoid OR steroid OR dihydropyridines OR amlodipine OR diltiazem OR felodipine OR lercanidipine OR nifedipine OR nimodipine OR verapamil OR nitrate OR nitrates OR trinitrate OR isosorbide OR mononitrate OR ivabradine OR nicorandil OR perhexiline OR antipsychotic OR amisulpride OR aripiprazole OR asenapine OR chlorpromazine OR clozapine OR droperidol OR flupenthixol OR fluphenazine OR haloperidol OR olanzapine OR paliperidone OR pericyazine OR quetiapine OR risperidone OR trifluoperazine OR ziprasidone OR zuclopenthixol OR lithium OR buspirone OR diphenhydramine OR doxylamine OR melatonin OR zolpidem OR zopiclone OR benzodiazepine OR alprazolam OR bromazepam OR midazolam OR clobazam OR nitrazepam OR oxazepam OR temazepam OR triazolam OR clonazepam OR diazepam OR flunitrazepam OR lorazepam OR hypnotic OR sedative OR anticholinergic OR darifenacin OR oxybutynin OR tolterodine OR benzhexol OR glycopyrronium OR hyoscine OR bromide OR biperiden OR orphenadrine OR solifenacin OR dopamine OR bromocriptine OR cabergoline OR pergolide OR apomorphine OR pramipexole OR rotigotine OR rasagiline OR selegiline OR amantadine OR entacapone OR levodopa OR anticholinesterase OR donepezil OR memantine OR galantamine OR rivastigmine OR bisphosphonate OR raloxifene OR alendronate OR clodronate OR ibandron* OR pamidronate OR risedronate OR tiludronate OR coledron* OR strontium OR aspirin OR antiplatelet OR clopidogrel OR thienopyridines OR prasugrel OR ticlopidine OR dipyridamole OR celecoxib OR diclofenac OR etoricoxib OR ibuprofen OR indomethacin OR ketoprofen OR ketorolac OR meloxicam OR naproxen OR piroxicam OR NSAID OR "non steroidal anti inflammatory" OR colchicine OR allopurinol OR quinine OR levothyroxine OR thyroxine OR liothyronine OR carbimazole OR propylthiouracil OR iodine OR paracetamol OR acetaminophen OR antiglycaemi* OR biguanide OR metformin OR sulphonylurea OR gliclazide OR glibenclamide OR glimepiride OR glipizide OR pioglitazone OR rosiglitazone OR insulin OR acarbose OR alogliptin OR linagliptin OR saxagliptin OR sitagliptin OR vildagliptin OR antiepilep* OR anticonvulsant OR valprotate OR carbamazepine OR barbiturate OR lithium OR lamotrigine OR gabapentin OR phenytoin OR pregabalin OR ethosuximide OR levetiracetam OR topiramate OR vigabatrin OR tiagabine OR antihistamine OR cyclizine OR cyproheptadine OR dexchlorpheniramine OR diphenhydramine OR doxylamine OR pheniramine OR promethazine OR trimeprazine OR cetirizine OR desloratadine OR fexofenadine OR levocetirizine OR loratadine OR diuretic OR thiazide OR frusemide OR indapamide OR hydrochlorothiazide OR spironolactone OR digoxin OR amiodarone OR antiarrhythmi* OR antidepressant* OR SSRI OR "serotonin reuptake inhibitor" OR mirtazapine OR TCA OR "tricyclic antidepressant" OR mianserin OR SNRI OR "serotonin and noradrenaline reuptake inhibitor" OR venlafaxine OR duloxetine OR desvenlafaxine OR "beta blocker" OR betablocker OR "alpha blocker" OR "angiotensin ii receptor antagonist" OR sartan OR "angiotensin converting enzyme inhibitors" OR acei OR statin OR "hmg coa reductase inhibitor") NEAR/3 (deprescri* OR withdraw* OR withdrew OR ceas* OR cessation OR withheld OR withhold OR discontinu* OR reduc* OR taper* OR stop OR stopping OR stopped OR end OR ending OR ended OR remove* OR removal OR removing)) OR AB=((medication* OR medicin* OR prescription* OR prescrib* OR polypharmacy* OR pharmaceutical* OR drug OR drugs OR aperient OR fiber OR laxative* OR lactulose OR glycerol OR sorbitol OR macrogol OR docusate OR bisacodyl OR senna OR picosulfate OR paraffin OR poloxamer OR "hormone replacement therapy" OR estrogen OR estradiol OR estriol OR tibolone OR "direct thrombin inhibitor" OR bivalirudin OR dabigatran OR warfarin OR anticoagula* OR "factor xa inhibitor" OR "factor xa inhibitors" OR apixaban OR fondaparinux OR rivaroxaban OR pyridoxine OR thiamine OR calcitriol OR cholecalciferol OR colecalciferol OR ergocalciferol OR micronutrient OR vitamin OR mineral OR iron OR ferrous OR calcium OR potassium OR magnesium OR ascorb* OR folic OR folate OR hydroxocobalamin OR cyanocobalamin OR "proton pump inhibitor" OR PPI OR "acid suppression" OR pantoprazole OR omeprazole OR esomeprazole OR lansoprazole OR rabeprazole OR antacid OR cimetidine OR famotidine OR nizatidine OR ranitidine OR fenofibrate OR gemfibrozil OR fibrate OR ezetimibe OR corticosteroid OR glucocorticoid OR steroid OR dihydropyridines OR amlodipine OR diltiazem OR felodipine OR lercanidipine OR nifedipine OR nimodipine OR verapamil OR nitrate OR nitrates OR trinitrate OR isosorbide OR mononitrate OR ivabradine OR nicorandil OR perhexiline OR antipsychotic OR amisulpride OR aripiprazole OR asenapine OR chlorpromazine OR clozapine OR droperidol OR flupenthixol OR fluphenazine OR haloperidol OR olanzapine OR paliperidone OR pericyazine OR quetiapine OR risperidone OR trifluoperazine OR ziprasidone OR zuclopenthixol OR lithium OR buspirone OR diphenhydramine OR doxylamine OR melatonin OR zolpidem OR zopiclone OR benzodiazepine OR alprazolam OR bromazepam OR midazolam OR clobazam OR nitrazepam OR oxazepam OR temazepam OR triazolam OR clonazepam OR diazepam OR flunitrazepam OR lorazepam OR hypnotic OR sedative OR anticholinergic OR darifenacin OR oxybutynin OR tolterodine OR benzhexol OR glycopyrronium OR hyoscine OR bromide OR biperiden OR orphenadrine OR solifenacin OR dopamine OR bromocriptine OR cabergoline OR pergolide OR apomorphine OR pramipexole OR rotigotine OR rasagiline OR selegiline OR amantadine OR entacapone OR levodopa OR anticholinesterase OR donepezil OR memantine OR galantamine OR rivastigmine OR bisphosphonate OR raloxifene OR alendronate OR clodronate OR ibandron* OR pamidronate OR risedronate OR tiludronate OR coledron* OR strontium OR aspirin OR antiplatelet OR clopidogrel OR thienopyridines OR prasugrel OR ticlopidine OR dipyridamole OR celecoxib OR diclofenac OR etoricoxib OR ibuprofen OR indomethacin OR ketoprofen OR ketorolac OR meloxicam OR naproxen OR piroxicam OR NSAID OR "non steroidal anti inflammatory" OR colchicine OR allopurinol OR quinine OR levothyroxine OR thyroxine OR liothyronine OR carbimazole OR propylthiouracil OR iodine OR paracetamol OR acetaminophen OR antiglycaemi* OR biguanide OR metformin OR sulphonylurea OR gliclazide OR glibenclamide OR glimepiride OR glipizide OR pioglitazone OR rosiglitazone OR insulin OR acarbose OR alogliptin OR linagliptin OR saxagliptin OR sitagliptin OR vildagliptin OR antiepilep* OR anticonvulsant OR valprotate OR carbamazepine OR barbiturate OR lithium OR lamotrigine OR gabapentin OR phenytoin OR pregabalin OR ethosuximide OR levetiracetam OR topiramate OR vigabatrin OR tiagabine OR antihistamine OR cyclizine OR cyproheptadine OR dexchlorpheniramine OR diphenhydramine OR doxylamine OR pheniramine OR promethazine OR trimeprazine OR cetirizine OR desloratadine OR fexofenadine OR levocetirizine OR loratadine OR diuretic OR thiazide OR frusemide OR indapamide OR hydrochlorothiazide OR spironolactone OR digoxin OR amiodarone OR antiarrhythmi* OR antidepressant* OR SSRI OR "serotonin reuptake inhibitor" OR mirtazapine OR TCA OR "tricyclic antidepressant" OR mianserin OR SNRI OR "serotonin and noradrenaline reuptake inhibitor" OR venlafaxine OR duloxetine OR desvenlafaxine OR "beta blocker" OR betablocker OR "alpha blocker" OR "angiotensin ii receptor antagonist" OR sartan OR "angiotensin converting enzyme inhibitors" OR acei OR statin OR "hmg coa reductase inhibitor") NEAR/3 (deprescri* OR withdraw* OR withdrew OR ceas* OR cessation OR withheld OR withhold OR discontinu* OR reduc* OR taper* OR stop OR stopping OR stopped OR end OR ending OR ended OR remove* OR removal OR removing))) AND (TI=("systematic review" OR "systematic literature review" OR "meta analysis" OR "metaanalysis") OR AB=("systematic review" OR "systematic literature review" OR "meta analysis" OR "metaanalysis"))

Centre for Reviews & Dissemination (University of York) – Health Technology Assessment, DARE, NHS EED

MESH term=Deprescriptions = 0

MeSH term = Inappropriate Prescribing = 25 [15 new]

Any field = deprescri* AND (older OR aged OR aging OR elder* OR senior OR geriatric*) = 1 [not new]

Any field=(older OR aged OR aging OR elder* OR senior OR geriatric*) AND Any field=inappropriate* AND Any field=(prescribing OR prescription*) = 26

Epistemonikos

(title:((elder OR elders OR eldest OR elderly OR geriatric* OR "old age" OR "oldest old" OR "senior citizen" OR "senior citizens" OR "very old" OR septuagenarian* OR octogenarian* OR octagenarian* OR nonagenarian* OR centarian* OR centenarian* OR supercentenarian*) AND (deprescri* OR "inappropriate prescribing")) OR abstract:((elder OR elders OR eldest OR elderly OR geriatric* OR "old age" OR "oldest old" OR "senior citizen" OR "senior citizens" OR "very old" OR septuagenarian* OR octogenarian* OR octagenarian* OR nonagenarian* OR centarian* OR centenarian* OR supercentenarian*) AND (deprescri* OR "inappropriate prescribing" OR "inappropriate prescriptions" OR "inappropriate medications" OR overprescri*)))
