# Supplementary material for: Deprescribing interventions in older adults: An overview of systematic reviews
Source: PLoS One. 2024 Jun 17;19(6):e0305215. doi: 10.1371/journal.pone.0305215 (PMC11182547; doi:10.1371/journal.pone.0305215)
Supplement: S1 Fig — (PDF) [file pone.0305215.s003.pdf]

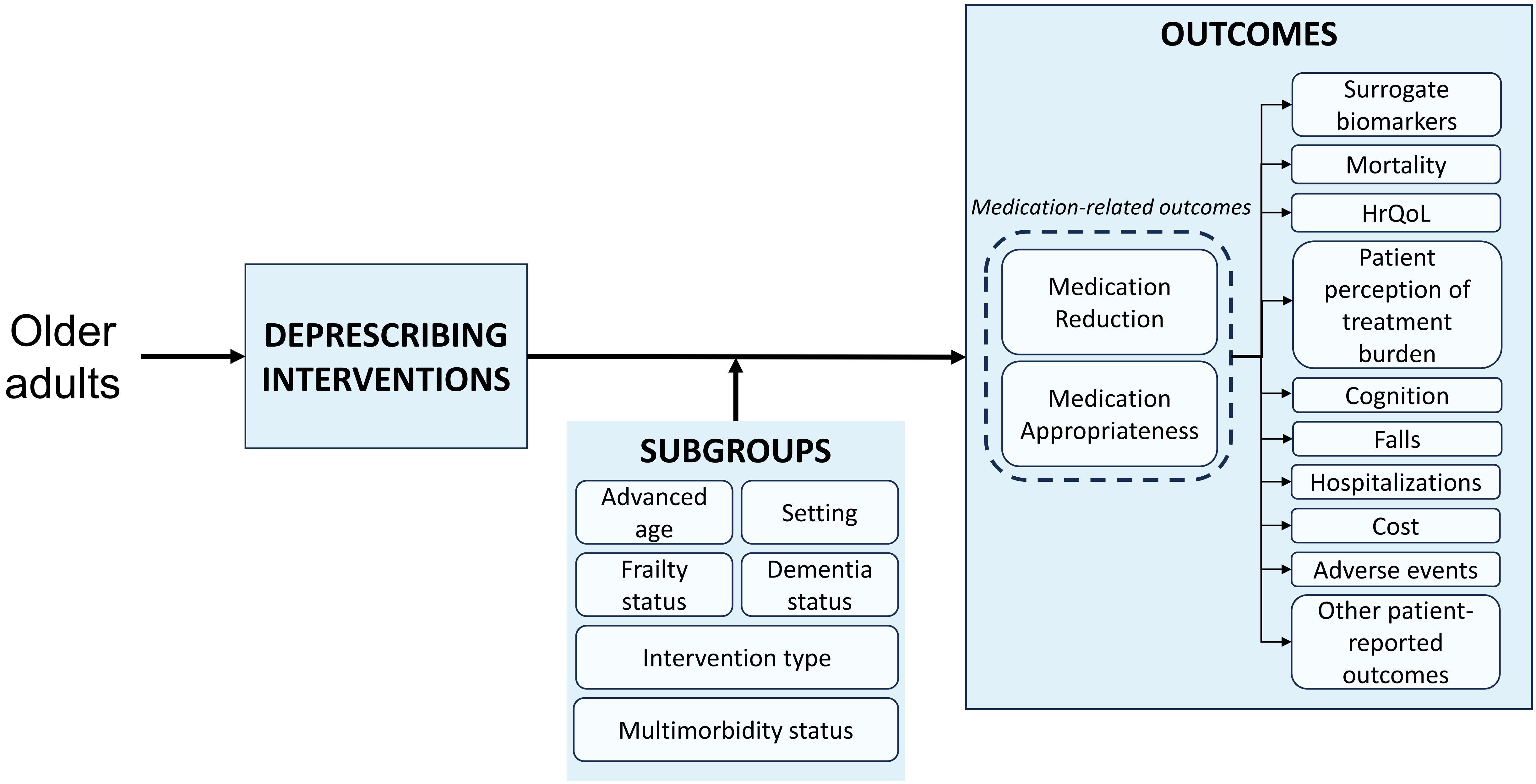
Older adults
DEPRESCRIBING INTERVENTIONS
SUBGROUPS
Advanced age
Setting
Frailty status
Dementia status
Intervention type
Multimorbidity status
OUTCOMES
Medication-related outcomes
Medication Reduction
Medication Appropriateness
Surrogate biomarkers
Mortality
HrQoL
Patient perception of treatment burden
Cognition
Falls
Hospitalizations
Cost
Adverse events
Other patient-reported outcomes
