# Supplementary material for: Deprescribing interventions in older adults: An overview of systematic reviews
Source: PLoS One. 2024 Jun 17;19(6):e0305215. doi: 10.1371/journal.pone.0305215 (PMC11182547; doi:10.1371/journal.pone.0305215)
Supplement: S2 Fig — (DOCX) [file pone.0305215.s004.docx]

S2 Fig. Example of data abstraction for narrative synthesis of eligible studies of systematic reviews for two outcomes (Step 2, table 4)

**STEP 2: Synthesis of eligible primary studies in reviews**

**STEP 1: Summary at Review level**

**Medication Discontinuation**

**Excluded primary studies (n=3):**

- Huijbers 2016– reason: mean age <60 years
- Lewin 2012– reason: mean age <60 years
- Zwisler 2015– reason: mean age <60 years

**Systematic Review:** Thio 2018

Primary studies (n=26)

**Included:**

Under the paragraph *‘Primary outcomes’* – “two studies found a significant difference in favour of the intervention group, … one (Potter 2016 at the number of medicines successfully discontinued”

– reason: SS reported, and Potter 2016 is an eligible primary study

**Excluded:**

Under the paragraph *‘Adverse events’* – “and seven studies reported that adverse events were rare and no serious adverse events occurred.” – Reason: SSNR

Under the paragraph *‘Adverse events’* – “One study (Lewin 2012) reported more serious adverse events in the control group but did not report significance calculation.” – Reason: SSNR, and Lewin 2012 is not an eligible primary study.

Under the paragraph *‘Restart of medication and relapse of symptoms’* – “The other eight studies reported relapse for only the intervention group or for the entire research population…and seven did not perform statistical testing of the observed difference.” – Reason: did not compare between groups.

**Excluded:**

Under the paragraph *‘Success of medication cessation’* and Table 2.

– Reason: SSNR

**Included:**

Under the paragraph *‘Adverse events’* – “Eight studies found no significant difference between comparator groups” (entered data under ADR)

Under the paragraph *‘Restart of medication and relapse of symptoms’* – “Nine studies reported rate of relapse for both placebo and control groups. Five of these studies found a significant difference between both groups, with more relapse in the intervention group compared with the control group.” (entered data under ADWE)

**RESULT entered in Table 3: (A) Evidence suggested beneficial effect only**

**RESULT entered in Table 3: ADR (C) Evidence suggested no effect. ADWE (F) Evidence suggested a negative effect.**

**Eligible primary studies (n=23)**

**Example of data abstraction for two outcomes**

ADR, adverse drug reaction; ADWE, adverse drug withdrawal event; SSNR, statistical significance not reported in narrative synthesis of systematic review; SS, statistical significance testing was reported in narrative synthesis of systematic review.
