# Supplementary material for: Deprescribing interventions in older adults: An overview of systematic reviews
Source: PLoS One. 2024 Jun 17;19(6):e0305215. doi: 10.1371/journal.pone.0305215 (PMC11182547; doi:10.1371/journal.pone.0305215)
Supplement: S1 Table — (DOCX) [file pone.0305215.s005.docx]

**S1 Table. Amendments to protocol**

| Protocol Section | Amendment | Rationale |
| --- | --- | --- |
| Population | Lowered inclusion criteria to greater than 60 years or older (from 65 years of age or older). | During the abstract and full-text screening, we found relevant reviews that did not meet the original age cut-off: 1) Reviews that focused on nursing facilities even if age as was not often an eligibility criterion; and 2) reviews that did not focus on older adults, but data was presented to identify primary studies where the mean age ≥60. |
| Inclusion criteria | Added an additional inclusion criterion that reviews needed to have a medication-related outcome. | During the abstract and full-text screening, we determined that documenting evidence of medication reduction is needed to interpret effects on other downstream outcomes (clinical, biomarkers, adverse events, etc.) |
| Exclusion criteria | Reviews that included <2 primary studies that met the PICOS | As we were abstracting data, we realized that not all primary studies of the included reviews were relevant (*e.g.,* did not use a eligible study design, age <60, did not include a comparator group, etc.). We excluded reviews that had only 1 eligible study. |
| Outcomes | Added cost as an outcome. | We adapted the core outcomes developed for polypharmacy for use in this overview. |
| Subgroup | We stated we would examine outcomes according to number of medications. | This subgroup description is not relevant for studies that focused on specific medication classes. Of general deprescribing studies, no reviews presented data according to number of medications. |
| Data abstraction | Clarification on how we included reviews in the narrative synthesis. Narrative synthesis of the overview only included results from primary studies of included reviews that met the PICOS criteria of the overview. | As we were abstracting data, we realized that not all primary studies of the included reviews were relevant (*e.g.,* did not use a eligible study design, age <60, did not include a comparator group, etc.). These were considered ineligible studies and data were not abstracted from the review for these for Table 3. |
| Expert Panel | We convened an interprofessional expert panel who represented a broad range of clinical expertise and perspectives related to the care of older adults. The panel provided input on key steps, including the design, interpretation of results and manuscript review. | A priori we planned to utilize an Expert Panel for broader input but inadvertently left this information out of the protocol. |
| Inclusion of results of meta-analyses | Not included in the original protocol, we present the results for meta-analyses of reviews. | The addition of meta-analyzed results provides another level of information to complement our narrative synthesis. |
