# Supplementary material for: Deprescribing interventions in older adults: An overview of systematic reviews
Source: PLoS One. 2024 Jun 17;19(6):e0305215. doi: 10.1371/journal.pone.0305215 (PMC11182547; doi:10.1371/journal.pone.0305215)
Supplement: S3 Table — (DOCX) [file pone.0305215.s007.docx]

S3 Table. List of articles excluded at full-text, with reasons

|  | **Reference** | **Author, Year** | **Reason for Exclusion** | **Additional Comments** |
| --- | --- | --- | --- | --- |
| 1. | Interventions to improve prescribing quality in care homes: a systematic review | Loganathan, 2010 | Published in abstract / poster form only | Conference poster |
| 2. | Computerised Medication Analysis Designed to Minimise Inappropriate Prescribing in Older Hospitalised Patients: A Systematic Review and Meta-Analysis | Dalton, 2017 | Published in abstract / poster form only | Conference poster |
| 3. | Changing Behaviours: A Systematic Literature Review of Deprescribing Interventions in Older People | Raae-Hansen, 2017 | Published in abstract / poster form only | Conference poster; Full-text is included in our overview |
| 4. | Risk for major and fatal bleeding after discontinuing oral anticoagulation for unprovoked venous thromboembolism: Systematic review and meta-analysis | Khan F, 2019 | Published in abstract / poster form only | Systematic review protocol |
| 5. | A systematic review identifying the impact of patient decision aids in older patients with problematic polypharmacy | Khan AH, 2019 | Published in abstract / poster form only | Conference poster |
| 6. | Polypharmacy, risk of falls in older people and deprescribing: A literature review | Morgado, 2017 | Published in abstract / poster form only | Conference poster |
| 7. | Nonpharmacologic outpatient interventions for benzodiazepine discontinuation in elderly persons | Arsenault, 2015 | Published in abstract / poster form only | Conference poster |
| 8. | Deprescribing strategies in elderly patients or patients with several chronic conditions | Rodriguez-Perez, 2014 | Published in abstract / poster form only | Conference poster |
| 9. | Interventions to improve prescribing at the end-of-life: A systematic review | Velten, 2011 | Published in abstract / poster form only | Conference poster |
| 10. | Does antipsychotic dose reduction result in worsening behavior among nursing home residents with dementia: A systematic review of the literature? | Tjia, 2012 | Published in abstract / poster form only | Conference poster |
| 11. | Deprescribing of medications in older patients in the last three months of life: A systematic review | Alwidyan, 2020 | Published in abstract / poster form only | Conference poster |
| 12. | Inappropriate hospitalisations, aggressive treatments, and insufficient deprescribing: The medicalisation of death from natural causes | Cardona, 2019 | Published in abstract / poster form only | Conference poster |
| 13. | A systematic review and new categorization of clinical aids to improve pharmacotherapy in older patients | Wehling | Published in abstract / poster form only | Article or poster was not found |
| 14. | A systematic review and thematic synthesis of evaluations of pharmacy-led services in care homes across England | Rathbone, 2020 | Published in abstract / poster form only | Conference poster |
| 15. | Evaluation of Deprescribing Services in Frail Patients: A Systematic Review | Alshatti, 2022 | Published in abstract / poster form only | Conference poster |
| 16. | Effectiveness of intervention-based opioid de-prescribing strategies for patients with chronic non-cancer pain in primary care settings: A systematic review and meta-analysis | Cai, 2022 | Published in abstract / poster form only | Conference abstract |
| 17. | A Systematic Review of the Effects of Improvement of Prescription to Reduce the Number of Medications in the Elderly with Polypharmacy | Maeda, 2009 | Not in English |  |
| 18. | Risks and benefits of statin deprescription in palliative care: A systemic review of the literature | Málaga, 2020 | Not in English |  |
| 19. | Review of computerized clinical decision support in community pharmacy | Curtain, 2014 | Not a systematic review as set out by DARE criteria^a^ |  |
| 20. | Evidence-Based Strategies for the Optimization of Pharmacotherapy in Older People | Topinkova, 2012 | Not a systematic review as set out by DARE criteria |  |
| 21. | Medication errors in older adults: a review of recent publications | Aspinall, 2007 | Not a systematic review as set out by DARE criteria |  |
| 22. | Deprescribing antihyperglycemic agents in older persons: Evidence-based clinical practice guideline | Farrell, 2017 | Not a systematic review as set out by DARE criteria |  |
| 23. | Review: Minimal interventions (e.g., a letter) reduce long-term benzodiazepine use in primary care | Primack, 2012 | Not a systematic review as set out by DARE criteria |  |
| 24. | Deprescribing in older people | Liacos, 2020 | Not a systematic review as set out by DARE criteria |  |
| 25. | Intervention elements to reduce inappropriate prescribing for older adults with multimorbidity receiving outpatient care: A scoping review | Lee, 2020 | Not a systematic review as set out by DARE criteria |  |
| 26. | Multidisciplinary Medication Review in Long-Term Care: A Review of Clinical Utility, Cost-Effectiveness and Guidelines | Chao, 2019 | Not a systematic review as set out by DARE criteria |  |
| 27. | Rates, determinants and success of implementing deprescribing in people with type 2 diabetes: A scoping review | Oktora, 2020 | Not a systematic review as set out by DARE criteria |  |
| 28. | For which patient subgroups are there positive outcomes from a medication review? A systematic review | Abrahamsen, 2020 | Not a systematic review as set out by DARE criteria |  |
| 29. | Pharmacist interventions to deprescribe opioids and benzodiazepines in older adults: A rapid review | Niznik, 2022 | Not a systematic review as set out by DARE criteria |  |
| 30. | The Effect of Interventions to Reduce Potentially Inappropriate Antibiotic Prescribing in Long-Term Care Facilities: a Systematic Review of Randomised Controlled Trials | Fleming, 2013 | No relevant study population | Age of population not defined in study |
| 31. | Reduced-dose direct oral anticoagulants in the extended treatment of venous thromboembolism: a systematic review and meta-analysis | Vasanthamohan, 2018 | No relevant study population | Age of population not defined in study |
| 32. | Reduction or discontinuation of antipsychotics for challenging behaviour in adults with intellectual disability: a systematic review | Sheehan, 2017 | No relevant study population | Age of population <60 years old |
| 33. | Strategies for discontinuation of proton pump inhibitors: a systematic review | Haastrup, 2014 | No relevant study population | Age of population not defined in study |
| 34. | The risk of asthma exacerbation after stopping low-dose inhaled corticosteroids: a systematic review and meta-analysis of randomized controlled trials | Rank, 2013 | No relevant study population | Age of population <60 years old |
| 35. | Deprescribing Medications for Chronic Diseases Management in Primary Care Settings: A Systematic Review of Randomized Controlled Trials | Dills, 2018 | No relevant study population | Age of population <60 years old |
| 36. | Minimal interventions to decrease long-term use of benzodiazepines in primary care: a systematic review and meta-analysis | Mugunthan, 2011 | No relevant study population | Age of population <60 years old |
| 37. | Stepping down the dose of inhaled corticosteroids for adults with asthma | Crossingham, 2017 | No relevant study population | Age of population <60 years old |
| 38. | Deprescribing versus continuation of chronic proton pump inhibitor use in adults | Boghossian, 2017 | No relevant study population | Age of population <60 years old |
| 39. | The effectiveness of computer system tools on potentially inappropriate medications ordered at discharge for adults older than 65 years of age: a systematic review | Iankowitz, 2012 | No relevant intervention |  |
| 40. | The effect of pharmacist-led interventions in optimising prescribing in older adults in primary care: A systematic review | Riordan, 2016 | No relevant intervention |  |
| 41. | Reducing potentially inappropriate medications in palliative cancer patients: evidence to support deprescribing approaches | Lindsay, 2014 | No relevant intervention |  |
| 42. | Analgesics use and withdrawal in people with dementia - a register-based Danish study and a systematic review | Sørensen, 2019 | No relevant intervention |  |
| 43. | Hospital pharmacists working with geriatric patients in Europe: a systematic literature review | Kiesel, 2018 | No relevant intervention |  |
| 44. | Risk versus risk: a review of benzodiazepine reduction in older adults | Paquin, 2014 | No relevant intervention |  |
| 45. | Discontinuation of lithium augmentation in geriatric patients with unipolar depression: A systematic review | Ross, 2008 | No relevant intervention |  |
| 46. | Discontinuation of alpha-blocker therapy in men with lower urinary tract symptoms: a systematic review and meta-analysis | van der Worp, 2019 | No relevant intervention |  |
| 47. | The Effect of Melatonin on Benzodiazepine Discontinuation and Sleep Quality in Adults Attempting to Discontinue Benzodiazepines: A Systematic Review and Meta-Analysis | Wright, 2015 | No relevant intervention |  |
| 48. | Clinical medication review of polypharmacy reduced unplanned hospitalizations in older adults: A meta-analysis of randomized clinical trials | Mizokami, 2019 | No relevant intervention |  |
| 49. | The feasibility of Chinese massage as an auxiliary way of replacing or reducing drugs in the clinical treatment of adult type 2 diabetes: A systematic review and meta-analysis | Zhang, 2020 | No relevant intervention |  |
| 50. | Long-Term Drug Therapy and Drug Discontinuations and Holidays for Osteoporosis Fracture Prevention A Systematic Review | Fink, 2019 | No relevant intervention |  |
| 51. | Polypharmacy and mobility outcomes | Ali, 2020 | No relevant intervention |  |
| 52. | Deprescribing interventions and their impact on medication adherence in community-dwelling older adults with polypharmacy: a systematic review | Ulley, 2019 | No relevant intervention |  |
| 53. | Potentially inappropriate prescribing in older adults in central and Eastern Europe: systematic literature review | Brkic, 2022 | No relevant intervention |  |
| 54. | A Systematic Review of Interventions to Improve Analgesic Use and Appropriateness in Long-Term Care Facilities | Dowd, 2022 | No relevant intervention |  |
| 55. | Effectiveness of the STOPP/START (Screening Tool of Older Persons' potentially inappropriate Prescriptions/Screening Tool to Alert doctors to the Right Treatment) criteria: systematic review and meta-analysis of randomized controlled studies | Hill-Taylor, 2016 | No deprescribing outcome of interest |  |
| 56. | Does Deprescribing Improve Quality of Life? A Systematic Review of the Literature | Pruskowski, 2019 | No deprescribing outcome of interest |  |
| 57. | Effectiveness of interventions targeting antibiotic use in long-term aged care facilities: A systematic review and meta-analysis | Raban, 2020 | No deprescribing outcome of interest |  |
| 58. | Withdrawal versus continuation of long‐term antipsychotic drug use for behavioural and psychological symptoms in older people with dementia | Van Leeuwen, 2018 | No deprescribing outcome of interest |  |
| 59. | Pharmacist-led interventions to reduce adverse drug events in older people living in residential aged care facilities: A systematic review | Ali, 2021 | No deprescribing outcome of interest |  |
| 60. | Medication review in hospitalised patients to reduce morbidity and mortality | Bülow, 2023 | No deprescribing outcome of interest |  |
| 61. | Anti-hypertensive drugs deprescribing: an updated systematic review of clinical trials | Crisafulli, 2021 | No deprescribing outcome of interest |  |
| 62. | Medication review interventions to reduce hospital readmissions in older people | Dautzenberg, 2021 | No deprescribing outcome of interest |  |
| 63. | Discontinuation of bisphosphonates in seniors: a systematic review on health outcomes | Lamarre, 2021 | No deprescribing outcome of interest |  |
| 64. | Deprescribing fall-risk increasing drugs (FRIDs) for the prevention of falls and fall-related complications: a systematic review and meta-analysis | Lee, 2021 | No deprescribing outcome of interest |  |
| 65. | Withdrawal or continuation of cholinesterase inhibitors or memantine or both, in people with dementia | Parsons, 2021 | No deprescribing outcome of interest |  |
| 66. | A Systematic Review of the Current Evidence from Randomised Controlled Trials on the Impact of Medication Optimisation or Pharmacological Interventions on Quantitative Measures of Cognitive Function in Geriatric Patients | Pazan, 2022 | No deprescribing outcome of interest |  |
| 67. | Deprescribing Interventions among Community-Dwelling Older Adults: A Systematic Review of Economic Evaluations | Romano, 2022 | No deprescribing outcome of interest |  |
| 68. | Medication reviews and deprescribing as a single intervention in falls prevention: a systematic review and meta-analysis | Seppala, 2022 | No deprescribing outcome of interest |  |
| 69. | Interventions to Reduce Adverse Drug Event-Related Outcomes in Older Adults: A Systematic Review and Meta-analysis | Tecklenborg, 2020 | No deprescribing outcome of interest |  |
| 70. | Pharmacist-led intervention on chronic pain management: A systematic review and meta-analysis | Thapa, 2021 | No deprescribing outcome of interest |  |
| 71. | Approaches for discontinuation versus continuation of long-term antidepressant use for depressive and anxiety disorders in adults | VanLeeuwen, 2021 | No deprescribing outcome of interest |  |
| 72. | Impact of pharmacist-led home medicines review services on drug-related problems among the elderly population: a systematic review | Gudi, 2019 | No relevant control group |  |
| 73. | Interventions for reducing benzodiazepine use in older people: meta-analysis of randomised controlled trials | Gould, 2014 | No relevant control group |  |
| 74. | Discontinuation of Preventive Medicines in Older People with Limited Life Expectancy: A Systematic Review | Narayan, 2017 | No relevant control group |  |
| 75. | Deintensification of hypoglycaemic medications-use of a systematic review approach to highlight safety concerns in older people with type 2 diabetes | Abdelhafiz, 2018 | Fewer than 2 eligible primary studies |  |
| 76. | Effect of interventions to reduce potentially inappropriate use of drugs in nursing homes: a systematic review of randomised controlled trials | Forsetlund, 2011 | Fewer than 2 eligible primary studies |  |
| 77. | Psychosocial interventions for reducing antipsychotic medication in care home residents | Richter, 2012 | Fewer than 2 eligible primary studies |  |
| 78. | Health Outcomes of Deprescribing Interventions Among Older Residents in Nursing Homes: A Systematic Review and Meta-analysis | Kua, 2019 | Fewer than 2 eligible primary studies |  |
| 79. | Antihypertensive withdrawal for the prevention of cognitive decline | Jongstra, 2016 | Fewer than 2 eligible primary studies |  |
| 80. | Process, impact and outcomes of medication review in Australian residential aged care facilities: A systematic review | Chen, 2019 | Fewer than 2 eligible primary studies |  |
| 81. | Probing pharmacists' interventions in Long-Term Care: a systematic review | Goncalves, 2021 | Fewer than 2 eligible primary studies |  |
| 82. | Benefits and Harms of Deprescribing Antihyperglycemics for Adults With Type 2 Diabetes: A Systematic Review | Deng, 2022 | Fewer than 2 eligible primary studies |  |
| 83. | Impact of pharmaceutical care interventions on multidisciplinary care of older patients with cancer: A systematic review | Herledan, 2023 | Fewer than 2 eligible primary studies |  |
| 84. | Impact of anticholinergic discontinuation on cognitive outcomes in older people: a systematic review | Salahudeen, 2014 | Fewer than 2 eligible primary studies |  |
| 85. | The Effectiveness of Interventions to Evaluate and Reduce Healthcare Costs of Potentially Inappropriate Prescriptions among the Older Adults: A Systematic Review | Mucherino, 2022 | Fewer than 2 eligible primary studies |  |
| 86. | Discontinuation of Long-Term Antipsychotic Drug Use for Behavioral and Psychological Symptoms in Older Adults Aged 65 Years and Older With Dementia | Van Leeuwen, 2018 | Duplicate study | Duplicate of #58, published separately |
| 87. | Medication review in hospitalised patients to reduce morbidity and mortality | Christensen, 2016 | Duplicate study | Duplicate of Bulow 2023, which was excluded due to having no deprescribing outcome of interest |

Reasons for exclusion were determined *a priori* in a hierarchical order as follow: 1. Published in abstract / poster form only, 2. Not in English, 3. Not a systematic review as set out by DARE criteria, 4. No relevant study population, 5. No relevant intervention, 6. No deprescribing outcome of interest, 7. No relevant control group, 8. Fewer than 2 eligible primary studies, and 9. Duplicate study. ^a^The Database of Abstracts of Reviews of Effects (DARE) University of York: NIHR Centre for Reviews and Dissemination; 2002 [Available from: <https://www.crd.york.ac.uk/CRDWeb/>.

**References (alphabetical order)**

Abdelhafiz AH, Sinclair AJ. Deintensification of hypoglycaemic medications-use of a systematic review approach to highlight safety concerns in older people with type 2 diabetes. J Diabetes Complications. 2018;32(4):444-50.

Abrahamsen B, Hansen RN, Rossing C. For which patient subgroups are there positive outcomes from a medication review? A systematic review. Pharm Pract (Granada). 2020;18(4):1976.

Ali MU, Sherifali D, Fitzpatrick-Lewis D, Kenny M, Liu A, Lamarche L, et al. Polypharmacy and mobility outcomes. Mech Ageing Dev. 2020;192:111356.

Ali S, Salahudeen MS, Bereznicki LRE, Curtain CM. Pharmacist-led interventions to reduce adverse drug events in older people living in residential aged care facilities: A systematic review. Br J Clin Pharmacol. 2021;87(10):3672-89.

Alshatti DC, A. R.; Hirsch, C.; Cheng, V. Evaluation of Deprescribing Services in Frail Patients: A Systematic Review. Drug Safety. 2022;45(10):1.

Alwidyan T, Parsons C. Deprescribing of medications in older patients in the last three months of life: A systematic review. International Journal of Pharmacy Practice. 2020;28(Supplement_1):44-87.

Arsenault C, Boivin A, Marcoux I, Toupin M. Nonpharmacologic outpatient interventions for benzodiazepine discontinuation in elderly persons. Can Fam Physician. 2014;61(2):S1-S76.

Aspinall S, Sevick MA, Donohue J, Maher R, Hanlon JT. Medication errors in older adults: a review of recent publications. Am J Geriatr Pharmacother. 2007;5(1):75-84.

Boghossian TA, Rashid FJ, Thompson W, Welch V, Moayyedi P, Rojas-Fernandez C, et al. Deprescribing versus continuation of chronic proton pump inhibitor use in adults. Cochrane Database Syst Rev. 2017;3(3):CD011969.

Brkic J, Fialova D, Okuyan B, Kummer I, Sesto S, Capiau A, et al. Prevalence of potentially inappropriate prescribing in older adults in Central and Eastern Europe: a systematic review and synthesis without meta-analysis. Sci Rep. 2022;12(1):16774.

Bulow C, Clausen SS, Lundh A, Christensen M. Medication review in hospitalised patients to reduce morbidity and mortality. Cochrane Database Syst Rev. 2023;1(1):CD008986.

Cai Q, Chen, T.-C., Baig, H., Starr, E., Steinke, D., and Chen, L.-C. Effectiveness of intervention-based opioid de-prescribing strategies for patients with chronic non-cancer pain in primary care settings: A systematic review and meta-analysis. Pharmacoepidemiology & Drug Safety: International Society for Pharmacoepidemiology; 2022.

Cardona M LE, Stehlik P, Sun S, Clark J. Inappropriate hospitalisations, aggressive treatments, and insufficient deprescribing: the medicalisation of death from natural causes. BMJ Evidence-Based Medicine; 2019.

Chao YS, MacDougall D. Multidisciplinary Medication Review in Long-Term Care: A Review of Clinical Utility, Cost-Effectiveness and Guidelines. Multidisciplinary Medication Review in Long-Term Care: A Review of Clinical Utility, Cost-Effectiveness and Guidelines. CADTH Rapid Response Reports. Ottawa (ON)2019.

Chen EYH, Wang KN, Sluggett JK, Ilomaki J, Hilmer SN, Corlis M, Bell JS. Process, impact and outcomes of medication review in Australian residential aged care facilities: A systematic review. Australas J Ageing. 2019;38 Suppl 2:9-25.

Christensen M, Lundh A. Medication review in hospitalised patients to reduce morbidity and mortality. Cochrane Database Syst Rev. 2016;2(2):CD008986.

Crisafulli S, Luxi N, Coppini R, Capuano A, Scavone C, Zinzi A, et al. Anti-hypertensive drugs deprescribing: an updated systematic review of clinical trials. BMC Fam Pract. 2021;22(1):208.

Crossingham I, Evans DJ, Halcovitch NR, Marsden PA. Stepping down the dose of inhaled corticosteroids for adults with asthma. Cochrane Database Syst Rev. 2017;2:CD011802.

Curtain C, Peterson GM. Review of computerized clinical decision support in community pharmacy. J Clin Pharm Ther. 2014;39(4):343-8.

Dalton K, O'Brien GL, Byrne S. Computerised Medication Analysis Designed to Minimise Inappropriate Prescribing in Older Hospitalised Patients: A Systematic Review and Meta-Analysis. Age Ageing. 2017;46:iii51-2.

Dautzenberg L, Bretagne L, Koek HL, Tsokani S, Zevgiti S, Rodondi N, et al. Medication review interventions to reduce hospital readmissions in older people. J Am Geriatr Soc. 2021;69(6):1646-58.

Deng Z, Thompson W, Korenvain C, Lega IC, Farrell B, Lochnan H, McCarthy LM. Benefits and Harms of Deprescribing Antihyperglycemics for Adults With Type 2 Diabetes: A Systematic Review. Can J Diabetes. 2022;46(5):473-9.

Dills H, Shah K, Messinger-Rapport B, Bradford K, Syed Q. Deprescribing Medications for Chronic Diseases Management in Primary Care Settings: A Systematic Review of Randomized Controlled Trials. J Am Med Dir Assoc. 2018;19(11):923-35 e2.

Dowd LA, Cross AJ, Veal F, Ooi CE, Bell JS. A Systematic Review of Interventions to Improve Analgesic Use and Appropriateness in Long-Term Care Facilities. J Am Med Dir Assoc. 2022;23(1):33-43 e3.

Farrell B, Black C, Thompson W, McCarthy L, Rojas-Fernandez C, Lochnan H, et al. Deprescribing antihyperglycemic agents in older persons: Evidence-based clinical practice guideline. Can Fam Physician. 2017;63(11):832-43.

Fink HA, MacDonald R, Forte ML, Rosebush CE, Ensrud KE, Schousboe JT, et al. Long-Term Drug Therapy and Drug Discontinuations and Holidays for Osteoporosis Fracture Prevention: A Systematic Review. Ann Intern Med. 2019;171(1):37-50.

Fleming A, Browne J, Byrne S. The effect of interventions to reduce potentially inappropriate antibiotic prescribing in long-term care facilities: a systematic review of randomised controlled trials. Drugs Aging. 2013;30(6):401-8.

Forsetlund L, Eike MC, Gjerberg E, Vist GE. Effect of interventions to reduce potentially inappropriate use of drugs in nursing homes: a systematic review of randomised controlled trials. BMC Geriatr. 2011;11:16.

Goncalves JR, Ramalhinho I, Sleath BL, Lopes MJ, Cavaco AM. Probing pharmacists' interventions in Long-Term Care: a systematic review. Eur Geriatr Med. 2021;12(4):673-93.

Gould RL, Coulson MC, Patel N, Highton-Williamson E, Howard RJ. Interventions for reducing benzodiazepine use in older people: meta-analysis of randomised controlled trials. Br J Psychiatry. 2014;204(2):98-107.

Gudi SK, Kashyap A, Chhabra M, Rashid M, Tiwari KK. Impact of pharmacist-led home medicines review services on drug-related problems among the elderly population: a systematic review. Epidemiol Health. 2019;41:e2019020.

Haastrup P, Paulsen MS, Begtrup LM, Hansen JM, Jarbol DE. Strategies for discontinuation of proton pump inhibitors: a systematic review. Fam Pract. 2014;31(6):625-30.

Herledan C, Cerfon MA, Baudouin A, Larbre V, Lattard C, Poletto N, et al. Impact of pharmaceutical care interventions on multidisciplinary care of older patients with cancer: A systematic review. J Geriatr Oncol. 2023;14(4):101450.

Hill-Taylor B, Walsh KA, Stewart S, Hayden J, Byrne S, Sketris IS. Effectiveness of the STOPP/START (Screening Tool of Older Persons' potentially inappropriate Prescriptions/Screening Tool to Alert doctors to the Right Treatment) criteria: systematic review and meta-analysis of randomized controlled studies. J Clin Pharm Ther. 2016;41(2):158-69.

Iankowitz N, Dowden M, Palomino S, Uzokwe H, Worral P. The effectiveness of computer system tools on potentially inappropriate medications ordered at discharge for adults older than 65 years of age: a systematic review. JBI Libr Syst Rev. 2012;10(13):798-831.

Jongstra S, Harrison JK, Quinn TJ, Richard E. Antihypertensive withdrawal for the prevention of cognitive decline. Cochrane Database Syst Rev. 2016;11:CD011971.

Khan AH TC, Bimpong KA, Slight SP. A systematic review identifying the impact of patient decision aids in older patients with problematic polypharmacy. In: Practice IJoP, editor.2019.

Khan F, Kimpton M, Tritschler T, Le Gal G, Hutton B, Fergusson DA, Rodger MA. Risk of major bleeding during extended oral anticoagulation in patients with first unprovoked venous thromboembolism: a systematic review and meta-analysis protocol. Syst Rev. 2019;8(1):245.

Kiesel E, Hopf Y. Hospital pharmacists working with geriatric patients in Europe: a systematic literature review. Eur J Hosp Pharm. 2018;25(e1):e74-e81.

Kua CH, Mak VSL, Huey Lee SW. Health Outcomes of Deprescribing Interventions Among Older Residents in Nursing Homes: A Systematic Review and Meta-analysis. J Am Med Dir Assoc. 2019;20(3):362-72 e11.

Lamarre M, Marcotte M, Laurin D, Furrer D, Vedel I, Tourigny A, et al. Discontinuation of bisphosphonates in seniors: a systematic review on health outcomes. Arch Osteoporos. 2021;16(1):133.

Lee J, Negm A, Peters R, Wong EKC, Holbrook A. Deprescribing fall-risk increasing drugs (FRIDs) for the prevention of falls and fall-related complications: a systematic review and meta-analysis. BMJ Open. 2021;11(2):e035978.

Lee JQ, Ying K, Lun P, Tan KT, Ang W, Munro Y, Ding YY. Intervention elements to reduce inappropriate prescribing for older adults with multimorbidity receiving outpatient care: a scoping review. BMJ Open. 2020;10(8):e039543.

Liacos M, Page AT, Etherton-Beer C. Deprescribing in older people. Aust Prescr. 2020;43(4):114-20.

Lindsay J, Dooley M, Martin J, Fay M, Kearney A, Barras M. Reducing potentially inappropriate medications in palliative cancer patients: evidence to support deprescribing approaches. Support Care Cancer. 2014;22(4):1113-9.

Loganathan M, Singh S, Bottle A, Franklin BD, Majeed A. P26 Interventions to improve prescribing quality in care homes: a systematic review. Journal of Epidemiology & Community Health. 2010;64(Suppl 1):A43-A4.

Maeda K. Systematic review of the effects of improvement of prescription to reduce the number of medications in the elderly with polypharmacy. Yakugaku Zasshi. 2009;129(5):631-45.

Malaga LRMG, Marta D; Vega, Diana C F. Risks and benefits of statin deprescription in palliative care: A systemic review of the literature. Medicina Paliativa. 2020;28(1):9.

Mizokami F, Mizuno T, Kanamori K, Oyama S, Nagamatsu T, Lee JK, Kobayashi T. Clinical medication review type III of polypharmacy reduced unplanned hospitalizations in older adults: A meta-analysis of randomized clinical trials. Geriatr Gerontol Int. 2019;19(12):1275-81.

Morgado M GM. Science meets practice: towards evidence-based clinical pharmacy services. 46th ESCP symposium on clinical pharmacy; Heidelberg, Germany: International Journal of Clinical Pharmacy; 2017.

Mucherino S, Casula M, Galimberti F, Guarino I, Olmastroni E, Tragni E, et al. The Effectiveness of Interventions to Evaluate and Reduce Healthcare Costs of Potentially Inappropriate Prescriptions among the Older Adults: A Systematic Review. Int J Environ Res Public Health. 2022;19(11).

Mugunthan K, McGuire T, Glasziou P. Minimal interventions to decrease long-term use of benzodiazepines in primary care: a systematic review and meta-analysis. Br J Gen Pract. 2011;61(590):e573-8.

Narayan SW, Nishtala PS. Discontinuation of Preventive Medicines in Older People with Limited Life Expectancy: A Systematic Review. Drugs Aging. 2017;34(10):767-76.

Niznik JD, Collins BJ, Armistead LT, Larson CK, Kelley CJ, Hughes TD, et al. Pharmacist interventions to deprescribe opioids and benzodiazepines in older adults: A rapid review. Res Social Adm Pharm. 2022;18(6):2913-21.

Oktora MP, Kerr KP, Hak E, Denig P. Rates, determinants and success of implementing deprescribing in people with type 2 diabetes: A scoping review. Diabet Med. 2021;38(2):e14408.

Paquin AM, Zimmerman K, Rudolph JL. Risk versus risk: a review of benzodiazepine reduction in older adults. Expert Opin Drug Saf. 2014;13(7):919-34.

Parsons C, Lim WY, Loy C, McGuinness B, Passmore P, Ward SA, Hughes C. Withdrawal or continuation of cholinesterase inhibitors or memantine or both, in people with dementia. Cochrane Database Syst Rev. 2021;2(2):CD009081.

Pazan F, Petrovic M, Cherubini A, Cruz-Jentoft AJ, Denkinger M, van der Cammen TJM, et al. A Systematic Review of the Current Evidence from Randomised Controlled Trials on the Impact of Medication Optimisation or Pharmacological Interventions on Quantitative Measures of Cognitive Function in Geriatric Patients. Drugs Aging. 2022;39(11):863-74.

Primack BA. ACP Journal Club. Review: Minimal interventions (e.g., a letter) reduce long-term benzodiazepine use in primary care. Ann Intern Med. 2012;156(4):JC2-08.Pruskowski JA, Springer S, Thorpe CT, Klein-Fedyshin M, Handler SM. Does Deprescribing Improve Quality of Life? A Systematic Review of the Literature. Drugs Aging. 2019;36(12):1097-110.

Raae-Hansen C, O’Mahony D, Kearney PM, Sahm LJ, Cullinan S, Rutjes AWS, et al. 112Changing Behaviours: A Systematic Literature Review of Deprescribing Interventions in Older People. Age and Ageing. 2017;46(Suppl_3):iii13-iii59.

Raban MZ, Gasparini C, Li L, Baysari MT, Westbrook JI. Effectiveness of interventions targeting antibiotic use in long-term aged care facilities: a systematic review and meta-analysis. BMJ Open. 2020;10(1):e028494.

Rank MA, Hagan JB, Park MA, Podjasek JC, Samant SA, Volcheck GW, et al. The risk of asthma exacerbation after stopping low-dose inhaled corticosteroids: a systematic review and meta-analysis of randomized controlled trials. J Allergy Clin Immunol. 2013;131(3):724-9.

Rathbone A, Eghbali S, Baqir W. A systematic review and thematic synthesis of evaluations of pharmacy-led services in care homes across England. International Journal of Pharmacy Practice. 2020;28(Supplement_1):44-87.

Richter T, Meyer G, Mohler R, Kopke S. Psychosocial interventions for reducing antipsychotic medication in care home residents. Cochrane Database Syst Rev. 2012;12:CD008634.

Riordan DO, Walsh KA, Galvin R, Sinnott C, Kearney PM, Byrne S. The effect of pharmacist-led interventions in optimising prescribing in older adults in primary care: A systematic review. SAGE Open Med. 2016;4:2050312116652568.

Rodríguez-Perez A G-BJ, Alfaro-Lara ER, Toscano-Guzmán MD, Sierra-Torres MI, Villalba-Moreno AM. Deprescribing strategies in elderly patients or patients with several chronic conditions. European Journal of Hospital Pharmacy; 2014.

Romano S, Figueira D, Teixeira I, Perelman J. Deprescribing Interventions among Community-Dwelling Older Adults: A Systematic Review of Economic Evaluations. Pharmacoeconomics. 2022;40(3):269-95.

Ross J. Discontinuation of lithium augmentation in geriatric patients with unipolar depression: a systematic review. Can J Psychiatry. 2008;53(2):117-20.

Salahudeen MS, Duffull SB, Nishtala PS. Impact of anticholinergic discontinuation on cognitive outcomes in older people: a systematic review. Drugs Aging. 2014;31(3):185-92.

Seppala LJ, Kamkar N, van Poelgeest EP, Thomsen K, Daams JG, Ryg J, et al. Medication reviews and deprescribing as a single intervention in falls prevention: a systematic review and meta-analysis. Age Ageing. 2022;51(9).

Sheehan R, Hassiotis A. Reduction or discontinuation of antipsychotics for challenging behaviour in adults with intellectual disability: a systematic review. Lancet Psychiatry. 2017;4(3):238-56.

Sørensen AMS, Tarp S, Johannsen P, Lolk A, Bandak E, Pedersen H, et al. Analgesics use and withdrawal in people with dementia - a register-based Danish study and a systematic review. Dan Med J. 2019;66(12).

Tecklenborg S, Byrne C, Cahir C, Brown L, Bennett K. Interventions to Reduce Adverse Drug Event-Related Outcomes in Older Adults: A Systematic Review and Meta-analysis. Drugs Aging. 2020;37(2):91-8.

Thapa P, Lee SWH, Kc B, Dujaili JA, Mohamed Ibrahim MI, Gyawali S. Pharmacist-led intervention on chronic pain management: A systematic review and meta-analysis. Br J Clin Pharmacol. 2021;87(8):3028-42.

Tjia J, Kanaan A, Donovan J. Do antipsychotic dose reduction trials result in worsening behavior among nursing home residents with dementia: a systematic review of the literature. UMass Center for Clinical and Translational Science Research Retreat2012.

Topinkova E, Baeyens JP, Michel JP, Lang PO. Evidence-based strategies for the optimization of pharmacotherapy in older people. Drugs Aging. 2012;29(6):477-94.

Ulley J, Harrop D, Ali A, Alton S, Fowler Davis S. Deprescribing interventions and their impact on medication adherence in community-dwelling older adults with polypharmacy: a systematic review. BMC Geriatr. 2019;19(1):15.

van der Worp H, Jellema P, Hordijk I, Lisman-van Leeuwen Y, Korteschiel L, Steffens MG, Blanker MH. Discontinuation of alpha-blocker therapy in men with lower urinary tract symptoms: a systematic review and meta-analysis. BMJ Open. 2019;9(11):e030405.

Van Leeuwen E, Petrovic M, van Driel ML, De Sutter AI, Stichele RV, Declercq T, Christiaens T. Discontinuation of Long-Term Antipsychotic Drug Use for Behavioral and Psychological Symptoms in Older Adults Aged 65 Years and Older With Dementia. J Am Med Dir Assoc. 2018;19(11):1009-14.

Van Leeuwen E, van Driel ML, Horowitz MA, Kendrick T, Donald M, De Sutter AI, et al. Approaches for discontinuation versus continuation of long-term antidepressant use for depressive and anxiety disorders in adults. Cochrane Database Syst Rev. 2021;4(4):CD013495.

Vasanthamohan L, Boonyawat K, Chai-Adisaksopha C, Crowther M. Reduced-dose direct oral anticoagulants in the extended treatment of venous thromboembolism: a systematic review and meta-analysis. J Thromb Haemost. 2018;16(7):1288-95.

Velten SJ, Parsons C, Briesacher B, Gurwitz J, Tjia J. Interventions to improve prescribing at the end-of-life: A systematic review. Journal of the American Geriatrics Society. 2011;59(S1):S1-S232.

Wright A, Diebold J, Otal J, Stoneman C, Wong J, Wallace C, Duffett M. The Effect of Melatonin on Benzodiazepine Discontinuation and Sleep Quality in Adults Attempting to Discontinue Benzodiazepines: A Systematic Review and Meta-Analysis. Drugs Aging. 2015;32(12):1009-18.

Zhang X, Cao D, Yan M, Liu M. The feasibility of Chinese massage as an auxiliary way of replacing or reducing drugs in the clinical treatment of adult type 2 diabetes: A systematic review and meta-analysis. Medicine (Baltimore). 2020;99(34):e21894.

Van Leeuwen E, Petrovic M, van Driel ML, De Sutter AI, Vander Stichele R, Declercq T, Christiaens T. Withdrawal versus continuation of long-term antipsychotic drug use for behavioural and psychological symptoms in older people with dementia. Cochrane Database Syst Rev. 2018;3:CD007726.
