# Supplementary material for: Deprescribing interventions in older adults: An overview of systematic reviews
Source: PLoS One. 2024 Jun 17;19(6):e0305215. doi: 10.1371/journal.pone.0305215 (PMC11182547; doi:10.1371/journal.pone.0305215)
Supplement: S4 Table — (DOCX) [file pone.0305215.s008.docx]

S4 Table. Study characteristics of included systematic reviews

| **Author (Year)** | **# Studies (# of People)** | **Setting(s) and Population Characteristics** | **Objectives and Intervention (Aim, Harm**[**^a^**](#Harm)**, Intervention type, Facilitator of intervention)** | **Comparator** | **Outcomes**[^b^](#GRADE) | **AMSTAR 2** | **Eligible Primary Studies** | |
| --- | --- | --- | --- | --- | --- | --- | --- | --- |
|  |  |  |  |  |  |  | **# Studies (Design)** | **# of People (Mean Age**[^c^](#MeanAge)**)** |
| **Specific Medication Target** | | | | | | | | |
| ***Anticholinergics*** | | | | | | | | |
| **Nakham et al (2020) [29]** | 8 (1,061) | • Setting: not stated • Age 65+  • On long-term medication (>12 weeks) | • Aim: reduce anticholinergic burden  • Harm: not specified • Intervention type: no restrictions • Facilitator: not stated | Usual care | • Medication use (i.e., number of drugs, anticholinergic burden or other score) • Patient outcomes such as falls  • Cost outcomes | Critically low | 4 (4 RCTs) | 423  (76-85) |
| **Salahudeen et al (2022) [30]** | 23 (65,114) | • Setting: any healthcare setting • Age 65+ | • Aim: reduce anticholinergic prescribing  • Harm: not specified • Intervention type: no restrictions • Facilitator: healthcare professional | Not specified | • Anticholinergic prescribing  • Adverse drug events | Critically low | 9 (3 RCTs, 3 cRCTs, 1 parallel arm RT, 1 quasi-experimental, 1 prospective case-control) | 47,650 (71-87) |
| ***Antihyperglycemics*** | | | | | | | | |
| **Black et al (2017) [27]** | 2 (6,352) | • Setting: any  • Age 18+ • Taking antihyperglycemic medications for T2DM | • Aim: deprescribe at least one antihyperglycemic medication  • Harm: specified; not defined • Intervention type: no restrictions • Facilitator: not stated | Continuing antihyperglycemic medications | • Hypoglycemia  (GRADE: very low) • Falls • Adverse drug reactions • Frequency of blood glucose testing • Blood glucose levels  • HbA1C levels  (GRADE: very low) • Pill burden • Health care utilization  • Quality of life and patient satisfaction  • Length of stay in hospital  • Microvascular complications • Macrovascular outcomes • Polyuria, hyperglycemia, and/or sleep disturbances | Critically low | 2 (2 CBAs) | 6,352  (77 -84) |
| **Seidu et al (2019) [28]** | 10 (26,925) | • Setting: any  • Age 65+ • With T2DM | • Aim: deintensify antidiabetic medication and other therapies  • Harm: specified; not defined • Intervention type: no restrictions • Facilitator: not stated | Usual care or continuing medications | • Measures of glycaemia • Admission rates  • Hospitalizations  • Complications  • Mortality • Quality of life  • Patient satisfaction | Low | 2 (2 CBAs) | 6,352  (77-84) |
| ***Antihypertensives*** | | | | | | | | |
| **Reeve et al (2020) [31]**  Meta-analysis | 6 (1,073) | • Setting: community, residential aged care facilities or hospital. • Age 50+  • With hypertension or required primary prevention of CVD • Prescribed ≥ 1 antihypertensive medication | • Aim: withdrawal of antihypertensive medication  • Harm: specified; defined • Intervention type: medication withdrawal • Facilitator: not stated | Continuation of anti-hypertensive medication | Primary outcomes: • Mortality (GRADE: low) • Myocardial infarction (GRADE: very low) • Adverse drug reactions and adverse drug withdrawal reactions (GRADE: very low)  Secondary outcomes:  • Systolic BP (GRADE: low)  • Diastolic BP (GRADE: low)  • Hospitalization (GRADE: low)  • Stroke (GRADE: low)  • Success rate of withdrawal from antihypertensive drugs  • Quality of life  • Falls | Low | 5 (5 RCTs) | 832  (60-82) |
| ***Proton Pump Inhibitors*** | | | | | | | | |
| **Wilsdon et al (2017) [41]** | 21 (>164,180) | • Setting: hospital, community dwelling or aged care facilities. • Age 65+ • Inappropriate PPI use | • Aim: deprescribe inappropriate PPI  • Harm: not specified • Intervention type: no restrictions • Facilitator: not stated | Not required | Primary outcome:  • Inappropriate PPI use  Secondary outcomes:  • Mortality  • Hospitalization rates  • Recurrence of peptic ulcer disease  • Adverse events | Critically low | 4 (3 RCTs, 1 cohort study) | 71,306 (82-87) |
| ***Psychotropics*** | | | | | | | | |
| **Nishtala et al (2008) [38]**  Meta-analysis | 11 (13,010) | • Setting: long-term care facilities • Age 65+ | • Aim: optimize the use of psychotropic drugs  • Harm: not specified  • Intervention type: medication reviews and/or educational intervention • Facilitator: not stated | Not stated | Primary outcome: • Proportion of residents using one or more psychotropic drugs  Secondary outcomes:  • Cognitive function  • Depression  • Falls | Critically low | 5 (5 RCTs) | 7596 (65+) |
| **Parr et al (2009) [35]**  Meta-analysis | 32 (15,787) | • Setting: general practice or outpatient  • Age not specified • On BZDs ≥ 3 months | • Aim: discontinue BDZs  • Harm: not specified • Intervention type: brief interventions, gradual dose reduction and psychological interventions. • Facilitator: pharmacists, physicians | Adjunctive treatment with routine care or GDR alone | • Cessation rates | Critically low | 4 (4 RCTs) | 4,829  (62-71) |
| **Thompson Coon et al (2014) [36]** | 23 (>19,300)[^e^](#SampleSize) | • Setting: community residential care  • Age not specified  • With dementia | • Aim: reduce inappropriate prescribing of antipsychotics  • Harm: not specified • Intervention type: no restrictions • Facilitator: health professionals (e.g., GP, psychiatrists, pharmacists) | Not stated | • Change in medication use | Low | 6 (6 RCTs) | 4912 (Not stated) |
| **Reeve et al (2017) [33]** | 7 (1,214) | • Setting: community, hospital or aged care • Age 65+ • On long term BZDs and/or Z-drugs (≥ 4 weeks) | • Aim: deprescribe long-term use of BZDs and/or Z-drugs  • Harm: specified; defined • Intervention type: no restrictions • Facilitator: not stated | Not stated | • Number of participants continuing vs. stopping BZD or Z-drug use • Clinical outcomes • Adverse drug withdrawal reactions • Return of condition/ loss of benefit | Critically low | 5 (5 RCTs) | 1,023 (73-82) |
| **Hoyle et al (2018) [34]** | 14 (2,852) | • Setting: nursing homes  • No population characteristics stated  • Age not specified | • Aim: reduce antipsychotic and/or BDZ use  • Harm: not specified • Intervention type: professional and organization interventions defined by EPOC • Facilitator: not stated | Not stated | Clinical outcomes as: • Behavioral and psychological symptoms • Quality of life • Falls | Critically low | 9 (5 RCTs,  3 nRCTs, 1 cITS) | 1888 (Not stated) |
| **Sheehan et al (2018) [37]**  Meta-analysis | 27 (8,556) | • Setting: any  • Any population  • Age not specified | • Aim: optimize psychotropic medications  • Harm: not specified • Intervention type: medication review • Facilitator: not stated | Not stated | • Psychotropic drug optimization (changes in medication-related variables)  • Clinical efficacy  • Adverse drug events  • Participant-reported outcomes  • Economic | Critically low | 3 studies in 4 articles (3 RCTs) | 652  (79-85) |
| **Dou et al (2019) [32]**  Meta-analysis | 11 (1,575) | • Setting: not stated • Age 60+  • Using BDZs ≥3 months | • Aim: withdraw long-term BZDs  • Harm: not specified • Intervention type: different methods to taper BZD • Facilitator: not stated | Standard care | • Percentage completely tapered off BZD | Critically low | 7 (7 RCTs) | 1,181  (60-75) |
| **Lynch et al (2020) [39]**  Meta-analysis | 8 (2,071) | • Setting: primary care  • Age 18+  • prescribed BZRAs on a long-term basis | • Aim: evaluate brief interventions targeting long-term BZRA use  • Harm: not specified • Intervention type: brief intervention targeting long-term BZRA use behavior • Facilitator: healthcare professional | Usual care | Primary outcomes:  • BZRA use (complete dis-continuation or reduction by ≥ 25%)  Secondary outcomes:  • HRQoL  • Withdrawal symptoms  • Anxiety  • Sleep quality  • Depression  • Health-care utilization | Critically low | 7 (5 RCTs, 2 cRCTs) | 1,932 (62-75) |
| **Ribeiro et al (2021) [40]** | 11 (>150,347) | • Setting: not stated  • chronic BZD users | • Aim: identify effective approaches to promote deprescription of benzodiazepines for chronic users  • Harm: not specified • Intervention type: Combined or multidisciplinary deprescription strategies (excluding interventions focused on health care providers). • Facilitator: healthcare professional | Individual approach to healthcare | • Effectiveness in benzodiazepine deprescription | Low | 4 (1 RCT, 2 cRCTs, 1 ITS) | 148,539 (65+) |
| ***Multiple Target Medication Classes*** | | | | | | | | |
| **Iyer et al (2008) [15]** | 31 (8,972) | • Setting: any  • Age 65+ | • Aim: assess the benefits and risks of medication withdrawal, target medication[^d^](#MedTarget) not stated  • Harm: specified; not defined • Intervention type: medication withdrawal  • Facilitator: not stated | RCTs: Continuing medications  Other designs:  No restrictions | • Clinically relevant outcome (not specified) | Critically low | 4 (4 RCTs) | 448  (65+) |
| **General Deprescribing** [**^d^**](#MedTarget) | | | | | | | | |
| **Castelino et al (2009) [47]** | 12 (3,958) | • Setting: not stated • Age 65+ | • Aim: reduce suboptimal prescribing of various drugs / drug classes  • Harm: not specified • Intervention type: no restrictions • Facilitator: pharmacist | Not stated | • Suboptimal prescribing (overuse, misuse or underuse) | Critically low | 7 (7 RCTs) | 1,944 (65+) |
| **Kaur et al (2009) [58]** | 24 (1,559,623) | • Setting: not stated • Age 65+ | • Aim: improve inappropriate prescribing  • Harm: not specified  • Intervention type: no restrictions  • Facilitator: not stated | Not stated | • Inappropriate prescribing | Critically low | 8 (8 RCTs) | 14963 (65+) |
| **Verrue et al (2009) [70]** | 8 (7,299) | • Setting: nursing homes • Age 65+  • No specific disease/pathology | • Aim: improve the quality of prescribing  • Harm: not specified  • Intervention type: no restrictions • Facilitator: pharmacist | Not stated | Not specified | Critically low | 7 (6 RCTs, 1 nRCT) | 6584  (65+) |
| **Tjia et al (2013) [69]** | 36 (13,906) | • Setting: assisted living, nursing home, hospital, or hospice • Age 65+ • With an indication of frailty/disability | • Aim: discontinue unnecessary daily medications for chronic conditions  • Harm: not specified • Intervention type: no restrictions • Facilitator: not stated | Not stated | • Reductions in the number of unnecessary medications | Critically low | 17 (11 RCTs, 3 cRCT, 3 nRCT) | 9330 (65+) |
| **Clyne et al (2016) [50]** | 21 (156,529) | • Setting: community dwelling • Age 65+ | • Aim: improve PIP  • Harm: not specified • Intervention type: no restrictions • Facilitator: not stated | Usual care or alternative intervention | • Change in PIP | Critically low | 9 (3RCTs, 6 cRCTs) | 14,917 (65+) |
| **Johansson et al (2016) [56]**  Meta-analysis | 25 (10,980) | • Setting: any  • Patients with polypharmacy: ≥4 drugs or 80% of study population taking ≥4 drugs  •Age: ≥ 65 or 80% of population ≥ 65 | • Aim: reduce polypharmacy  • Harm: not specified • Intervention type: no restrictions • Facilitator: not stated | No intervention or usual care | Primary outcomes:  • Mortality (GRADE: low) • Hospitalization (GRADE: very low) • Change in number of drugs  Secondary outcomes:  • New morbidity  • Change in quality of life  • Physical and mental functioning  • Adverse drug event  • Adverse drug reaction  • Medication error  • Inappropriate medication  • Adverse event after discontinuation of medication  • User or patient satisfaction  • Adherence to medication  • Healthcare utilization  • Costs/ cost-effectiveness | Critically low | 25 (21 RCTs, 4 nRCTs) | 10,980 (70-88) |
| **Walsh et al (2016) [71]**  Meta-analysis | 5 (1651) | • Setting: hospital  • Age 65+ • Or any age with dementia | • Aim: improve quality of prescribing  • Harm: not specified • Intervention type: no restrictions • Facilitator: pharmacist | Required but not specified | • Reduction of PIP | Critically low | 5 (3 RCTs^,^  2 nRCTs) | 1,651  (82-87) |
| **Dalton et al (2018) [51]**  Meta-analysis | 8 (>48,204)[^e^](#SampleSize) | • Setting: hospital • Age 65+ | • Aim: reduce PIP  • Harm: not specified • Intervention type: computer-generated recommendation • Facilitator: not relevant | Usual care or non-exposed | Primary outcomes:  • Reductions in PIP or patients with PIP.  Secondary outcomes:  • Patient outcomes  • Recommendation acceptance rates | Critically low | 2 (1 RCTs, 1 cITS) | 5586  (75–78) |
| **Hansen et al (2018) [53]**  Meta-analysis | 25 (20,812) | • Setting: any  • Age 65+ | • Aim: examine behavior change techniques for reducing medications and inappropriate prescribing.  • Harm: not specified  • Intervention type: no restrictions • Facilitator: not stated | Active intervention or sham/no intervention | Primary outcomes:  • Number of total and inappropriate prescriptions and/or drugs • Proportion with a reduction in number of total and inappropriate prescriptions and/or drugs • Implementation of recommendations  Secondary outcome:  • Change in MAI score | Critically low | 24 (24 RCTs) | 20,812 (70-87) |
| **Kallio et al (2018) [57]** | 16 (7,340) | • Setting: outpatient • Age 65+ | • Aim: describe the outcomes of medication reviews  • Harm: not specified • Intervention type: medication reviews • Facilitator: community pharmacists | No restrictions | Not specified | Critically low | 3 (3 RCTs) | 3,143 (65+) |
| **Rankin et al (2018) [62]**  Meta-analysis | 32 (28,672) | • Setting: any  • Age 65+ • Having >1 long term medical condition • Receiving polypharmacy (≥4 prescribed medicines) | • Aim: improve appropriate polypharmacy  • Harm: specified; defined  • Intervention type: no restrictions  • Facilitator: not stated | Usual care | Primary outcomes: • Medication appropriateness (GRADE: very low) • PIMs (GRADE: very low) • Potential prescribing omissions (GRADE: low to very low) • Hospital admissions (GRADE: low)  Secondary outcomes:  • Medication-related problem  • Adherence to medication  • Quality of life (GRADE: low) | Low | 30 (23 RCTs, 2 nRCTs,  2 CBA, 3 cRCT) | >7533 (64–85) |
| **Thillainadesan et al (2018) [67]** | 9 (2,522) | • Setting: hospital • Age 65+ (median) | • Aim: reduce PIM  • Harm: not specified • Intervention type: no restrictions  • Facilitator: pharmacist, physician, and prescriber | Usual care | Primary outcome:  • Reduction in PIMs  Secondary outcomes:  • Mortality  • Falls  • Cognitive function  • Adverse drug withdrawal events  • Quality of life  • Hospitalizations | Critically low | 9 (9 RCTs) | 2,522  (75–87) |
| **Thio et al (2018) [68]** | 26 (8,773) | • Setting: primary care or nursing home • Age 18+ • On long-term medication (>4 wks) | • Aim: withdraw ≥ 1 long-term medications  • Harm: specified; defined  • Intervention type: no restrictions • Facilitator: not stated | Continuing medication | • Number of patients who successfully stopped medication.  • Patients who experienced relapse of symptoms or restarted medication • Early dropout of the study | Critically low | 23 (23 RCTs) | 8,146  (62-89) |
| **Monteiro et al (2019) [61]** | 16 (>266,562) | • Setting: any  • Age 65+ | • Aim: reduce PIPs/PIMs  • Harm: not specified • Intervention type: computerized decision support tools • Facilitator: not relevant | No restrictions | • Outcomes categorized as PIP or PIM-related. • Number of prescriptions • Adverse drug reactions • Potential drug-drug interactions | Critically low | 4 (4 RCTs) | 94942 (75-82) |
| **Shrestha et al (2020) [13]** | 9 (1,375) | • Setting: any  • Age 65+ • With life-limiting illness and limited life expectancy up to 2 years | • Aim: deprescribe inappropriate medications  • Harm: not specified • Intervention type: no restrictions • Facilitator: healthcare professional | Usual care or any head-head intervention | • Medication appropriateness • Clinical measures • Mortality  • Quality of life • Falls • Sleep quality • Bowel function • Cognitive function • Performance status and  • Symptoms status  • Costs | Critically low | 4 (3 RCTs, 1 nRCT) | 972  (74-84) |
| **Almutairi et al (2020) [43]**  Meta-analysis | 25 (22,989) | • Setting: aged care facility, nursing homes, residential continuing care hospitals • Age 60+ | • Aim: increase the appropriateness of medications used  • Harm: not specified • Intervention type: no restrictions. • Facilitator: healthcare professional | Not stated | • Medication appropriateness  • Residents’ clinical outcomes (not specified) | Critically low | 14 (6 RCTs, 8 cRCTs) | 9921 (65+) |
| **Bloomfield et al (2020) [45]**  Meta-analysis | 38 trials reported in 47 papers (419,902) | • Setting: community-dwelling  • Age 65+ | • Aim: deprescribe medications  • Harm: specified; not defined • Intervention type: comprehensive medication review, education and feedback, or computerized decision support • Facilitator: not stated | Not stated | Primary outcomes:  • Quality of life (GRADE: low) • Mortality (GRADE: low) • Hospitalizations (GRADE: moderate) • Falls (GRADE: low) • Adverse drug withdrawal events • Major adverse cardiac events • Delirium • PIMs | Critically low | 24 (12 RCTs, 12 cRCT) | 103873 (70-83) |
| **Earl et al (2020) [52]** | 27  (>89,047)[^e^](#SampleSize) | • Setting: Not stated • Older adults (no specific age defined) | • Aim: deprescribe to reduce polypharmacy and use of STOPP interventions to reduce PIMs  • Harm: not specified • Intervention type: no restrictions • Facilitator: not stated | Not stated | • Reduction of preventable adverse drug events • Polypharmacy • PIMs  • Other relevant outcomes (not specified) | Critically low | 10 (5 RCTs, 4 cRCTs, 1 feasibility nRCT) | 84,424 (65+) |
| **Hart et al (2020) [54]** | 14 (8,300) | • Setting: not stated • Age 60+ • History of fall-related injury/fall. | • Aim: reduce or discontinue FRIDs  • Harm: not specified • Intervention type: no restrictions • Facilitator: not stated | Not stated | • FRID use prior to and following  fall-related healthcare episode  • Falls • FRID use | Critically low | 3 (3 RCTs) | 997  (76-85) |
| **Abu Fadaleh et al (2021) [42]**  Meta-analysis | 18 (6200) | • Setting: community • Age 65+ • Community-dwelling older adults capable of self-care activities | • Aim: determine the value of home medication review on health care utilization  • Harm: not specified • Intervention type: home medication review • Facilitator: healthcare professional | Usual care with no medication review | Primary outcomes: • Health care utilization (hospitalization or ED admission)  Secondary outcomes:  • Mortality  • Quality of life  • Economic outcomes  • Medication outcomes (adverse events, complexity of regimen, change in the number of medications, and medication appropriateness (e.g. Beers Criteria, STOPP, MAI) | Critically low | 9 (6 RCTs, 2 nRCT, 1 cRCT) | 2974  (65+) |
| **Alshammari et al (2021) [44]** | 27 (16,093) | • Setting: hospital • Age 65+ | • Aim: investigate clinical and non-clinical outcomes of PIM reviews  • Harm: not specified  • Intervention type: PIM review using explicit criteria • Facilitator: healthcare professional | Not stated | Primary outcome: • PIM reduction  Secondary outcomes:  • Clinical and non-clinical outcomes | Critically low | 7 (5 RCTs , 1 nRCT, 1 ITS) | 10,230 (65+) |
| **Cardona et al (2021) [46]** | 7 (1,747) | • Setting: hospital • Age not specified  • Older patients near the end of life (terminal, palliative, or limited life expectancy) | • Aim: ascertain effectiveness and post-discharge sustainability of hospital-initiated deprescribing strategies  • Harm: not specified  • Intervention type: Interventions intended to reduce the number or dose of inappropriate medications to any extent.  • Facilitator: hospital staff | Different intervention or usual care (as defined by authors), or modified usual care, or no intervention at all | Primary outcome: • Deprescribing measures (mean number/proportion of PIM that remained discontinued post-discharge; proportions of  patients maintaining discontinuation or dose reduction of at least one medication for at ≥ 6 months)  Secondary outcomes:  • Deprescribing measures <6 months  • MAI  • Hospital admissions or ED visits  • Number/rates of medication-related or all-cause adverse events | High | 4 (4 RCTs) | 834  (83-86) |
| **Christopher et al (2021) [48]**  Meta-analysis | 13 (6,173) | • Setting: Community pharmacy, patient’s home or medical center clinics or home care unit  • Age 65+ | • Aim: To determine the clinical and humanistic outcomes of community pharmacy-based interventions  • Harm: not specified  • Intervention type: Community pharmacy-based interventions  • Facilitator: community pharmacists | Non-intervention or not receiving community pharmacy-based services | Humanistic and clinical outcomes  • Hospitalization (GRADE: moderate)  • Falls (GRADE: high)  • BZD discontinuation (GRADE: high) | Critically low | 5 (3 cRCTs, 2 RCTs) | 1490  (65+) |
| **Ibrahim et al (2021) [55]** | 6 (657) | • Setting: Any  • Age 65+  • Living with frailty identified by reliable measures. | • Aim: explore the safety and impact of deprescribing among older people living with frailty identified by reliable measures.  • Harm: specified, defined  • Intervention type: deprescribing as the only intervention or as part of medication review intervention where deprescribing accounts for ≥ 50% of total recommendations  • Facilitator: not specified | Any, no, or comparator considered | Primary outcome: • Safety (adverse events, hospital admission, all-cause mortality)  Secondary outcomes:  • Clinical outcomes (i.e., frailty status, function, falls, cognition,  depression, quality of life)  • Medication-related outcomes (changes in number of medications and PIMs)  • Feasibility of deprescribing (i.e. number of patients/proportion who successfully stopped medications)  • Acceptability  • Cost-related outcomes. | Low | 4 (2 RCTs, 2 nRCTs) | 608 (79-85) |
| **Laberge et al (2021) [59]** | 11 (6,375) | • Setting: Any  • 80% aged 65+  • multimorbidity (2+ conditions) and having polypharmacy | • Aim: examine economic impact of medication optimization interventions  • Harm: not specified • Intervention type: no restrictions • Facilitator: not specified | Not receiving intervention | Primary outcome: • Cost-effectiveness, cost-utility, or cost-benefit  Secondary outcomes:  • Clinical outcomes | Critically low | 5 (1 RCT, 4 cRCTs) | 3,080 (not stated) |
| **Shrestha et al (2021) [65]**  Meta-analysis | 5 (689) | • Setting: any  • Age 65+  • Near end of life due to any life limiting illness, prescribed ≥1 potentially inappropriate dual purpose medications | • Aim: deprescribe potentially inappropriate dual purpose medications  • Harm: not specified • Intervention type: no restrictions • Facilitator: not specified | Usual or standard care | • Any clinical or patient-reported outcomes such as QoL, short-term mortality, falls, hospital referrals or admissions, ED visits, fractures, or medication-related adverse events | Critically low | 5 (4 RCTs,  1 quasi-  experimental) | 689  (82-86) |
| **Lee et al (2022) [60]** | 16 (3,745) | • Setting: surgery-related settings (clinic and hospital)  • Age 65+  • Undergoing planned or emergency surgery | • Aim: deprescribe during preoperative period  • Harm: not specified • Intervention type: deprescribing or medication-related interventions • Facilitator: geriatrician, pharmacist, interdisciplinary team | Not stated | • not specified | Critically low | 2 (2 RCTs) | 462  (79-83) |
| **Saeed et al (2022) [64]** | 3 (1,110) | • Secondary or acute care settings  • Age 65+  • Frail older inpatients (using any tool) | • Aim: optimize medications of frail older adults  • Harm: not specified  • Intervention type: no restrictions but multicomponent interventions were excluded. • Facilitator: healthcare professional | Usual care or no intervention | • Change in medication (dose, frequency, dosage form, number of medications stopped or started)  • Appropriateness of prescribing  • Adverse drug reactions  • Death  • Quality of life  • Falls or recurrent falls  • Fractures  • Disability  • Cost of medication and health care utilisation (i.e. hospital readmission and duration of hospitalization) | Critically low | 3 (3 RCTs) | 1,110 (75+) |
| **Rodrigues et al (2022) [63]** | 47 (not specified) | • Setting: not stated  • Age 65+ | • Aim: reduce PIM prescriptions  • Harm: not specified • Intervention type: Intervention aiming to optimize pharmacotherapy • Facilitator: no restrictions | Not stated | • Change rate between the mean number of PIM per patient and/or the mean number of patients with PIM before and after an intervention | Critically low | 16 (5 RCTs, 7 cRCTs, 1 block RCT, 2 nRCTs, 1 ITS) | 88,824 (65-86) |
| **Stotzner et al (2022) [66]** | 58 (30,554) | • Setting: Inpatient and outpatient psychiatric settings and nursing homes • Adults 18+ | • Aim: optimize polypharmacy for patients with psychiatric disorders  • Harm: not specified • Intervention type: any intervention using a clear tool, guideline, or protocol for targeted optimization of polypharmacy • Facilitator: no restrictions | Not stated | • Any clinical or medication-related outcome | Critically low | 14  (14 RCTs) | 8,909 (77-88) |
| **Clarkson et al (2023) [49]** | 19 (10,914) | • Setting: medical specialist outpatient clinics  • Age 60+ | • Aim: review the feasibility and effectiveness of deprescribing interventions implemented within specialist outpatient clinics.  • Harm: not specified • Intervention type: no restrictions • Facilitator: healthcare professional | Not stated | Primary outcome: • Change in medication load (total number or PIM count)  Secondary outcomes:  • Maintenance of deprescription  • Clinical benefits | Critically low | 7 (3 RCTs, 2 cRCTs, 2 pilot RCTs) | 7,986 (69- 82) |
| **Specific Medication Targets and General Deprescribing** | | | | | | | | |
| **Page et al (2016) [12]**  Meta-analysis | 116 (34,143) | • Setting: any  • Age 65+ • Prescribed ≥1 regular medication | • Aim: deprescribe ≥ 1 regular prescription medications  • Harm: specified; defined • Intervention type: no restrictions • Facilitator: healthcare professional | Any comparator or usual care | Primary outcome: • Mortality Secondary outcomes:  • Adverse drug withdrawal events  • Psychological and physical health  • Quality of life  • Medication usage | Critically low | 55 (45 CTs, 5 nRCTs, 5 cohort studies) | 14,268 (65-86) |

Information on setting and population characteristics, objectives and intervention, comparison and outcomes are based on what was specified in the methods of each review. The two rightmost columns of the table summarize information specific to eligible primary studies within each review.

*Abbreviations:* BZD, benzodiazepines; CBA, controlled-before-after studies; CDS, computerized decision support; CVD, cardiovascular disease; EPOC, Cochrane Effective Practice and Organization of Care; FRID, fall risk increasing drugs; HbA1c, glycated hemoglobin; cITS, interrupted time series; ED, emergency department; MAI, medication appropriateness index; nRCT, non-randomized controlled trials; PIMs, potentially inappropriate medications; PIPs, potentially inappropriate prescriptions; PPI, proton pump inhibitor; RCT, randomized controlled trials; STOPP, Screening Tool of Older Persons' Prescriptions; T2DM, Type 2 Diabetes mellitus; Z-drugs, nonbenzodiazepine drugs.

^a^ This identifies whether studies explicitly specified an objective to examine harms associated with interventions.

^b^ GRADE results in parentheses represent GRADE recommendations, by outcome, as reported by reviews if performed.

^c^ Represented in range of mean ages of participants in eligible primary studies when available, if reported by systematic reviews.

^d^ Studies that focused on non-medication specific deprescribing, polypharmacy, PIM, medication appropriateness.

^e^ Indicates that one or more primary studies did not report their sample size.

**References**

(numbering matches the manuscript, only sources to S4 Table included)

12. Page AT, Clifford RM, Potter K, Schwartz D, Etherton-Beer CD. The feasibility and effect of deprescribing in older adults on mortality and health: A systematic review and meta-analysis. Br J Clin Pharmacol. 2016;82(3):583-623.

13. Shrestha S, Poudel A, Steadman K, Nissen L. Outcomes of deprescribing interventions in older patients with life-limiting illness and limited life expectancy: A systematic review. Br J Clin Pharmacol. 2020;86(10):1931-45.

15. Iyer S, Naganathan V, McLachlan AJ, Le Couteur DG. Medication withdrawal trials in people aged 65 years and older: A systematic review. Drugs Aging. 2008;25(12):1021-31.

27. Black CD, Thompson W, Welch V, McCarthy L, Rojas-Fernandez C, Lochnan H, et al. Lack of evidence to guide deprescribing of antihyperglycemics: A systematic review. Diabetes Ther. 2017;8(1):23-31.

28. Seidu S, Kunutsor SK, Topsever P, Hambling CE, Cos FX, Khunti K. Deintensification in older patients with type 2 diabetes: A systematic review of approaches, rates and outcomes. Diabetes Obes Metab. 2019;21(7):1668-79.

29. Nakham A, Myint PK, Bond CM, Newlands R, Loke YK, Cruickshank M. Interventions to reduce anticholinergic burden in adults aged 65 and older: A systematic review. J Am Med Dir Assoc. 2020;21(2):172-80.e5.

30. Salahudeen MS, Alfahmi A, Farooq A, Akhtar M, Ajaz S, Alotaibi S, et al. Effectiveness of interventions to improve the anticholinergic prescribing practice in older adults: A systematic review. J Clin Med. 2022;11(3).

31. Reeve E, Jordan V, Thompson W, Sawan M, Todd A, Gammie TM, et al. Withdrawal of antihypertensive drugs in older people. Cochrane Database Syst Rev. 2020;6(6):CD012572.

32. Dou C, Rebane J, Bardal S. Interventions to improve benzodiazepine tapering success in the elderly: A systematic review. Aging Ment Health. 2019;23(4):411-6.

33. Reeve E, Ong M, Wu A, Jansen J, Petrovic M, Gnjidic D. A systematic review of interventions to deprescribe benzodiazepines and other hypnotics among older people. Eur J Clin Pharmacol. 2017;73(8):927-35.

34. Hoyle DJ, Bindoff IK, Clinnick LM, Peterson GM, Westbury JL. Clinical and economic outcomes of interventions to reduce antipsychotic and benzodiazepine use within nursing homes: A systematic review. Drugs Aging. 2018;35(2):123-34.

35. Parr JM, Kavanagh DJ, Cahill L, Mitchell G, McD Young R. Effectiveness of current treatment approaches for benzodiazepine discontinuation: A meta-analysis. Addiction. 2009;104(1):13-24.

36. Thompson-Coon J, Abbott R, Rogers M, Whear R, Pearson S, Lang I, et al. Interventions to reduce inappropriate prescribing of antipsychotic medications in people with dementia resident in care homes: A systematic review. J Am Med Dir Assoc. 2014;15(10):706-18.

37. Sheehan R, Strydom A, Brown E, Marston L, Hassiotis A. Association of focused medication review with optimization of psychotropic drug prescribing: A systematic review and meta-analysis. JAMA Netw Open. 2018;1(6):[e183750.

38. Nishtala PS, McLachlan AJ, Bell JS, Chen TF. Psychotropic prescribing in long-term care facilities: Impact of medication reviews and educational interventions. Am J Geriatr Psychiatry. 2008;16(8):621-32.

39. Lynch T, Ryan C, Hughes CM, Presseau J, van Allen ZM, Bradley CP, Cadogan CA. Brief interventions targeting long-term benzodiazepine and z-drug use in primary care: A systematic review and meta-analysis. Addiction. 2020;115(9):1618-39.

40. Ribeiro PRS, Schlindwein AD. Benzodiazepine deprescription strategies in chronic users: A systematic review. Fam Pract. 2021;38(5):684-93.

41. Wilsdon TD, Hendrix I, Thynne TR, Mangoni AA. Effectiveness of interventions to deprescribe inappropriate proton pump inhibitors in older adults. Drugs Aging. 2017;34(4):265-87.

42. Abu Fadaleh SM, Charrois TL, Makhinova T, Eurich DT, Sholeh R, Sadowski CA. The effect of home medication review in community-dwelling older adults: A systematic review. J Public Health (Berl). 2022;30:1857–72.

43. Almutairi H, Stafford A, Etherton-Beer C, Flicker L. Optimisation of medications used in residential aged care facilities: A systematic review and meta-analysis of randomised controlled trials. BMC Geriatr. 2020;20(1):236.

44. Alshammari H, Al-Saeed E, Ahmed Z, Aslanpour Z. Reviewing potentially inappropriate medication in hospitalized patients over 65 using explicit criteria: A systematic literature review. Drug Healthc Patient Saf. 2021;13:183-210.

45. Bloomfield HE, Greer N, Linsky AM, Bolduc J, Naidl T, Vardeny O, et al. Deprescribing for community-dwelling older adults: A systematic review and meta-analysis. J Gen Intern Med. 2020;35(11):3323-32.

46. Cardona M, Stehlik P, Fawzy P, Byambasuren O, Anderson J, Clark J, et al. Effectiveness and sustainability of deprescribing for hospitalized older patients near end of life: A systematic review. Expert Opin Drug Saf. 2021;20(1):81-91.

47. Castelino RL, Bajorek BV, Chen TF. Targeting suboptimal prescribing in the elderly: A review of the impact of pharmacy services. Ann Pharmacother. 2009;43(6):1096-106.

48. Christopher CM, Kc B, Blebil A, Alex D, Ibrahim MIM, Ismail N, Alrasheedy AA. Clinical and humanistic outcomes of community pharmacy-based healthcare interventions regarding medication use in older adults: A systematic review and meta-analysis. Healthcare (Basel). 2021;9(11):1577.

49. Clarkson L, Hart L, Lam AK, Khoo TK. Reducing inappropriate polypharmacy for older patients at specialist outpatient clinics: A systematic review. Curr Med Res Opin. 2023;39(4):545-54.

50. Clyne B, Fitzgerald C, Quinlan A, Hardy C, Galvin R, Fahey T, Smith SM. Interventions to address potentially inappropriate prescribing in community-dwelling older adults: A systematic review of randomized controlled trials. J Am Geriatr Soc. 2016;64(6):1210-22.

51. Dalton K, O'Brien G, O'Mahony D, Byrne S. Computerised interventions designed to reduce potentially inappropriate prescribing in hospitalised older adults: A systematic review and meta-analysis. Age Ageing. 2018;47(5):670-8.

52. Earl TR, Katapodis ND, Schneiderman SR, Shoemaker-Hunt SJ. Using deprescribing practices and the screening tool of older persons' potentially inappropriate prescriptions criteria to reduce harm and preventable adverse drug events in older adults. J Patient Saf. 2020;16(3S Suppl 1):S23-S35.

53. Hansen CR, O'Mahony D, Kearney PM, Sahm LJ, Cullinan S, Huibers CJA, et al. Identification of behaviour change techniques in deprescribing interventions: A systematic review and meta-analysis. Br J Clin Pharmacol. 2018;84(12):2716-28.

54. Hart LA, Phelan EA, Yi JY, Marcum ZA, Gray SL. Use of fall risk-increasing drugs around a fall-related injury in older adults: A systematic review. J Am Geriatr Soc. 2020;68(6):1334-43.

55. Ibrahim K, Cox NJ, Stevenson JM, Lim S, Fraser SDS, Roberts HC. A systematic review of the evidence for deprescribing interventions among older people living with frailty. BMC Geriatr. 2021;21(1):258.

56. Johansson T, Abuzahra ME, Keller S, Mann E, Faller B, Sommerauer C, et al. Impact of strategies to reduce polypharmacy on clinically relevant endpoints: A systematic review and meta-analysis. Br J Clin Pharmacol. 2016;82(2):532-48.

57. Kallio SE, Kiiski A, Airaksinen MSA, Mantyla AT, Kumpusalo-Vauhkonen AEJ, Jarvensivu TP, Pohjanoksa-Mantyla MK. Community pharmacists' contribution to medication reviews for older adults: A systematic review. J Am Geriatr Soc. 2018;66(8):1613-20.

58. Kaur S, Mitchell G, Vitetta L, Roberts MS. Interventions that can reduce inappropriate prescribing in the elderly: A systematic review. Drugs Aging. 2009;26(12):1013-28.

59. Laberge M, Sirois C, Lunghi C, Gaudreault M, Nakamura Y, Bolduc C, Laroche ML. Economic evaluations of interventions to optimize medication use in older adults with polypharmacy and multimorbidity: A systematic review. Clin Interv Aging. 2021;16:767-79.

60. Lee JW, Li M, Boyd CM, Green AR, Szanton SL. Preoperative deprescribing for medical optimization of older adults undergoing surgery: A systematic review. J Am Med Dir Assoc. 2022;23(4):528-36 e2.

61. Monteiro L, Maricoto T, Solha I, Ribeiro-Vaz I, Martins C, Monteiro-Soares M. Reducing potentially inappropriate prescriptions for older patients using computerized decision support tools: Systematic review. J Med Internet Res. 2019;21(11):e15385.

62. Rankin A, Cadogan CA, Patterson SM, Kerse N, Cardwell CR, Bradley MC, et al. Interventions to improve the appropriate use of polypharmacy for older people. Cochrane Database Syst Rev. 2018;9:CD008165.

63. Rodrigues DA, Placido AI, Mateos-Campos R, Figueiras A, Herdeiro MT, Roque F. Effectiveness of interventions to reduce potentially inappropriate medication in older patients: A systematic review. Front Pharmacol. 2022;12:777655.

64. Saeed D, Carter G, Parsons C. Interventions to improve medicines optimisation in frail older patients in secondary and acute care settings: A systematic review of randomised controlled trials and non-randomised studies. Int J Clin Pharm. 2022;44(1):15-26.

65. Shrestha S, Poudel A, Cardona M, Steadman KJ, Nissen LM. Impact of deprescribing dual-purpose medications on patient-related outcomes for older adults near end-of-life: A systematic review and meta-analysis. Ther Adv Drug Saf. 2021;12:20420986211052343.

66. Stotzner P, Ferrebus Abate RE, Henssler J, Seethaler M, Just SA, Brandl EJ. Structured interventions to optimize polypharmacy in psychiatric treatment and nursing homes: A systematic review. J Clin Psychopharmacol. 2022;42(2):169-87.

67. Thillainadesan J, Gnjidic D, Green S, Hilmer SN. Impact of deprescribing interventions in older hospitalised patients on prescribing and clinical outcomes: A systematic review of randomised trials. Drugs Aging. 2018;35(4):303-19.

68. Thio SL, Nam J, van Driel ML, Dirven T, Blom JW. Effects of discontinuation of chronic medication in primary care: A systematic review of deprescribing trials. Br J Gen Pract. 2018;68(675):e663-e72.

69. Tjia J, Velten SJ, Parsons C, Valluri S, Briesacher BA. Studies to reduce unnecessary medication use in frail older adults: A systematic review. Drugs Aging. 2013;30(5):285-307.

70. Verrue CL, Petrovic M, Mehuys E, Remon JP, Vander Stichele R. Pharmacists' interventions for optimization of medication use in nursing homes : A systematic review. Drugs Aging. 2009;26(1):37-49.

71. Walsh KA, O'Riordan D, Kearney PM, Timmons S, Byrne S. Improving the appropriateness of prescribing in older patients: A systematic review and meta-analysis of pharmacists' interventions in secondary care. Age Ageing. 2016;45(2):201-9.
