# Supplementary material for: Deprescribing interventions in older adults: An overview of systematic reviews
Source: PLoS One. 2024 Jun 17;19(6):e0305215. doi: 10.1371/journal.pone.0305215 (PMC11182547; doi:10.1371/journal.pone.0305215)
Supplement: S5 Table — (DOCX) [file pone.0305215.s009.docx]

S5 Table. Summary of authors’ conclusions from systematic reviews and results of meta-analyses (Step 1)

| **Author (Year)** | **Conclusions of Systematic Reviews** | **Results of Meta-analysis [Statistic (95% Confidence Interval)]** |
| --- | --- | --- |
| **Specific Medication Targets** | | |
| ***Anticholinergics*** | | |
| Nakham et al. (2020) [29] | - Inconclusive evidence for reducing anticholinergic burden. |  |
| Salahudeen et al (2022) [30] | - Healthcare practitioner-oriented interventions were mostly effective in reducing the burden of anticholinergic medications and facilitating the discontinuation of anticholinergic medications in older people. |  |
| ***Antihyperglycemics*** | | |
| Black et al. (2017) [27] | - Limited evidence for deprescribing antihyperglycemic medications. |  |
| Seidu et al. (2019) [28] | - Majority of studies reported no deterioration in HbA1c levels, hypoglycemic episodes, falls or hospitalizations. - No significant differences observed among comparison groups for adverse events and mortality. |  |
| ***Antihypertensives*** | | |
| Reeve et al. (2020) [31] | - No significant increase in mortality, CVD or stroke, however there is low or very low certainty in these results. - There is an absence of data on falls and quality of life. | ***No effect***  Outcome: Mortality   - All-cause mortality (n=4), OR 2.08 (0.79, 5.46) - Cardiovascular mortality (n=1), OR 2.04 (0.21, 20.19)   Outcome: Clinical or patient-centered outcomes   - Myocardial infarction (fatal and non-fatal) (n=2), OR 1.86 (0.19, 17.98) - Stroke (fatal + nonfatal + TIA) (n=3), OR 1.44 (0.25, 8.35) - Quality of life (n=1), OR -0.10 (-0.35, 0.15)   Outcome: Hospitalization   - Hospitalization (n=1), OR 0.83 (0.33, 2.10)   ***Favors comparison***  Outcome: Surrogate biomarkers outcomes   - Systolic blood pressure (n=5), MD 9.75 mmHg higher (7.33, 12.18)   Subgroup analysis- Duration   - Less than 12 months (n=3), MD 9.17 mmHg (6.62, 11.71) - 12 months or longer (n=2), MD 15.30 mmHg (7.48, 23.12)   Subgroup analysis- Drug type   - Diuretics (n=4), MD 10.85 mmHg (7.92, 13.78) - Other (n=1), MD 7.40 mmHg (3.10, 11.70) - Diastolic blood pressure (n=5), MD 3.50 mmHg higher (1.82, 5.18)   Subgroup analysis-Duration   - Less than 12 months (n=3), MD 2.68 mmHg (0.87, 4.49) - 12 months or longer (n=2), MD 8.70 mmHg (4.15, 13.25)   Subgroup analysis- Drug type   - Diuretics (n=4), MD 4.42 mmHg (2.03, 6.81) - Other (n=1), MD 2.60 mmHg (0.24, 4.96)   Outcome: Restart medication   - Restart due to hypertension or other clinical reasons (n=3), OR 3.23 (1.86, 5.61) |
| ***Proton Pump Inhibitors*** | | |
| Wilsdon et al. (2017) [41] | - Some interventions more effective than others at deprescribing inappropriate medicines. |  |
| ***Psychotropics*** | | |
| Nishtala et al. (2008) [38] | - Significant positive effect on reducing psychotropic prescribing or use. | ***Favors interventions***  Outcome: Medication use   - Proportion of residents on hypnotic drugs (n=5), OR 0.576 (0.419, 0.793)   ***No effect***   - Proportion of residents on antipsychotic drugs (n=5, OR 0.813 (0.635, 1.039) |
| Parr et al. (2009) [35] | - All interventions studied were better than routine care in reducing benzodiazepines. - Relatively weak evidence for substitutive pharmacotherapy, compared to brief intervention and/or psychological intervention. | ***Favors interventions***  Outcome: Reduction in medication use: (proportion ceased BZDs by intervention)   - Brief intervention vs routine care with individual random allocation (n=3), OR 4.37 (2.28, 8.40) - Brief intervention vs routine care with practices random allocation (n=2), OR 2.21 (1.92, 2.55) - Psychological intervention vs routine care (n=3), OR 3.38 (1.86, 6.12) - Psychological intervention and gradual dose reduction (GDR) vs GDR alone at post-cessation (n=7), OR 1.82 (1.25, 2.67) or follow-up (n=6), OR 1.88 (1.19, 2.97)   ***No effect***  Outcome: Reduction in medication use   - Abrupt substitution of substitutive pharmacotherapy vs abrupt reduction alone (n=3), OR 1.69 (0.60, 4.74) - Substitutive pharmacotherapy and GDR vs GDR alone at post-cessation (n=14), OR 1.30 (0.97, 1.73) and at follow-up (n=5), OR 1.30 (0.77, 2.20)   ***Favors comparison***  Outcome: Reduction in medication use   - Abrupt substitution of substitutive pharmacotherapy vs GDR alone (n=3), OR 0.30 (0.14, 0.64) |
| Thompson Coon et al. (2014) [36] | - May be effective in short-term reducing inappropriate prescribing of antipsychotics. |  |
| Reeve et al. (2017) [33] | - Most studies found no difference in sleep quality and no significant adverse drug withdrawal reactions when tapering or substitution was used. - Withdrawal success variable, benefits and sustainability unclear. - Limited evidence available on clinical outcomes. |  |
| Hoyle et al. (2018) [34] | - Little evidence for deleterious effects from interventions to reduce antipsychotic or benzodiazepine medications - Clinical and economic outcomes remain underreported. |  |
| Sheehan et al. (2018) [37] | - Potential to contribute to psychotropic medication optimization - No evidence suggesting improved clinical and patient important outcomes. | ***Favors interventions***  Outcome: Reduction in medication use   - Reduction in psychotropic medications in cognitively impaired older adults in nursing homes (n=3), OR 0.24 (0.14, 0.39) |
| Dou et al. (2019) [32] | - Evidence of success discontinuing benzodiazepines. | ***Favors interventions***  Outcome: Reduction in medication use  Percentage of participants completely tapered off benzodiazepines   - CBT intervention studies (n=3), OR 1.93 (1.13, 3.29) - Education intervention studies (n=3), OR 5.94 (3.99, 8.83)   ***No effect***  Outcome: Reduction in medication use  Percentage of participants completely tapered off benzodiazepines   - Melatonin substitution during BZD taper studies (n=3), OR 1.16 (0.63, 2.14) |
| Lynch et al (2020) [39] | - Brief interventions delivered in primary care were more effective than usual care in reducing and discontinuing long-term BZRA use. | ***Favors interventions***  Outcome: Medication discontinuation   - Discontinued BZRA use 6 months post-intervention (n=8), RR 2.73 (1.84, 4.06) - Discontinued BZRA use 12 months post-intervention (n=2), RR 3.41 (2.22, 5.25) - Reduced BZRA use 6 months post-intervention (n=5), RR 1.68 (1.03, 2.75) |
| Ribeiro et al (2021) [40] | - Interventions aiming to promote benzodiazepine deprescription based on patient self-awareness presented good discontinuation rates. |  |
| ***Multiple Medication Classes*** | | |
| Iyer et al. (2008) [15] | - Medications may be withdrawn without causing harm. Symptoms that recurred after medication withdrawal could be easily treated by recommencing the medications. - Some evidence for specific psychotropic medicine class withdrawal in some people. |  |
| **General Deprescribing^1^** | | |
| Castelino et al. (2009) [47] | - Promising results for prescribing optimisation. |  |
| Kaur et al. (2009) [58] | - Mixed effect on reducing inappropriate prescribing. |  |
| Verrue et al. (2009) [70] | - Mixed evidence for effectiveness on pharmacotherapy in the nursing home setting. |  |
| Tjia et al. (2013) [69] | - Few rigorous intervention studies on reducing medications in frail older adults. - Differing effectiveness associated with differences in interventions, though conclusions limited by heterogeneous study design. |  |
| Clyne et al. (2016) [50] | - Some interventions appear beneficial in reducing inappropriate prescribing, though beneficial effect was small. |  |
| Johansson et al. (2016) [56] | - No convincing evidence that the strategies assessed are effective in reducing polypharmacy or have an impact on clinically relevant endpoints. - Strategies to reduce polypharmacy had no effect on all-cause mortality. | ***No effect***  Outcome: Mortality   - All pooled studies (n=18), OR 1.02 (0.84, 1.23) - Only RCTs (n=16), OR 1.05 (0.85, 1.29)   Subgroup analysis   - Studies with short follow-up 2-6 months (n=8), OR 1.13 (0.86, 1.50) - Studies with long follow-up 12-18 months (n=10), OR 0.93 (0.69, 1.24) |
| Walsh et al. (2016) [71] | - May have improvements in prescribing appropriateness in certain contexts. | ***Favors interventions***  Outcome: Medication appropriateness   - Summated MAI scores at discharge (n=4), MD −5.27 (−8.44, −2.11)   Sensitivity analysis Summated MAI scores at discharge including study at high risk of bias (n=5), MD -5.91 (-8.95, -2.87)   - Change in summated MAI scores from admission to discharge (n=4), MD −7.45 (-11.14, −3.76) |
| Dalton et al. (2018) [51] | - Can be effective in reducing potentially inappropriate prescribing. | ***Favors interventions***  Outcome: Potentially inappropriate medications   - Reduction in the proportion of patients prescribed PIMs post-intervention (n=3), OR 0.60 (0.38, 0.93) |
| Hansen et al. (2018) [53] | - Effective in reducing number of drugs taken and improving inappropriate prescribing. | ***Favors interventions***  Outcome: Medication use   - Mean number of drugs (n=8), MD -0.96 (-1.53, -0.38) - Change in number of drugs per patient (n=11), MD -0.74 (-1.26, -0.22)   Subgroup analysis-Setting   - Outpatient setting (n=9), MD -0.80 (-1.40, -0.21) - Hospital setting (n=2), MD-0.50 (-1.36, 0.37) – **No effect**   Subgroup analysis -Intervention type   - Patient-centred interventions (n=4), MD -1.01 (-2.00, -0.03) - Healthcare professional-centred interventions (n=7), MD -0.51 (-0.80, -0.22)   Subgroup analysis -RoB assessment   - Low RoB random sequence (n=8), MD -0.73 (-1.50, 0.03) – **No effect** - High RoB allocation concealment (n=3), MD -0.74 (-1.14, -0.34)   Outcome: Prescribing appropriateness   - Mean difference in change in MAI score per person (n=5), MD -5.04 (-7.40, -2.68) - No. of inappropriate drugs (n=7), OR 0.59 (0.38, 0.91)   Subgroup analysis- RoB assessment   - Low RoB allocation concealment (n=3), OR 0.48 (0.20, 1.16) – **No effect** - High RoB allocation concealment (n=4), OR 0.67 (0.45, 1.01) – **No effect**   ***No effect***  Outcome: Prescribing appropriateness   - Mean difference in number of inappropriate drugs (n=3), MD -0.19 (-0.40, 0.02) |
| Kallio et al. (2018) [57] | - Seem to reduce drug related problems and improve adherence. - Evidence of economic and clinical outcomes were rare. |  |
| Rankin et al. (2018) [62] | - Weak evidence for improvement in appropriate polypharmacy. - Uncertain if improvement is clinically significant. - May be slightly beneficial in reducing potential prescribing omissions. | ***Favors interventions***  Outcome: Medication appropriateness   - Comparison of MAI scores from baseline to follow-up (n= 5), MD -4.76, (-9.20, -0.33)   Sensitivity analyses (removing studies with high risk of bias)   - Comparison of MAI scores from baseline to follow-up (n=4), MD -5.16, (-11.04, 0.72)] – **No effect** - Comparison of MAI scores from baseline to follow-up (n=3), MD -0.50 (-2.27, 1.28)]– **No effect**   Outcome: Potentially inappropriate medications   - Number of PIMs (n=7), SMD -0.22, (-0.38, -0.05)   ***No effect***  Outcome: Potentially inappropriate medications   - Proportion of patients with one or more PIM (n=11), RR 0.79, (0.61, 1.02)   Sensitivity analyses (removing studies with high risk of bias)   - Proportion of patients with one or more PIM (n= 10) – favors intervention [RR 0.79, (0.61, 1.02)] - Proportion of patients with one or more PIM (n= 9), RR 0.88, (0.72, 1.09)] |
| Thillainadesan et al. (2018) [67] | - No notable harm observed in intervention groups. - Can reduce potentially inappropriate medicines. - Uncertain impact on clinical outcome (functional status, falls, rehospitalisation, and mortality). |  |
| Thio et al. (2018) [68] | - Evidence of relapse of symptoms. - Evidence of success stopping medication. - Equivocal for clinical outcomes. |  |
| Monteiro et al. (2019) [61] | - Some significance of positive impact on potentially inappropriate medicines. - Evidence of adverse drug reactions, length of stay, mortality, and cost-effectiveness are lacking. |  |
| Almutairi et al. (2020) [43] | - Could improve medication appropriateness. - More evidence on clinical outcomes is needed. | ***Favors interventions***  Outcome: Medication appropriateness   - Medication appropriateness (n=10), RR 0.71 (0.60, 0.84)   Outcome: Medication appropriateness scales   - Mean difference in change of MAI score (n=2), MD -0.67 (-0.97, -0.37)   Subgroup analyses-Intervention   - Medication review (n=4), RR 0.62 (0.41, 0.93) - Multidisciplinary team meetings (n=1), RR 0.97 (0.92, 1.03) – **No effect** - Staff education (n=4), RR 0.66 (0.43, 1.01) – **No effect** - Computerised clinical decision, RR 0.78 (0.64, 0.95)   ***No effect***  Outcome: Hospital admissions   - Hospital admission (n=9), RR 1.00 (0.93, 1.06)   Outcome: Clinical and patient-centered outcomes   - Mortality (n=10), RR 0.98 (0.86, 1.11) - Falls (n=8), RR 1.06 (0.89, 1.26) - Quality of life (n=3), MD 0.16, (-0.13, 0.45) - BPSD (n=2), RR 0.68 (0.44, 1.06)] - Cognitive function (n=2), MD 0.69 (-1.25, 2.64)   Outcome: Adverse Drug Events   - ADEs (n=2), RR 1.04 (0.96, 1.13) |
| Bloomfield et al. (2020) [45] | - Some evidence for reduction in mortality and use of PIMs with comprehensive medication reviews. - Uncertain effects on quality of life and rates of hospitalizations and falls. - More evidence needed with regards to clinical outcomes. | ***Favors interventions***  Outcome: All-cause mortality   - Comprehensive medication review RCTs (n=12), OR 0.74 (0.58, 0.95)   ***No effect*** Outcome: Hospitalizations   - Comprehensive medication review RCTs (n=6), RR 1.07 (0.92, 1.26) - Meta-analyses not conducted for educational interventions and computerized decision support. |
| Earl et al. (2020) [52] | - Some statistically significant differences in polypharmacy or PIMs. |  |
| Shrestha et al. (2020) [13] | - Can improve medication appropriateness. - Limited evidence available but has potential for mortality reduction and cost savings. - Impact on quality of life and falls not clear. |  |
| Hart et al. (2020) [54] | - Limited evidence suggesting no reduction in overall fall risk increasing drug use following the fall‐related healthcare encounter. |  |
| Abu Fadaleh et al (2021) [42] | - Home Medication Review (HMR) provides a modest benefit in improving health care utilization and some medication measures. | ***No effect*** Outcome: Hospitalization   - Rate of hospitalizations and health service utilization (n=9), RR 0.91 (0.71, 1.15) |
| Alshammari et al (2021) [44] | - The reduction of PIMs varied in some studies, however some evidence showed improved patient outcomes. |  |
| Cardona et al (2021) [46] | - Limited evidence suggests hospital-initiated deprescribing interventions may reduce prescribing inappropriateness among older terminal patients in the short term, but evidence beyond 3 months is lacking for significant prevention of adverse events or health service utilization. |  |
| Christopher et al (2021) [48] | - There is evidence in the literature that community pharmacy-based interventions have a beneficial impact on clinical outcomes among older adults, including a reduction in inappropriate medicine use (e.g., sedative–hypnotic drugs), reduction in uncontrolled health problems, and reduction of adverse drug reactions. - There is limited or inconclusive evidence on the impact of community pharmacists’ interventions on hospitalization, quality of life, and other outcomes from randomized control trials. | ***Favors interventions***  Outcome: Medication discontinuation   - Benzodiazepine discontinuation after patient education intervention (n=2), RR 1.28 (1.20, 1.36)   ***No effect*** Outcome: Falls   - Rate of falls (n=2), RR 1.25 (0.78, 1.99)   Outcome: Hospitalizations   - Reducing hospitalizations (n=3), RR 0.72 (0.47, 1.12) |
| Ibrahim et al (2021) [55] | - Deprescribing could be safe, feasible, well tolerated and can lead to important benefits on geriatric conditions such as depression, function and frailty. |  |
| Laberge et al (2021) [59] | - Cannot draw a clear conclusion on the cost-effectiveness of interventions to optimize medication use. - Cost-effectiveness of an intervention may depend on contextual factors of the intervention and willingness-to-pay thresholds for a marginal improvement in medication use or a marginal reduction in adverse drug events. |  |
| Lee et al (2022) [60] | - Studies that instituted the STOPP/START criteria demonstrated statistically significant findings in medication changes. - A multifaceted deprescribing approach by an interdisciplinary team with a validated guideline for deprescribing is important for improving outcomes for high-risk older adults undergoing surgery during hospitalizations. |  |
| Saeed et al (2022) [64] | - Medicine optimization interventions may improve prescribing appropriateness in frail older inpatients. - Although their impact on frail patients’ clinical outcomes is unclear, these interventions seem to be safe and feasible for implementation in acute settings. |  |
| Shrestha et al (2021) [65] | - There is some evidence that deprescribing interventions involving dual purpose medications for older people with life-limiting illness and limited life expectancy can lower the risk of mortality and referral to acute care facilities. - There is insufficient evidence to establish its impact on other outcomes, such as health service utilization and adverse events. | ***Favors intervention***  Outcome: Mortality   - Reduction of mortality rate due to deprescription of dual-purpose medications (n=4), RR 0.59 (0.44, 0.79)   ***No Effect***  Outcome: Falls   - Reduction of fall rate due to description of dual-purpose medications (n=2) RR 0.87 (0.65, 1.17)   Outcome: Adverse events   - Reduction of non-vertebral fractures due to description of dual-purpose medications (n=2) RR 0.67 (0.10, 4.56)   Outcome: Hospitalizations   - Reduction of unplanned hospital admission due to description of dual-purpose medications (n=2) RR 1.12 (0.74, 1.69) |
| Rodrigues et al (2022) [63] | - Medication review is the most indicated intervention to promote the well-being of the hospitalized patients through the reduction of PIMs. |  |
| Stotzner et al (2022) [66] | - Improvement of drug-related outcomes can be achieved by interventions such as individualized medication review and educational approaches in psychiatric settings. - Changes in clinical outcomes, however, are often non substantial and generally underreported. |  |
| Clarkson et al (2023) [49] | - The evidence for deprescribing interventions in outpatient clinics is very limited. - The addition of a pharmacist in outpatient clinics and use of validated medication assessment tools appear to be enablers for implementing deprescribing interventions. |  |
| **Specific Medication Target and Optimizing Medication Use** | | |
| Page et al. (2016) [12] | - Some medications can be deprescribed without adverse changes in the specific health outcomes that the medications were intended to treat or in quality of life. - Feasible to reduce polypharmacy. - Patient specific descripting interventions reduced mortality. | ***Favors intervention***  Outcome: Mortality   - All pooled non-randomized studies (n=2), OR 0.32 (0.17, 0.60)   Outcome: Falls   - Number of falls per participant (n=3), MD -0.11 (-0.21, -0.02)   Outcome: Effect on medication regimen   - Reduction of total number of medications (n=2), MD -0.99 (-1.83, -0.14) - Reduction of PIMs (n=3), MD -0.49 (-0.70, -0.28)   ***No effect***  Outcome: Mortality   - All pooled randomized studies (n=10), OR 0.82 (0.61, 1.11)   Subgroup analysis- Intervention type   - Patient-specific interventions (n=8), OR 0.62 (0.43, 0.88) – **favors intervention** - Educational programs interventions (n=2), OR 1.21 (0.86, 1.69)   Subgroup analysis- Age   - Patients ≥ 80 years (n=7), OR 0.88 (0.58, 1.34) - Patients < 80 years (n=3), OR 0.64 (0.40, 1.04)   Subgroup analysis- Dementia status   - Participants living with dementia (n=5), OR 0.89 (0.63, 1.27) - Cognitively-intact people (n=5), OR 0.64 (0.36, 1.13)   Subgroup analysis- No. of medications   - Deprescribing of single medication class - antipsychotics (n=5), OR 0.59 (0.33, 1.07)   Outcome: Falls   - Risk of experiencing at least one fall (n=5), OR 0.65 (0.40, 1.05)   Outcome: Adverse drug withdrawal events   - Adverse drug withdrawal events associated with deprescribing of antipsychotics, randomized studies (n=3), OR 1.24 (0.51, 3.01)   Outcomes: Others   - Change in the neuropsychiatric index associated with deprescribing of antipsychotics, randomized studies (n=3), OR 2.65 (0.52, 4.77) - Successful withdrawal of deprescribing of benzodiazepines, randomized studies (n=3), OR 1.38 (0.21, 9.08) - Non-vertebral fractures of deprescribing bisphosphonates, non-randomized studies (n=3), OR 0.94 (0.50, 1.78)   ***Favors control*** Outcome: Surrogate biomarkers outcomes   - Systolic blood pressure: deprescribing diuretics (n=3), MD 9.73 (8.13, 11.33) - Diastolic blood pressure: deprescribing diuretics (n=3), MD 3.99 (3.04, 4.94) |

*Abbreviations:* BPSD= behavioral and psychological symptoms of dementia; BZD=benzodiazepines; CVD=cardiovascular disease; HbA1c=glycated hemoglobin; MD=mean difference; OR=odds ratio; PIMs=potentially inappropriate medications; RoB=risk of bias assessment; RR=risk ratio; SMD=standardized mean difference; TIA=transient ischemic attack.

^1^ Studies that focused on non-medication specific deprescribing, polypharmacy, PIM, medication appropriateness

**References**

(numbering matches the manuscript, only sources to S5 Table included)

￼

12. Page AT, Clifford RM, Potter K, Schwartz D, Etherton-Beer CD. The feasibility and effect of deprescribing in older adults on mortality and health: A systematic review and meta-analysis. Br J Clin Pharmacol. 2016;82(3):583-623.

13. Shrestha S, Poudel A, Steadman K, Nissen L. Outcomes of deprescribing interventions in older patients with life-limiting illness and limited life expectancy: A systematic review. Br J Clin Pharmacol. 2020;86(10):1931-45.

15. Iyer S, Naganathan V, McLachlan AJ, Le Couteur DG. Medication withdrawal trials in people aged 65 years and older: A systematic review. Drugs Aging. 2008;25(12):1021-31.

27. Black CD, Thompson W, Welch V, McCarthy L, Rojas-Fernandez C, Lochnan H, et al. Lack of evidence to guide deprescribing of antihyperglycemics: A systematic review. Diabetes Ther. 2017;8(1):23-31.

28. Seidu S, Kunutsor SK, Topsever P, Hambling CE, Cos FX, Khunti K. Deintensification in older patients with type 2 diabetes: A systematic review of approaches, rates and outcomes. Diabetes Obes Metab. 2019;21(7):1668-79.

29. Nakham A, Myint PK, Bond CM, Newlands R, Loke YK, Cruickshank M. Interventions to reduce anticholinergic burden in adults aged 65 and older: A systematic review. J Am Med Dir Assoc. 2020;21(2):172-80.e5.

30. Salahudeen MS, Alfahmi A, Farooq A, Akhtar M, Ajaz S, Alotaibi S, et al. Effectiveness of interventions to improve the anticholinergic prescribing practice in older adults: A systematic review. J Clin Med. 2022;11(3).

31. Reeve E, Jordan V, Thompson W, Sawan M, Todd A, Gammie TM, et al. Withdrawal of antihypertensive drugs in older people. Cochrane Database Syst Rev. 2020;6(6):CD012572.

32. Dou C, Rebane J, Bardal S. Interventions to improve benzodiazepine tapering success in the elderly: A systematic review. Aging Ment Health. 2019;23(4):411-6.

33. Reeve E, Ong M, Wu A, Jansen J, Petrovic M, Gnjidic D. A systematic review of interventions to deprescribe benzodiazepines and other hypnotics among older people. Eur J Clin Pharmacol. 2017;73(8):927-35.

34. Hoyle DJ, Bindoff IK, Clinnick LM, Peterson GM, Westbury JL. Clinical and economic outcomes of interventions to reduce antipsychotic and benzodiazepine use within nursing homes: A systematic review. Drugs Aging. 2018;35(2):123-34.

35. Parr JM, Kavanagh DJ, Cahill L, Mitchell G, McD Young R. Effectiveness of current treatment approaches for benzodiazepine discontinuation: A meta-analysis. Addiction. 2009;104(1):13-24.

36. Thompson-Coon J, Abbott R, Rogers M, Whear R, Pearson S, Lang I, et al. Interventions to reduce inappropriate prescribing of antipsychotic medications in people with dementia resident in care homes: A systematic review. J Am Med Dir Assoc. 2014;15(10):706-18.

37. Sheehan R, Strydom A, Brown E, Marston L, Hassiotis A. Association of focused medication review with optimization of psychotropic drug prescribing: A systematic review and meta-analysis. JAMA Netw Open. 2018;1(6):[e183750.

38. Nishtala PS, McLachlan AJ, Bell JS, Chen TF. Psychotropic prescribing in long-term care facilities: Impact of medication reviews and educational interventions. Am J Geriatr Psychiatry. 2008;16(8):621-32.

39. Lynch T, Ryan C, Hughes CM, Presseau J, van Allen ZM, Bradley CP, Cadogan CA. Brief interventions targeting long-term benzodiazepine and z-drug use in primary care: A systematic review and meta-analysis. Addiction. 2020;115(9):1618-39.

40. Ribeiro PRS, Schlindwein AD. Benzodiazepine deprescription strategies in chronic users: A systematic review. Fam Pract. 2021;38(5):684-93.

41. Wilsdon TD, Hendrix I, Thynne TR, Mangoni AA. Effectiveness of interventions to deprescribe inappropriate proton pump inhibitors in older adults. Drugs Aging. 2017;34(4):265-87.

42. Abu Fadaleh SM, Charrois TL, Makhinova T, Eurich DT, Sholeh R, Sadowski CA. The effect of home medication review in community-dwelling older adults: A systematic review. J Public Health (Berl). 2022;30:1857–72.

43. Almutairi H, Stafford A, Etherton-Beer C, Flicker L. Optimisation of medications used in residential aged care facilities: A systematic review and meta-analysis of randomised controlled trials. BMC Geriatr. 2020;20(1):236.

44. Alshammari H, Al-Saeed E, Ahmed Z, Aslanpour Z. Reviewing potentially inappropriate medication in hospitalized patients over 65 using explicit criteria: A systematic literature review. Drug Healthc Patient Saf. 2021;13:183-210.

45. Bloomfield HE, Greer N, Linsky AM, Bolduc J, Naidl T, Vardeny O, et al. Deprescribing for community-dwelling older adults: A systematic review and meta-analysis. J Gen Intern Med. 2020;35(11):3323-32.

46. Cardona M, Stehlik P, Fawzy P, Byambasuren O, Anderson J, Clark J, et al. Effectiveness and sustainability of deprescribing for hospitalized older patients near end of life: A systematic review. Expert Opin Drug Saf. 2021;20(1):81-91.

47. Castelino RL, Bajorek BV, Chen TF. Targeting suboptimal prescribing in the elderly: A review of the impact of pharmacy services. Ann Pharmacother. 2009;43(6):1096-106.

48. Christopher CM, Kc B, Blebil A, Alex D, Ibrahim MIM, Ismail N, Alrasheedy AA. Clinical and humanistic outcomes of community pharmacy-based healthcare interventions regarding medication use in older adults: A systematic review and meta-analysis. Healthcare (Basel). 2021;9(11):1577.

49. Clarkson L, Hart L, Lam AK, Khoo TK. Reducing inappropriate polypharmacy for older patients at specialist outpatient clinics: A systematic review. Curr Med Res Opin. 2023;39(4):545-54.

50. Clyne B, Fitzgerald C, Quinlan A, Hardy C, Galvin R, Fahey T, Smith SM. Interventions to address potentially inappropriate prescribing in community-dwelling older adults: A systematic review of randomized controlled trials. J Am Geriatr Soc. 2016;64(6):1210-22.

51. Dalton K, O'Brien G, O'Mahony D, Byrne S. Computerised interventions designed to reduce potentially inappropriate prescribing in hospitalised older adults: A systematic review and meta-analysis. Age Ageing. 2018;47(5):670-8.

52. Earl TR, Katapodis ND, Schneiderman SR, Shoemaker-Hunt SJ. Using deprescribing practices and the screening tool of older persons' potentially inappropriate prescriptions criteria to reduce harm and preventable adverse drug events in older adults. J Patient Saf. 2020;16(3S Suppl 1):S23-S35.

53. Hansen CR, O'Mahony D, Kearney PM, Sahm LJ, Cullinan S, Huibers CJA, et al. Identification of behaviour change techniques in deprescribing interventions: A systematic review and meta-analysis. Br J Clin Pharmacol. 2018;84(12):2716-28.

54. Hart LA, Phelan EA, Yi JY, Marcum ZA, Gray SL. Use of fall risk-increasing drugs around a fall-related injury in older adults: A systematic review. J Am Geriatr Soc. 2020;68(6):1334-43.

55. Ibrahim K, Cox NJ, Stevenson JM, Lim S, Fraser SDS, Roberts HC. A systematic review of the evidence for deprescribing interventions among older people living with frailty. BMC Geriatr. 2021;21(1):258.

56. Johansson T, Abuzahra ME, Keller S, Mann E, Faller B, Sommerauer C, et al. Impact of strategies to reduce polypharmacy on clinically relevant endpoints: A systematic review and meta-analysis. Br J Clin Pharmacol. 2016;82(2):532-48.

57. Kallio SE, Kiiski A, Airaksinen MSA, Mantyla AT, Kumpusalo-Vauhkonen AEJ, Jarvensivu TP, Pohjanoksa-Mantyla MK. Community pharmacists' contribution to medication reviews for older adults: A systematic review. J Am Geriatr Soc. 2018;66(8):1613-20.

58. Kaur S, Mitchell G, Vitetta L, Roberts MS. Interventions that can reduce inappropriate prescribing in the elderly: A systematic review. Drugs Aging. 2009;26(12):1013-28.

59. Laberge M, Sirois C, Lunghi C, Gaudreault M, Nakamura Y, Bolduc C, Laroche ML. Economic evaluations of interventions to optimize medication use in older adults with polypharmacy and multimorbidity: A systematic review. Clin Interv Aging. 2021;16:767-79.

60. Lee JW, Li M, Boyd CM, Green AR, Szanton SL. Preoperative deprescribing for medical optimization of older adults undergoing surgery: A systematic review. J Am Med Dir Assoc. 2022;23(4):528-36 e2.

61. Monteiro L, Maricoto T, Solha I, Ribeiro-Vaz I, Martins C, Monteiro-Soares M. Reducing potentially inappropriate prescriptions for older patients using computerized decision support tools: Systematic review. J Med Internet Res. 2019;21(11):e15385.

62. Rankin A, Cadogan CA, Patterson SM, Kerse N, Cardwell CR, Bradley MC, et al. Interventions to improve the appropriate use of polypharmacy for older people. Cochrane Database Syst Rev. 2018;9:CD008165.

63. Rodrigues DA, Placido AI, Mateos-Campos R, Figueiras A, Herdeiro MT, Roque F. Effectiveness of interventions to reduce potentially inappropriate medication in older patients: A systematic review. Front Pharmacol. 2022;12:777655.

64. Saeed D, Carter G, Parsons C. Interventions to improve medicines optimisation in frail older patients in secondary and acute care settings: A systematic review of randomised controlled trials and non-randomised studies. Int J Clin Pharm. 2022;44(1):15-26.

65. Shrestha S, Poudel A, Cardona M, Steadman KJ, Nissen LM. Impact of deprescribing dual-purpose medications on patient-related outcomes for older adults near end-of-life: A systematic review and meta-analysis. Ther Adv Drug Saf. 2021;12:20420986211052343.

66. Stotzner P, Ferrebus Abate RE, Henssler J, Seethaler M, Just SA, Brandl EJ. Structured interventions to optimize polypharmacy in psychiatric treatment and nursing homes: A systematic review. J Clin Psychopharmacol. 2022;42(2):169-87.

67. Thillainadesan J, Gnjidic D, Green S, Hilmer SN. Impact of deprescribing interventions in older hospitalised patients on prescribing and clinical outcomes: A systematic review of randomised trials. Drugs Aging. 2018;35(4):303-19.

68. Thio SL, Nam J, van Driel ML, Dirven T, Blom JW. Effects of discontinuation of chronic medication in primary care: A systematic review of deprescribing trials. Br J Gen Pract. 2018;68(675):e663-e72.

69. Tjia J, Velten SJ, Parsons C, Valluri S, Briesacher BA. Studies to reduce unnecessary medication use in frail older adults: A systematic review. Drugs Aging. 2013;30(5):285-307.

70. Verrue CL, Petrovic M, Mehuys E, Remon JP, Vander Stichele R. Pharmacists' interventions for optimization of medication use in nursing homes : A systematic review. Drugs Aging. 2009;26(1):37-49.

71. Walsh KA, O'Riordan D, Kearney PM, Timmons S, Byrne S. Improving the appropriateness of prescribing in older patients: A systematic review and meta-analysis of pharmacists' interventions in secondary care. Age Ageing. 2016;45(2):201-9.
