# Supplementary material for: Deprescribing interventions in older adults: An overview of systematic reviews
Source: PLoS One. 2024 Jun 17;19(6):e0305215. doi: 10.1371/journal.pone.0305215 (PMC11182547; doi:10.1371/journal.pone.0305215)
Supplement: S6 Table — (DOCX) [file pone.0305215.s010.docx]

S6 Table. AMSTAR 2 quality assessment of included systematic reviews.

|  | **AMSTAR 2 Domains [25]** | | | | | | | | | | | | | | | | **Confidence Rating** |
| --- | --- | --- | --- | --- | --- | --- | --- | --- | --- | --- | --- | --- | --- | --- | --- | --- | --- |
|  | **1** | **2** | **3** | **4** | **5** | **6** | **7** | **8** | **9** | **10** | **11** | **12** | **13** | **14** | **15** | **16** |  |
| Abu Fadaleh 2022 [42] |  | - |  | - |  |  | x |  |  | x |  |  |  |  | x |  | Critically low |
| Almutairi 2020 [43] |  |  | x | x | x |  | x | x |  | x |  | x | x |  |  |  | Critically low |
| Alshammari 2021 [44] | x | - |  | x |  | x | x | - | x | x | NA | NA | x | x | NA |  | Critically low |
| Black 2017 [27] |  |  | x | - |  |  | x |  |  | x | NA | NA | x |  | NA |  | Critically low |
| Bloomfield 2020 [45] |  |  |  | x |  |  | x |  |  |  | x |  |  | x | x |  | Critically low |
| Cardona 2021 [46] |  |  |  |  |  |  |  |  |  |  | NA | NA |  |  | NA |  | High |
| Castelino 2009 [47] | x | x | x | x | x | x |  | - | x | x | NA | NA | x |  | NA | x | Critically low |
| Christopher 2021 [48] |  |  | x | x |  | x | x | x |  | x |  |  | x |  | x |  | Critically low |
| Clarkson 2023 [49] | x | x | x | x | x | x | x | - |  | x | NA | NA |  |  | NA |  | Critically low |
| Clyne 2016 [50] |  | x | x | - |  | x | x |  |  | x | NA | NA |  |  | NA |  | Critically low |
| Thompson-Coon 2014 [36] |  | - | x | - |  |  | x | - |  | x | NA | NA |  |  | NA |  | Low |
| Dalton 2018 [51] |  |  |  | - |  |  | x | - |  | x |  |  |  |  | x |  | Critically low |
| Dou 2019 [32] |  | x | x | x | x | x | x |  |  | x | x | x |  |  | x |  | Critically low |
| Earl 2020 [52] | x | x | x | x | x | x | x | - | x | x | NA | NA | x | x | NA |  | Critically low |
| Hansen 2018 [53] |  |  |  | - |  |  | x | x |  | x |  |  |  |  | x |  | Critically low |
| Hart 2020 [54] |  | x | x | x |  |  | x | x |  | x | NA | NA | x | x | NA |  | Critically low |
| Hoyle 2018 [34] |  | - | x | x |  | x | x | x |  | x | NA | NA |  |  | NA |  | Critically low |
| Ibrahim 2021 [55] |  |  |  | - |  |  | x |  |  | x | NA | NA |  |  | NA |  | Low |
| Iyer 2008 [15] |  | x | x | x | x |  | x | - | x | x | NA | NA | x | x | NA |  | Critically low |
| Johansson 2016 [56] |  | x |  | - |  |  |  |  | x | x | x | x | x | x | x |  | Critically low |
| Kallio 2018 [57] |  | x | x | x |  | x | x |  | x | x | NA | NA | x |  | NA |  | Critically low |
| Kaur 2009 [58] | x | x | x | x | x | x | x | x | x | x | NA | NA | x |  | NA |  | Critically low |
| Laberge 2021[59] |  | x |  | - |  |  | x | x | x | x | NA | NA |  |  | NA |  | Critically low |
| Lee 2022 [60] | x | x | x | x |  | x | x | x |  | x | NA | NA |  |  | NA |  | Critically low |
| Lynch 2020 [39] |  | x | x | x |  |  | x |  |  | x |  |  |  |  |  |  | Critically low |
| Monteiro 2019 [61] | x | - | x |  |  |  | x | - | x | x | NA | NA |  |  | NA |  | Critically low |
| Nakham 2020 [29] |  |  | x | - |  |  | x | - | - | x | NA | NA | x |  | NA |  | Critically low |
| Nishtala 2008 [38] |  | x | x | x |  | x |  | x | x | x |  | x | x | x | x | x | Critically low |
| Page 2016 [12] |  |  | x | x |  |  |  |  |  |  |  | x |  |  | x |  | Critically low |
| Parr 2009 [35] |  | x | x | x |  | x | x | x | x | x | x | x | x | x | x |  | Critically low |
| Rankin 2018 [62] |  |  |  | - |  |  |  |  |  | x | x |  |  |  |  |  | Low |
| Reeve 2017 [33] | x | x | x | x |  |  | x | x |  | x | NA | NA |  | x | NA |  | Critically low |
| Reeve 2020 [31] |  |  | x |  |  |  |  |  |  |  |  |  |  |  | x |  | Low |
| Ribeiro 2021 [40] |  | - | x | - |  |  | x |  |  | x | NA | NA |  |  | NA |  | Low |
| Rodrigues 2022[63] |  |  |  | x |  |  | x | - |  | x | NA | NA |  |  | NA |  | Critically low |
| Saeed 2022 [64] |  |  |  | x |  |  | x |  |  | x | NA | NA |  |  | NA |  | Critically low |
| Salahudeen 2022 [30] |  | x | x | x |  |  | x |  |  | x | NA | NA |  |  | NA |  | Critically low |
| Seidu 2019 [28] |  |  |  | - |  |  | x |  |  | x | NA | NA |  | x | NA |  | Low |
| Sheehan 2018 [37] |  | - | x | - |  | x | x |  |  |  | x | x |  | x | x |  | Critically low |
| Shrestha 2020 [13] |  |  | x | x |  | x | x |  |  | x | NA | NA | x | x | NA |  | Critically low |
| Shrestha 2021 [65] |  |  |  | x |  |  | x |  |  | x |  |  |  |  | x |  | Critically low |
| Stotzner 2022 [66] | x |  | x | x |  | x | x | - |  | x | NA | NA | x |  | NA |  | Critically low |
| Thillainadesan 2018 [67] |  | x | x | x |  | x | x |  |  | x | NA | NA |  |  | NA |  | Critically low |
| Thio 2018 [68] |  | x | x | - |  |  | x | - |  | x | NA | NA |  |  | NA |  | Critically low |
| Tjia 2013 [69] |  | x | x | x |  |  | x | x | x | x | NA | NA | x |  | NA |  | Critically low |
| Verrue 2009 [70] | x | x | x | x | x | x | x | - | x | x | NA | NA | x | x | NA |  | Critically low |
| Walsh 2016 [71] |  | - | x | - |  |  | x | x |  | x |  |  |  |  | x |  | Critically low |
| Wilsdon 2017 [41] |  |  |  | - |  |  | x |  | - | x | NA | NA | x |  | NA |  | Critically low |

“x” denotes a weakness in that domain, while “–“ denotes a partial weakness in that domain. Shaded columns represent domains considered critical by Shea et al. One critical flaw results in an overall confidence rating of low, while more than one critical flaw results in an overall confidence rating of critically low.

Domain description: 1 Use of PICO (Population, Intervention, Comparison, Outcome) in research question, 2 Prespecified protocol, 3 Explanation of study designs included, 4 Search strategy, 5 Study selection in duplicate, 6 Data extraction in duplicate, 7 Justified exclusion of studies, 8 Description of included studies, 9 Risk of bias (RoB) assessment, 10 Report of source of funding of primary studies, 11 (Meta-analysis) Appropriate statistical combination of results, 12 (Meta-analysis) RoB assessment on results of meta-analysis, 13 Accounting for RoB in discussion of results, 14 Explanation of observed heterogeneity, 15 (Meta-analysis) Publication bias, 16 Report on potential conflict of interest

**References**

(numbering matches the manuscript, only sources to S6 Table included)

￼

12. Page AT, Clifford RM, Potter K, Schwartz D, Etherton-Beer CD. The feasibility and effect of deprescribing in older adults on mortality and health: A systematic review and meta-analysis. Br J Clin Pharmacol. 2016;82(3):583-623.

13. Shrestha S, Poudel A, Steadman K, Nissen L. Outcomes of deprescribing interventions in older patients with life-limiting illness and limited life expectancy: A systematic review. Br J Clin Pharmacol. 2020;86(10):1931-45.

15. Iyer S, Naganathan V, McLachlan AJ, Le Couteur DG. Medication withdrawal trials in people aged 65 years and older: A systematic review. Drugs Aging. 2008;25(12):1021-31.

27. Black CD, Thompson W, Welch V, McCarthy L, Rojas-Fernandez C, Lochnan H, et al. Lack of evidence to guide deprescribing of antihyperglycemics: A systematic review. Diabetes Ther. 2017;8(1):23-31.

28. Seidu S, Kunutsor SK, Topsever P, Hambling CE, Cos FX, Khunti K. Deintensification in older patients with type 2 diabetes: A systematic review of approaches, rates and outcomes. Diabetes Obes Metab. 2019;21(7):1668-79.

29. Nakham A, Myint PK, Bond CM, Newlands R, Loke YK, Cruickshank M. Interventions to reduce anticholinergic burden in adults aged 65 and older: A systematic review. J Am Med Dir Assoc. 2020;21(2):172-80.e5.

30. Salahudeen MS, Alfahmi A, Farooq A, Akhtar M, Ajaz S, Alotaibi S, et al. Effectiveness of interventions to improve the anticholinergic prescribing practice in older adults: A systematic review. J Clin Med. 2022;11(3).

31. Reeve E, Jordan V, Thompson W, Sawan M, Todd A, Gammie TM, et al. Withdrawal of antihypertensive drugs in older people. Cochrane Database Syst Rev. 2020;6(6):CD012572.

32. Dou C, Rebane J, Bardal S. Interventions to improve benzodiazepine tapering success in the elderly: A systematic review. Aging Ment Health. 2019;23(4):411-6.

33. Reeve E, Ong M, Wu A, Jansen J, Petrovic M, Gnjidic D. A systematic review of interventions to deprescribe benzodiazepines and other hypnotics among older people. Eur J Clin Pharmacol. 2017;73(8):927-35.

34. Hoyle DJ, Bindoff IK, Clinnick LM, Peterson GM, Westbury JL. Clinical and economic outcomes of interventions to reduce antipsychotic and benzodiazepine use within nursing homes: A systematic review. Drugs Aging. 2018;35(2):123-34.

35. Parr JM, Kavanagh DJ, Cahill L, Mitchell G, McD Young R. Effectiveness of current treatment approaches for benzodiazepine discontinuation: A meta-analysis. Addiction. 2009;104(1):13-24.

36. Thompson-Coon J, Abbott R, Rogers M, Whear R, Pearson S, Lang I, et al. Interventions to reduce inappropriate prescribing of antipsychotic medications in people with dementia resident in care homes: A systematic review. J Am Med Dir Assoc. 2014;15(10):706-18.

37. Sheehan R, Strydom A, Brown E, Marston L, Hassiotis A. Association of focused medication review with optimization of psychotropic drug prescribing: A systematic review and meta-analysis. JAMA Netw Open. 2018;1(6):[e183750.

38. Nishtala PS, McLachlan AJ, Bell JS, Chen TF. Psychotropic prescribing in long-term care facilities: Impact of medication reviews and educational interventions. Am J Geriatr Psychiatry. 2008;16(8):621-32.

39. Lynch T, Ryan C, Hughes CM, Presseau J, van Allen ZM, Bradley CP, Cadogan CA. Brief interventions targeting long-term benzodiazepine and z-drug use in primary care: A systematic review and meta-analysis. Addiction. 2020;115(9):1618-39.

40. Ribeiro PRS, Schlindwein AD. Benzodiazepine deprescription strategies in chronic users: A systematic review. Fam Pract. 2021;38(5):684-93.

41. Wilsdon TD, Hendrix I, Thynne TR, Mangoni AA. Effectiveness of interventions to deprescribe inappropriate proton pump inhibitors in older adults. Drugs Aging. 2017;34(4):265-87.

42. Abu Fadaleh SM, Charrois TL, Makhinova T, Eurich DT, Sholeh R, Sadowski CA. The effect of home medication review in community-dwelling older adults: A systematic review. J Public Health (Berl). 2022;30:1857–72.

43. Almutairi H, Stafford A, Etherton-Beer C, Flicker L. Optimisation of medications used in residential aged care facilities: A systematic review and meta-analysis of randomised controlled trials. BMC Geriatr. 2020;20(1):236.

44. Alshammari H, Al-Saeed E, Ahmed Z, Aslanpour Z. Reviewing potentially inappropriate medication in hospitalized patients over 65 using explicit criteria: A systematic literature review. Drug Healthc Patient Saf. 2021;13:183-210.

45. Bloomfield HE, Greer N, Linsky AM, Bolduc J, Naidl T, Vardeny O, et al. Deprescribing for community-dwelling older adults: A systematic review and meta-analysis. J Gen Intern Med. 2020;35(11):3323-32.

46. Cardona M, Stehlik P, Fawzy P, Byambasuren O, Anderson J, Clark J, et al. Effectiveness and sustainability of deprescribing for hospitalized older patients near end of life: A systematic review. Expert Opin Drug Saf. 2021;20(1):81-91.

47. Castelino RL, Bajorek BV, Chen TF. Targeting suboptimal prescribing in the elderly: A review of the impact of pharmacy services. Ann Pharmacother. 2009;43(6):1096-106.

48. Christopher CM, Kc B, Blebil A, Alex D, Ibrahim MIM, Ismail N, Alrasheedy AA. Clinical and humanistic outcomes of community pharmacy-based healthcare interventions regarding medication use in older adults: A systematic review and meta-analysis. Healthcare (Basel). 2021;9(11):1577.

49. Clarkson L, Hart L, Lam AK, Khoo TK. Reducing inappropriate polypharmacy for older patients at specialist outpatient clinics: A systematic review. Curr Med Res Opin. 2023;39(4):545-54.

50. Clyne B, Fitzgerald C, Quinlan A, Hardy C, Galvin R, Fahey T, Smith SM. Interventions to address potentially inappropriate prescribing in community-dwelling older adults: A systematic review of randomized controlled trials. J Am Geriatr Soc. 2016;64(6):1210-22.

51. Dalton K, O'Brien G, O'Mahony D, Byrne S. Computerised interventions designed to reduce potentially inappropriate prescribing in hospitalised older adults: A systematic review and meta-analysis. Age Ageing. 2018;47(5):670-8.

52. Earl TR, Katapodis ND, Schneiderman SR, Shoemaker-Hunt SJ. Using deprescribing practices and the screening tool of older persons' potentially inappropriate prescriptions criteria to reduce harm and preventable adverse drug events in older adults. J Patient Saf. 2020;16(3S Suppl 1):S23-S35.

53. Hansen CR, O'Mahony D, Kearney PM, Sahm LJ, Cullinan S, Huibers CJA, et al. Identification of behaviour change techniques in deprescribing interventions: A systematic review and meta-analysis. Br J Clin Pharmacol. 2018;84(12):2716-28.

54. Hart LA, Phelan EA, Yi JY, Marcum ZA, Gray SL. Use of fall risk-increasing drugs around a fall-related injury in older adults: A systematic review. J Am Geriatr Soc. 2020;68(6):1334-43.

55. Ibrahim K, Cox NJ, Stevenson JM, Lim S, Fraser SDS, Roberts HC. A systematic review of the evidence for deprescribing interventions among older people living with frailty. BMC Geriatr. 2021;21(1):258.

56. Johansson T, Abuzahra ME, Keller S, Mann E, Faller B, Sommerauer C, et al. Impact of strategies to reduce polypharmacy on clinically relevant endpoints: A systematic review and meta-analysis. Br J Clin Pharmacol. 2016;82(2):532-48.

57. Kallio SE, Kiiski A, Airaksinen MSA, Mantyla AT, Kumpusalo-Vauhkonen AEJ, Jarvensivu TP, Pohjanoksa-Mantyla MK. Community pharmacists' contribution to medication reviews for older adults: A systematic review. J Am Geriatr Soc. 2018;66(8):1613-20.

58. Kaur S, Mitchell G, Vitetta L, Roberts MS. Interventions that can reduce inappropriate prescribing in the elderly: A systematic review. Drugs Aging. 2009;26(12):1013-28.

59. Laberge M, Sirois C, Lunghi C, Gaudreault M, Nakamura Y, Bolduc C, Laroche ML. Economic evaluations of interventions to optimize medication use in older adults with polypharmacy and multimorbidity: A systematic review. Clin Interv Aging. 2021;16:767-79.

60. Lee JW, Li M, Boyd CM, Green AR, Szanton SL. Preoperative deprescribing for medical optimization of older adults undergoing surgery: A systematic review. J Am Med Dir Assoc. 2022;23(4):528-36 e2.

61. Monteiro L, Maricoto T, Solha I, Ribeiro-Vaz I, Martins C, Monteiro-Soares M. Reducing potentially inappropriate prescriptions for older patients using computerized decision support tools: Systematic review. J Med Internet Res. 2019;21(11):e15385.

62. Rankin A, Cadogan CA, Patterson SM, Kerse N, Cardwell CR, Bradley MC, et al. Interventions to improve the appropriate use of polypharmacy for older people. Cochrane Database Syst Rev. 2018;9:CD008165.

63. Rodrigues DA, Placido AI, Mateos-Campos R, Figueiras A, Herdeiro MT, Roque F. Effectiveness of interventions to reduce potentially inappropriate medication in older patients: A systematic review. Front Pharmacol. 2022;12:777655.

64. Saeed D, Carter G, Parsons C. Interventions to improve medicines optimisation in frail older patients in secondary and acute care settings: A systematic review of randomised controlled trials and non-randomised studies. Int J Clin Pharm. 2022;44(1):15-26.

65. Shrestha S, Poudel A, Cardona M, Steadman KJ, Nissen LM. Impact of deprescribing dual-purpose medications on patient-related outcomes for older adults near end-of-life: A systematic review and meta-analysis. Ther Adv Drug Saf. 2021;12:20420986211052343.

66. Stotzner P, Ferrebus Abate RE, Henssler J, Seethaler M, Just SA, Brandl EJ. Structured interventions to optimize polypharmacy in psychiatric treatment and nursing homes: A systematic review. J Clin Psychopharmacol. 2022;42(2):169-87.

67. Thillainadesan J, Gnjidic D, Green S, Hilmer SN. Impact of deprescribing interventions in older hospitalised patients on prescribing and clinical outcomes: A systematic review of randomised trials. Drugs Aging. 2018;35(4):303-19.

68. Thio SL, Nam J, van Driel ML, Dirven T, Blom JW. Effects of discontinuation of chronic medication in primary care: A systematic review of deprescribing trials. Br J Gen Pract. 2018;68(675):e663-e72.

69. Tjia J, Velten SJ, Parsons C, Valluri S, Briesacher BA. Studies to reduce unnecessary medication use in frail older adults: A systematic review. Drugs Aging. 2013;30(5):285-307.

70. Verrue CL, Petrovic M, Mehuys E, Remon JP, Vander Stichele R. Pharmacists' interventions for optimization of medication use in nursing homes : A systematic review. Drugs Aging. 2009;26(1):37-49.

71. Walsh KA, O'Riordan D, Kearney PM, Timmons S, Byrne S. Improving the appropriateness of prescribing in older patients: A systematic review and meta-analysis of pharmacists' interventions in secondary care. Age Ageing. 2016;45(2):201-9.
