# Supplementary material for: Deprescribing interventions in older adults: An overview of systematic reviews
Source: PLoS One. 2024 Jun 17;19(6):e0305215. doi: 10.1371/journal.pone.0305215 (PMC11182547; doi:10.1371/journal.pone.0305215)
Supplement: S7 Table — (DOCX) [file pone.0305215.s011.docx]

S7 Table. Summary of the effect of deprescribing interventions on outcome themes, grouped by specific medication classes.

|  | **A** | **B** | **C** | **D** | **E** | **F** |
| --- | --- | --- | --- | --- | --- | --- |
| **Each review for each outcome showed evidence of:** | **Beneficial effect only** | **Beneficial and no effect** | **No effect** | **Beneficial, no effect and negative effect** | **No effect and negative effect** | **Negative effect only** |
| **Anticholinergics** | - Medication appropriateness [29] - Adverse effects [29] | - Medication reduction[29] - Medication appropriateness [30] | - Cognition [29] |  |  |  |
| **Antihyperglycemics** | - Medication reduction [27] |  | - Surrogate biomarker outcomes [27,28] - Mortality [27,28] - Other patient-reported outcome measures [27,28] |  |  |  |
| **Antihypertensives** | - Adverse effects [31] |  | - Quality of life [31] |  |  |  |
| **Proton Pump inhibitors** | - Medication reduction [41] |  |  |  |  |  |
| **Psychotropics** | - Medication reduction [33, 34, 38] - Adverse effects [37] | - Medication reduction [32,36] - Quality of life [33] - Cognition [34] | - Mortality [37] - Quality of life [34,37] - Cognition [33] - Falls [34,37] - Hospitalizations [34,38] - Adverse effects [32,38] - Other patient-reported outcome measures [34,37,38] | - Other patient-reported outcome measures [33, 37] | - Other patient-reported outcome measures [34] | - Adverse effects (ADWE) [33] - Other patient-reported outcome measures [34,38] |

Other patient-reported outcome measures**: Col C**: Activities of Daily Living [34], hypoglycemia-but was not classified as an adverse effect so reported here [27,28], behavior/agitation [34,38], depressive symptoms [37], function [37], sleep [34]. **Col D** sleep quality [33], behavioral and psychological symptoms of dementia [37]; **Col E**: depression [34]; **Col F**: depression [38], apathy and psychiatric symptoms [34].

**References**

(numbering matches the manuscript, only sources to S7 Table included)

￼

27. Black CD, Thompson W, Welch V, McCarthy L, Rojas-Fernandez C, Lochnan H, et al. Lack of evidence to guide deprescribing of antihyperglycemics: A systematic review. Diabetes Ther. 2017;8(1):23-31.

28. Seidu S, Kunutsor SK, Topsever P, Hambling CE, Cos FX, Khunti K. Deintensification in older patients with type 2 diabetes: A systematic review of approaches, rates and outcomes. Diabetes Obes Metab. 2019;21(7):1668-79.

29. Nakham A, Myint PK, Bond CM, Newlands R, Loke YK, Cruickshank M. Interventions to reduce anticholinergic burden in adults aged 65 and older: A systematic review. J Am Med Dir Assoc. 2020;21(2):172-80.e5.

30. Salahudeen MS, Alfahmi A, Farooq A, Akhtar M, Ajaz S, Alotaibi S, et al. Effectiveness of interventions to improve the anticholinergic prescribing practice in older adults: A systematic review. J Clin Med. 2022;11(3).

31. Reeve E, Jordan V, Thompson W, Sawan M, Todd A, Gammie TM, et al. Withdrawal of antihypertensive drugs in older people. Cochrane Database Syst Rev. 2020;6(6):CD012572.

32. Dou C, Rebane J, Bardal S. Interventions to improve benzodiazepine tapering success in the elderly: A systematic review. Aging Ment Health. 2019;23(4):411-6.

33. Reeve E, Ong M, Wu A, Jansen J, Petrovic M, Gnjidic D. A systematic review of interventions to deprescribe benzodiazepines and other hypnotics among older people. Eur J Clin Pharmacol. 2017;73(8):927-35.

34. Hoyle DJ, Bindoff IK, Clinnick LM, Peterson GM, Westbury JL. Clinical and economic outcomes of interventions to reduce antipsychotic and benzodiazepine use within nursing homes: A systematic review. Drugs Aging. 2018;35(2):123-34.

36. Thompson-Coon J, Abbott R, Rogers M, Whear R, Pearson S, Lang I, et al. Interventions to reduce inappropriate prescribing of antipsychotic medications in people with dementia resident in care homes: A systematic review. J Am Med Dir Assoc. 2014;15(10):706-18.

37. Sheehan R, Strydom A, Brown E, Marston L, Hassiotis A. Association of focused medication review with optimization of psychotropic drug prescribing: A systematic review and meta-analysis. JAMA Netw Open. 2018;1(6):[e183750.

38. Nishtala PS, McLachlan AJ, Bell JS, Chen TF. Psychotropic prescribing in long-term care facilities: Impact of medication reviews and educational interventions. Am J Geriatr Psychiatry. 2008;16(8):621-32.

41. Wilsdon TD, Hendrix I, Thynne TR, Mangoni AA. Effectiveness of interventions to deprescribe inappropriate proton pump inhibitors in older adults. Drugs Aging. 2017;34(4):265-87.
