# Supplementary material for: Deprescribing interventions in older adults: An overview of systematic reviews
Source: PLoS One. 2024 Jun 17;19(6):e0305215. doi: 10.1371/journal.pone.0305215 (PMC11182547; doi:10.1371/journal.pone.0305215)
Supplement: Attachment — Submitted filename: Comments to authors.docx [file pone.0305215.s012.docx]

Manuscript ID: PONE-D-23-42998

**Effectiveness of Deprescribing Interventions in Older Adults: An Overview of Systematic Reviews**

The authors have synthesized evidence from reviews to provide an overarching overview of effectiveness of Deprescribing interventions in older adults. This is a valuable piece of work and appreciate the authors’ effort. Some of my comments are as follows:

1. **Introduction – L83:** The authors state that earlier work focused on reducing polypharmacy by addressing the appropriateness of medications and discontinuing those where harms outweigh benefits. As a reader, I would want to look into some of these studies. The authors can highlight these studies providing reference(s).
2. **Method – Search strategy - L120:** The term information specialist might not be a common word in some parts of the world. It might be worth defining it or providing alternative word in a bracket such as ‘librarian’ for wider audience.
3. **Method – Table 1 – Population:** I appreciate that the authors have considered studies conducted in long-term care even if age was not provided. My comment is related to the definition of population. The authors have considered aged 60 years and above as older adults. However, the authors have used search term ‘Aged’ and ‘older adults’ in their search strategy, which are considered as electronic databases as person 65 years and above. Often studies on older adults have considered the later definition. So, it would be great if the authors could clarify this.
4. **Method - Expert panel:** The authors have considered an interprofessional expert panel for providing input in this review. I understand that they had a broad range of clinical and perspective related to the care of older adults. But for the methodological rigor the reader would benefit from knowing about the specific criteria for their inclusion.
5. **Result – Step 2 (page 12, L276):** The author state there were 19 studies reporting on mortality but have cited only 18 studies.
6. **Discussion – please consider the grammar – L384:** “…and those that did found…”
7. **Discussion:** The authors have raised a really good point that majority of reviews did not specifically aim to examine harms. While this is true, studies reporting on the causality of harm due to deprescribing is also limited. It is also true that unless evidence of harm associated with deprescribing is well established, deprescribing option should be open to patients. This is not a comment but a view.
